# RelCalc: symbolic evaluation of BWR theory relaxation rates in python, applications to TROSY effects in AX$_n$ spin systems

**DOI:** 10.1007/s10858-025-00479-6

**Published:** 2026-06-29

**Authors:** James Eaton, Daniel Stedman, Andrew J. Baldwin

**Affiliations:** 1 Physical and Theoretical Chemistry, Oxford University, Oxford, UK; 2 Kavli Institute of Nanoscience Discovery, Oxford University, Oxford, UK

**Keywords:** Relaxation rates, Redfield theory, BWR theory, NMR, MethylTROSY, TROSY, Long-lived states

## Abstract

Relaxation phenomena greatly influence magnetic resonance experiments, from setting repetition rates and measurement times through to measuring internal motion of molecules. RelCalc provides a convenient and efficient means for symbolic and numerical evaluation of relaxation rates using Bloch, Wagness and Redfield (BWR) theory. Users supply interactions within a spin system arranged appropriately in the molecular frame. The effects of optional fluctuations around a symmetry axis, and then under either isotropic or anisotropic global tumbling are then computed. Relaxation rates linking pairs of Liouvillian basis operators are then returned symbolically, formatted as a sum of weighted reduced spectra density functions. Parameters are then supplied to replace symbolic quantities for values for rapid computation of numerical rates. We demonstrate the utility of the software by exploring relaxation interference (TROSY) effects and ’long-lived states’ in $X_2$ and $AX_n$ systems, which, for *n*=1,2, and 3, describe NH, NH$_2$ and CH$_3$ groups in proteins, and small molecules with n=4,6,8,12 and 20 arranged as platonic solids. The implementation is highly efficient with 65,536 relaxation rates required to perform complete Liouvillian simulation of $AX_3$ methyl groups rotating about a symmetric axis in the presence of adjacent static ‘external’ spins being symbolically calculated in a few seconds on a single processor on a 2021 laptop. From this result, a complete set of numerical rates are then computed in under one second. RelCalc is implemented in python and is freely available.

## Introduction

Relaxation rates play pivotal roles in all aspects of magnetic resonance experiments. Starting from the introduction of the time-scales $T_1$ and $T_2$ by Bloch (1946), relaxation rates set the time-scale and recovery periods for measuring free induction decays (FIDs) Cavanagh et al. (2007), provide a quantitative means to experimentally characterise molecular dynamics (Reid Alderson et al. 2019; Juárez-Jiménez et al. 2020; Mittermaier and Kay 2006; Philippopoulos et al. 1997; Scott 2007; Dedmon et al. 2005) and enable experiments for high molecular weight complexes by solution NMR (Pervushin et al. 1997; Jason 2003). Relaxation, the loss of coherence and the return to thermal equilibrium is caused by incoherent motion where individual spins within an ensemble experience time dependent fluctuating magnetic fields. Bloch, Wagness and Redfield (BWR) theory (Wangsness and Bloch 1953; Redfield 2010) takes interactions in the molecular frame, evaluates the stochastic effects of random motion and returns relaxation rates.

Calculation of relaxation rates remains a challenging endeavour. Symbolic expressions are available in the literature, including X$_2$ (Malcolm and Levitt 2012), AX (Redfield 2010; Woessner 1962), AMX, AX$_2$, AX$_3$ (Kay et al. 1992; Jason 2003) and AX$_4$ (Werbeck and Hansen 2014) spin systems. These have led to deep insights and enable experiments that push the size limits of solution NMR including transverse relaxation optimised spectroscopy (TROSY) Pervushin et al. (1997) and methyl-TROSY (Jason 2003) methods. New applications can require additional interactions to be considered, with relaxation rates between more states than may be directly stated in the literature. To meet this need, a range of programs exist for both numerical and symbolic relaxation rates (Kuprov et al. 2007; Hogben et al. 2011; Bengs and Levitt 2018). These typically rely on proprietary software, and require deep understanding of the underlying quantum mechanics to use them.

NMR observables such as relaxation rates are influenced significantly by underlying molecular motions. It remains highly non-trivial to map back and forth between measurements and the specific molecular trajectories that determine them. Such information is likely essential to understand how, for example, biomolecules such as enzymes function. Current methods remain largely qualitative, identifying mobile and static segments within molecules. To improve this on this, it will likely be necessary to take complete descriptions of underlying interactions in a molecule, examine precisely how these are modulated by local and global sources of motion to accounting for all forms of auto and cross correlated relaxation encountered in a given experiment. The results of such an analysis need to be in a form that allows the dynamical trajectories to be robustly extracted.

To take a step towards this goal, we have developed RelCalc, a python based software for both symbolic and numerical calculation of relaxation rates. The software is designed to allow a user to focus on the chemical detail of setting up the relevant interactions in the molecular frame. It allows a range of motional models, both local and global to produce relaxation rates characterised by a minimal number of parameters. It is optimised to arrange the relaxation rates in a manner that emphasise useful features such as TROSY effects. By taking advantage of a variety of symmetries inherent to the problem, RelCalc’s implementation of a BWR computation is highly efficient, and once performed expressions can be retained for rapid re-computation. This renders it suitable for both simulation of spin physics, and fitting relaxation data.

A series of templates have been produced, released with the software, that allow a new user to both perform the computations of spin systems described in this manuscript and move beyond this, and we refer the reader to these. These enable computation of relevant relaxation rates in a form suitable for data analysis, avoiding the requirement to understand precisely how the relaxation rates were computed. Detailed understanding of relaxation theory is not required to use RelCalc, indeed the theory for computations of relaxation rates can be daunting for chemists and biochemists. We emphasise that the following sections are intended to provide the interested user insight into how the computations are performed and that deep understanding is not necessary to ‘use’ RelCalc to analyse experimental data.

RelCalc computes relaxation rates in the same way as a human (Cavanagh et al. 2007). A detailed derivation is provided (Appendix A) that arranges the theory into a form amenable to computation. In brief, the Redfield master equation describes how a system (described by a density matrix, $\rho$) will evolve in time, in the laboratory frame:

1 $$\begin{aligned} \frac{d\rho }{dt}={\hat{\hat{\mathcal {H}}}}^0\rho -{\hat{\hat{\Gamma }}}\left( \rho -\rho _{eq}\right) . \end{aligned}$$

${\hat{\hat{\mathcal {H}}}}^0$ accounts for the coherent evolution under interactions such as isotropic chemical shift and scalar coupling. The relaxation super-operator ${\hat{\hat{\Gamma }}}$ (Eq. A1) determines how random motion can cause a weakly perturbing Hamiltonian $\mathcal {H^\textrm{1}}$ to drive the system back to thermal equilibrium. Relaxation rates are conveniently understood in Liouvillian space, where $\rho = \sum _ic_i\rho _i$, a sum of time independent ortho-normal basis operators $\rho _i$ and time dependent coefficients $c_i$. The co-efficients $c_i$ form a state vector $c=(c_1,c_2,...)$, whose Eq. of motion is $\frac{d c}{dt}= -L c$ Allard et al. (1998). The elements of the Liouvillian matrix $L_{st}$ describe how magnetisation evolves between pairs of operators *s* and *t*. Each element has two components $L_{st}=H_{st}+R_{st}$ where

2 $$\begin{aligned} H_{st}=\langle \rho _s| {\hat{\hat{\mathcal {H}}}}^0 \rho _t\rangle , \end{aligned}$$

describes coherent evolution, and

3 $$\begin{aligned} R_{st}=\langle \rho _s| {\hat{\hat{\Gamma }}} \rho _t\rangle . \end{aligned}$$

is a relaxation rate, a real scalar value that describes either cross relaxation between states $\rho _s$ and $\rho _t$ or auto-relaxation if $s=t$. The states $\rho _s$ and $\rho _t$ are normalised density matrices which can be entered explicitly as matrices, or symbolically as operators, such as $H_x$ or $H_xC_z$. For a single spin-$\frac{1}{2}$, a convenient complete basis is formed from the normalised form of the Pauli density matrices $\rho _x$, $\rho _y$, and $\rho _z$ together with the identity *E*. The time-scales $T_1$ and $T_2$ from Bloch’s original work can be computed using Eq. 3 from $\frac{1}{T_1}=R_1 = R_{z,z} = \langle \rho _z| {\hat{\hat{\Gamma }}} \rho _z\rangle$ and $\frac{1}{T_2}=R_2 = R_{x,x}=R_{y,y}= \langle \rho _x| {\hat{\hat{\Gamma }}} \rho _x\rangle$.

**Fig. 1:**
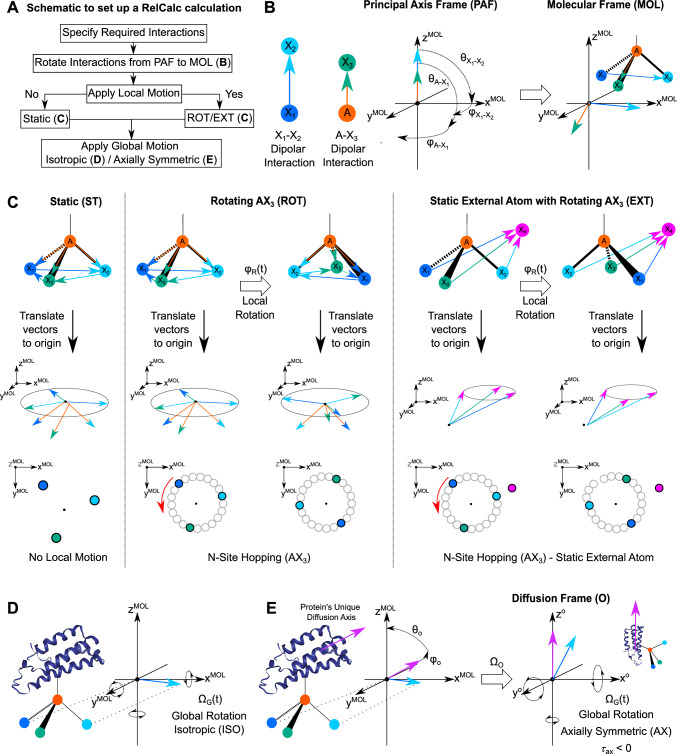
(**A**) A flowchart describing how the perturbing Hamiltonian $\mathcal {H}^1$ is set up in RelCalc (Sect. 2). (**B**) Each interaction is rotated from its initial orientation in the principal axis frame (PAF) on the positive *z* axis into the molecular frame by a polar rotation of $\theta$ about the *Y* axis and an azimuthal rotation of $\phi$ about the *Z* axis. This is illustrated for the $X_{1}$-$X_{2}$ and *A*-$X$ dipolar interactions in an $AX_{3}$ group. (**C**) Comparison of the three internal motional modes (ST/ROT/EXT) supported by RelCalc, illustrated by an $AX_3$ group. Static (ST) keeps all nuclei static in the molecular frame. Rotating (ROT) allows motion about the symmetry axis, which can either be *N* site hopping or diffusive, where motion leads to no variation in the interaction strength (distances for dipolar are held constant). External (EXT) covers the case where a group undergoing motion is dipolar coupled to an external group, and the distance between nuclei varies with time. (**D**) Global tumbling can either be isotropic or (**E**) axially symmetric. In the latter case, it is necessary to rotate the molecular frame into the diffusion frame by the angle $\theta _O$, where the unique axis for tumbling ($\tau _\parallel$) is initially aligned with the positive *z* axis

The relaxation super-operator ${\hat{\hat{\Gamma }}}$ (Eq. A1) requires a perturbing Hamiltonian $\mathcal {H^\textrm{1}}$ that describes all relevant interactions (e.g. quadrupolar, dipolar, CSA), arranged appropriately in a molecular frame to reflect a given problem (Fig. 1). To perform a BWR computation, pairs of interactions in the system are assessed to ascertain the degree to which they can cause inter-conversion between states *s* and *t* under the provided motional model (Eq. A20), and final relaxation rate has the following schematic form:

4 $$\begin{aligned} R_{st}=\sum _\gamma \kappa _\gamma J_\gamma \left( \tau _\gamma ,\omega _{\gamma }\right) . \end{aligned}$$

Each term in the sum is weighted by $\kappa _\gamma$ whose magnitude will depend on the strengths of the two contributing interactions (Table 1), arrangement of interactions in the molecular frame and a numerical symmetry factor. These are combined with a reduced spectral density *J* that describes the probability of frequency $\omega _\gamma$ being generated on time-scale $\tau _\gamma$ (Appendix B3),

5 $$\begin{aligned} J(\tau ,\omega )=\frac{\tau }{1+\omega ^2\tau ^2}, \end{aligned}$$

which is a Lorentzian function that takes the value $\tau$ at $\omega =0$, tending to zero as $\omega$ increases. RelCalc retains any numerical parts of $\kappa$ with symbolic constants that describe interaction strengths (Table 1) and Legendre polynomials $P_q^{(r)}$ that orient interactions in the molecular frame (Table 1). In the case of anisotropic diffusion (Fig. 1), functions that orient the molecular frame with respect to the diffusion frame are also required (Table 11). As the final relaxation rates are saved in this schematic form, the constants can be re-computed with new values very efficiently, rendering the output highly amenable to data analysis.

**Table 1:** *Left: *RelCalc allows dipolar, quadrupolar or CSA terms associated with individual spin(s)

	$a_k$	$A_k$	*q*	$P_q^{(1)}$	$P_q^{(2)}$
Dipolar	$-\frac{\hbar \gamma _A\gamma _X \mu _{0}}{4\pi }$	$\frac{1}{r^3}$	0	$\cos \theta$	$\frac{1}{2}(3\cos ^2\theta -1)$
Quadrupolar	$\frac{eV Q}{\hbar }$	1	1	$\sin \theta$	$3\cos \theta \sin \theta$
CSA	$\frac{1}{3} B_0 \gamma \Delta\delta$	1	2		$3\sin ^2\theta$

These can be used as templated for any bilinear, quadratic or linear interaction. Each is supplied to RelCalc with a symbolic interaction constant. During numerical evaluation, additional parameters need to be supplied; a distance in metres (dipolar), differences in the chemical shift tensor in ppm (CSA) or differences in the electric field gradient tensor in atomic units (quadrupolar, SI unit of $V m^{-2}$, supplied in atomic units (Hartrees/electron charge/Bohr radius$^2$) and converted internally to SI) such that the resulting contribution to a relaxation rate has units rad s$^{-1}$. Nuclei types to characterise gyromagnetic ratios (in rad s$^{-1}$ T$^{-1}$) or nuclear cross-sections, *Q*, (in Barns, 100 fm$^2$) are specified and values are recalled from an internal library (Pyykkö 2018) (quadrupolar). For the quadupolar interaction, a factor of $\frac{1}{4S(2S-1)}$ is automatically applied to the result taking the quantum number *S* from the specified spin system. A static field $B_0$ (in Teslas) is set by supplying the corresponding proton Larmor frequency (in MHz) of the spectrometer of interest. The dipolar interaction is axially symmetric and requires only one contribution per interaction. The constant $A_k$ used in the spectral density function is set to $\frac{1}{r^3}$ (which may vary with time, EXT, Fig. 1) (Appendix A1). In studies involving relaxation rates the constants parameterising the CSA and quadrupolar interactions are typically used as fitting parameters. In cases where a Cartesian chemical shift tensor, $\delta$ (CSA) or an electric field gradient tensor, *V* (quadrupolar) are known, the values can be supplied as follows (Appendix A1). The CSA interaction can contain two symmetric axially symmetric terms, $\Delta\delta ^{ax}=\delta _{zz}^{(2)}-\delta _{yy}$ and $\Delta\delta ^{ax2}=\delta _{xx}-\delta _{yy}$ where $\delta _{ii}$ are the three eigenvalues of the rank 2 tensor decomposition. The second axially symmetric term requires additionally an angle that orients the interaction in the xy plane, $\phi _{ax2}$. An asymmetric contribution can also be specified ($\Delta\delta _{as}=\sqrt{\delta _{xy}^2+\delta _{xz}^2+\delta _{yz}^2}$) with values derived from the rank 1 tensor decomposition, with angles that arrange it in the PAF ($\theta _{as}$ and $\phi _{as}$), and the constants $a_k=\gamma B_0\Delta\delta _{as}$. The quadrupolar interaction can contain two axially symmetric terms derive from the three eigenvalues $V_{ii}$ of the electric field gradient tensor, where $V_{ax}=\frac{1}{3}\left( V_{zz}-V_{yy}\right)$ and $V_{ax2}=\frac{1}{3}\left( V_{xx}-V_{yy}\right)$. The second term can be supplied with an angle $\phi _{ax2}$ that arranges the tensor in the xy plane. All contributed terms are then rotated into the molecular frame by angles $\theta _R$ and $\phi _R$. All definitions for relevant interactions are provided symbolically in the PDF report for reference. **Right:** Arranging the interaction in the molecular frame requires evaluation of Legendre polynomials $P_q^{(r)}$ of rank *r* computed from $\cos \theta _R$. If the angle is provided symbolically, final expressions will be in terms of symbolic Legendre polynomials, which can then be evaluated numerically from a specified angle $\theta _R$

As an example, consider an isolated $^{13}CH_3 (AX_3)$ methyl group allowed to ‘hop’ around its three-fold symmetry axis in a molecule with time constants $\tau _{HOP}$ in a molecule undergoing axially symmetric anisotropic tumbling. To analyse this situation, an input script is provided, allowing a user to alter the parameters repeatedly as needed. Here we compute the auto-relaxation rates of the ‘outer lines’ of a $^{13}C$ quartet, associated with the Liouvillian operators for $\hat{C}_+\hat{H}_{\alpha }\hat{H}_{\alpha }\hat{H}_{\alpha }$ and $\hat{C}_+\hat{H}_{\beta }\hat{H}_{\beta }\hat{H}_{\beta }$ that have $^1$H projected angular momenta values of $\pm \frac{3}{2}$. The resulting relaxation rate is of the schematic form (Eq. 4). Expressing this rate in the macromolecular limit often used for biological molecules (retaining frequencies in $\omega _A$ and smaller, and assuming $\tau _c>> \tau _{\textrm{HOP}}$) leads to the following result containing 45 terms that, to our knowledge, has not been published previously:

6 $$\begin{aligned} & R^{AX_3}{\pm \frac{3}{2}} = \sum _{q_0=0}^2 \frac{1}{5}M^{q_0}{0,0} \left( 3dP_0 \pm 2c_A\right) ^2\nonumber \\ & \quad \left( \frac{\tau c}{1+\frac{q_0^2}{6}\left( \frac{1}{\sigma }-1\right)}\right. \nonumber \\ & \quad \left. +\frac{3}{4} J\left( q_0,0,\omega _A\right) \right) + \tau _{\textrm{Hop}} \left( M^{q_0}{1,1}P_1^2\left( \frac{3}{5} c_X^2 + d^2 \right) \right. \nonumber \\ & \quad \left. + \frac{1}{4}M^{q_0}{2,2}\left( \frac{3}{20} \left( 3e \pm 2c_XP_2\right) ^2 + d^2P_2^2 + \frac{27}{5} e^2 \right) \right. \nonumber \\ & \quad \left. + \cos (3\phi _M) M^{q_0}{-1,2}P_1 \left( d^2P_2 - \frac{9}{10} ec_X \right) \right) \end{aligned}$$

Axially symmetric CSA interactions on both C and H nuclei are included (described by constants $c_A$ and $c_X$ respectively, Table 1) with CH and HH dipolar interactions (constants *d* and *e* respectively, Table 1). The shape factor $\sigma =\frac{\tau _{\parallel }}{\tau _c}$ describes the relative size of the unique global tumbling time ($\tau _c$) to the degenerate tumbling time ($\tau _\parallel$). The 2nd order Legendre polynomials $P_q$ are defined by a single angle $\beta$, between the positive *z* axis and the CH bonds, and the $M^{q_0}_{q,q^*}$ coefficients orient the molecular frame with diffusion frame (Table 11, Eq. B14), parameterised by the further angles $\theta _O$ and $\phi _O$.        

Although the expression is complicated, it can provide substantial insight into how the spin system behaves in experiments. The CH dipolar and C CSA interactions interfere, as for $+\frac{3}{2}$ the CH dipolar term $3dP_0$ combines constructively with the carbon CSA term, $2c_A$ increasing the relaxation rate. By contrast, for $-\frac{3}{2}$ state, the two terms interfere destructively, reducing the relaxation rate (Flemming Hansen et al. 2009). Here we also see the HH dipolar and H CSA contributions also exhibiting relaxation interference. This spin system is explored numerically in more detail (Sect. 3.5), where limitations associated with the specific assumption of the macromolecular limit are discussed. While symbolic expressions contain physical insight, the expressions are cumbersome. Relcalc allows a user, should they wish, to keep these hidden, concentrating instead only on the numerical values used to compute the rates.    

Finally, RelCalc produces a PDF report where in the constant terms, irrational numbers are converted where possible into rational fractions, and attempts are made to identify relaxation interference effects by ‘completing the square’ (e.g. Eq. 6). All results in this manuscript were produced in this fashion, copied from the reports. The symbolic expressions are retained in a form that supports rapid re-calculation, rendering the engine highly suitable for numerical simulations. A sample input script is provided (Appendix I), and all scripts used for the results sections are included with the software download.

In what follows, we first describe the symbolic language the software uses to characterise spin systems and then present both symbolic and then provide numerical relaxation rates from a range of systems of experimental interest. We refer a new user to the examples with the software to gain familiarity if he following sections are challenging to assimilate.

## How to setup a computation

A detailed manual and examples are provided with the software. To setup a computation requires first the definition of a spin system, a description of the various interactions of interest in the molecular frame and a motional model. Basis operators $\rho _s$ and $\rho _t$ then need to be supplied, where string based names are linked to either symbolic or matrix representations of the operators. The final relaxation rates are stored as a dictionary (Eq. 4) indexed by the strings that describe $\rho _s$ and $\rho _t$. These can be saved, from which numerical rates can be rapidly re-evaluated as many times as required with new parameters. Plots are then created in 3D that show the orientation of all interactions, in the molecular frame.

Both the operators describing the interactions and basis operators for relaxation rates are supplied as text strings that are interpreted by RelCalc to compute the relevant spin physics. This has the advantage that all the symmetries and tricks used by a human to avoid doing vast quantities of matrix algebra can be hard-coded into the computation for substantial efficiency gains. In a practical case, the input from a user is minimal, requiring a few text strings describing the spin system and its arrangement in the molecular frame, together with the basis operators of interest for relaxation rates.

### Define a spin system

A spin system is first supplied, eg *CH*2*D* or *CFH*3, where each letter is a nucleus and the number gives the number of repeats. The spin of each nucleus will be assumed to be $\frac{1}{2}$ unless otherwise specified. When referring to operators or introducing operators for rate calculations, spins will be numbered by counting from the left where for example for $CH_2D$, the spins will be labelled as *C*1, *H*2, *H*3 and *D*4 when introducing specific basis operators $\rho _s$ and $\rho _t$ (section 4).

### Define $\mathcal {H^\textrm{1}}$

Interactions need to be specified that make up the perturbing Hamiltonian in the molecular frame (Fig. 1, Appendix A1). These are selected from CSA, dipolar and quadrupolar interactions, and assigned to the relevant spin(s). In the simplest case where each interaction is axially symmetric, a symbolic constant for the interaction strength is provided $A^{\textrm{ax}}$, with a pair of interaction specific polar angles $\theta _R,\phi _R$ that rotate the tensor from its principle axis frame (PAF, usually the *z* axis) into the molecular frame. The angles can be symbolic, in which case the results will be parameterised by Legendre polynomials, or numerical constants (Table 1).

More generally, all relevant interactions are described by traceless spatial tensors *A* (Appendix A1) that can be decomposed into 3 independent components. While the dipolar interaction is inherently axially symmetric (requiring one constant), CSA and quadrupolar interactions are not. The quadrupolar interaction can have an additional symmetric contribution $A^{\textrm{ax2}}$ that can be added with an angle that orients it in the xy plane $\phi _{ax2}$. The CSA interaction may require both axially symmetric components, and also an anti-symmetric part $A^{\textrm{as}}$, that requires a further a pair of polar angles $\theta _{as},\phi _{as}$ for orientation in the PAF. All three terms are then rotated from the PAF by $\theta _R,\phi _R$ about the YZ axes, into the molecular frame. An interaction tensor maximally requires three interaction strengths, three angles that align the tensor in the PAF and two angles that orient the tensor in the molecular frame, amounting to 8 independent degrees of freedom. The symmetric parts alone require 5 parameters, and the axially symmetric component alone requires 3.

The individual two spin operators that define the Hamiltonian are either retained symbolically (for spins $\frac{1}{2}$) or their matrix representation is automatically constructed, as the operators for single spins with +, − or *z* are defined for any spin.

### Define a motional model

Each interaction requires a local motion model to be supplied and the overall computation requires a global tumbling model (Fig. 1). The effect of these is to split the overall spectral density function (Table 12) into a sum of model specific individual reduced spectral density functions, each with a characteristic amplitude, frequency and time-scale (Eq. 5). The spectral density functions for all models considered here are derived explicitly (Appendix B1) via their correlation functions (Appendix B1).

One of six motional models can be selected by combining 2 types of global and 3 types of local motion (Fig. 1); i) isotropic tumbling with no local motion (ISO ST, Furry (1957); Redfield (2010)), ii) axially symmetric tumbling and no local motion (AX ST, Favro (1960); Woessner (1962)), iii) isotropic tumbling with spins rotating about a symmetry axis in a manner that keeps the distance between the spins constant (ISO ROT, Woessner (1962); Wallach (1967), iv) axially symmetric tumbling with spins rotating about a symmetry axis in a manner that keeps all distances constant (AX ROT, Wesley (1968); Woessner et al. (1969); Robert (1976)), v) isotropic tumbling where there is rotation about a symmetry axis that causes variable distances during the internal motion (ISO EXT, Dellwo and Wand (1993)), a mode suitable for treating NOEs between methyl protons and an adjacent group (Dellwo and Wand 1993) and vi) axially symmetric tumbling in a similar situation, which we have been unable to find in the literature (AX EXT).

RelCalc accommodates these models within a single framework using a generic time-scale function (Appendix B1):

7 $$\begin{aligned} \tau (q_0,q,r)=\left( \frac{1}{\left( 1+2\delta _{r,1}\right) \tau _c}+\frac{q_0^2}{\tau _{ax}}+\frac{1}{\tau _{\textrm{loc}}(q)}\right) ^{-1}. \end{aligned}$$

that itself is parameterised by three integers, $q_0$, *q* and *r* that arise when specific pairs of operators are analysed during the computation. $q_0$ takes the values 0,1,2 that modifies the time-scale for axially symmetric global tumbling (0 for isotropic global tumbling), *q* describes the local motion about a symmetry axis (Fig. 1) and the tensorial rank *r* is 1 for anti-symmetric components, and 2 for symmetric. The introduction of $\delta _{r,1}$ is to reduce $\tau _c$ by a factor of 3 when antisymmetric interactions are under consideration. $\tau _c$ is the isotropic global tumbling time (or the degenerate rotational time-scale in the axially symmetric case), the unique rotational tumbling time-scale $\tau _{ax}=6\tau _c\left( \frac{1}{\sigma }-1\right) ^{-1}$ where the shape factor $\sigma =\frac{\tau _{\parallel }}{\tau _c}$ and $\tau _{\parallel }$ is the tumbling time of the unique axis ($\sigma$ > 1 the motion is oblate and $\sigma$ < 1, the motion is prolate). The local motion time-scale describing rotation about the symmetry axis (Appendix C) is

8 $$\begin{aligned} \frac{1}{\tau _{\textrm{loc}}(q)}=2 k \left( 1-\cos \left( \frac{2\pi q}{N}\right) \right) , \end{aligned}$$

and is parameterised by the integer *q* where *k* is the rate constant describing moving between adjacent sites. This definition allows for both diffusive motion in a ring, and ‘hopping’ between *N* sites. For a hopping model, it is common to define local motion timescale $\frac{1}{\tau _{\textrm{HOP}}}=\frac{1}{Nk}$. If we take the limit $N\rightarrow \infty$, we obtain continuous diffusion in a ring where $\frac{1}{\tau _{\textrm{loc}}(q)}=\frac{2q^2 }{\tau _{\textrm{Diff}}}$ where the 1D diffusion time scale is $\tau _{\textrm{Diff}}=\frac{1}{k}\left( \frac{N}{2\pi }\right) ^2$, the lifetime per unit squared radian ‘hopped’ (Appendix C). For $N=2,3,4$ there are small differences between an *N*-state hopping model, and a freely diffusing case. For $N>4$, the two models are indistinguishable (Appendix B1). Either ‘hopping’ or ‘diffusive’ can be selected for the local motion in RelCalc.

The generalised time-scale reduces to simple values. Where there is no motion about a symmetry axis in the molecular frame, $\tau _{\textrm{loc}}(q)^{-1}=0$ and the generalised time-scale becomes independent of *q*. In the case of isotropic tumbling, $\sigma =1$, $\tau _{ax}^{-1}=0$ and dependence on the integer $q_0$ is removed. For isotropically tumbling with no local motion, both $q_0$ and $q=0$, and $\tau (0,0)=\tau _c$. In the slow tumbling (macromolecular) limit where $\tau _{ax},\tau _c>> \tau _{\textrm{loc}}(q)$ and for terms where $q \ne 0$, $\tau (q_0,q)\approx \tau _{\textrm{loc}}(q)$ (Fig. 9), and so depend only on the local motion. All relaxation rates are fundamentally parameterised by up to 3 values: $\tau _c$, $\sigma$ if anisotropic diffusion is required and either $\tau _{\textrm{HOP}}$ or $\tau _{\textrm{Diff}}$ if there is local motion.

When considering dipolar interactions where distance can vary during the motion (ISO EXT and AX EXT, Fig. 1), the spectral density function is left as a symbolic factor, which can be evaluated numerically (Appendix D).

### Specifying operators of interest

Following the definition of $\mathcal {H^\textrm{1}}$ and after pairs of terms have been identified that can drive relaxation, the final task is to supply lists of operators for which all combinations of $\rho _s$ and $\rho _t$ will be drawn, selected to be relevant for a given application. Operators can be supplied to RelCalc symbolically. For example, if the spin system is CH2D, operators can be supplied as strings such as ‘C1xH2yD4z’. Matrix representations will be automatically generated, provided the state for each spin is specified in either the Cartesian (*xyzE*) or shift ($+/-$, specified as *p* or *m* respectively) bases. For spins-$\frac{1}{2}$, the symbols $\alpha$ and $\beta$ will also be interpreted automatically as single element density matrices. For a more general case, any matrix for $\rho _{s/t}$ can be constructed manually and linked to an arbitrary string identifier. Moreover, composite operators can be constructed, by symbolically specifying linear combinations and linked the result to an arbitrary string, used to identify specific entries for $\rho _{s/t}$. For example, in a three spin computation, the arbitrary string ‘pab’ can be set to represent the following two operators, also specified to RelCalc as strings, ‘+1.0 C1pH2aH3b’ and ‘+1.0 C1pH2bH3a’, corresponding to transverse carbon magnetisation, and all permutations of a the adjacent two protons that have projected angular momentum of zero. Such a term would represent the central transition of the triple in a one pulse 13 C NMR experiment. This composite operator identified above as strings would be described more formally in terms of the underlying wavefunctions or equivalently as outer products of single spin basis basis operators as

9 $$\begin{aligned} & \texttt {'pab'}=\left| \alpha \alpha \beta \rangle \langle \beta \alpha \beta + \alpha \beta \alpha \rangle \langle \beta \beta \alpha \right| \nonumber \\ & \quad = C_+^1 H_\alpha ^2 H_\beta ^3 + C_+^1 H_\beta ^2 H_\alpha ^3. \end{aligned}$$

All operators used for $\rho _{s/t}$ are normalised prior to a relaxation rate computation. For spins-$\frac{1}{2}$, the single spin identifiers $xyz+-ab$ are insufficient to describe all possible states in a multi-spin ensemble can form. For spins $>\frac{1}{2}$, the single spin operators $xyz+-$ will be interpreted automatically but should more be required then a bespoke definition can be supplied. For a spin 1 for example, a complete set of single spin density matrices can be found in e.g. Allard and Härd (2001).

RelCalc will not automatically apply a ‘Redfield Kite’ when constructing relaxation rates. The effect of a non-zero cross-relaxation rate between two operators whose characteristic evolution frequency in the laboratory frame differs by MHz will in practice be negligible, and this factor is not accounted explicitly by RelCalc. Care should be taken to ensure that such rates are manually set to zero or otherwise corrected if the rates are used for Liouvillian simulations. Functions have been provided that will automatically set relaxation rates to zero, provided *s* and *t* have been supplied in the shift basis (using $+-$ terms for single spin basis elements), allowing relaxation rates between terms of the same coherence order from the same nuclei to be retained, and the others set to zero.

In certain applications, individual relaxation rates are sufficient for data analysis. More generally, multiple rates describing all possible transfers between operators of interest to assemble an evolution matrices *L*. A complete set of Liouvillian basis matrices for *N* spins, whose total spin angular momentum is $S_i$ will require $C=\left( \prod _{i=1}^N(2S_i+1)\right) ^2$ ortho-normal basis operators. The dimension of the corresponding evolution matrix *L* will be $C\times C$, requiring $C^2$ total relaxation rates. For a $^{13}$CH$_3$ methyl group for example, $C=256$ leading to (in principle) 65,536 relaxation rates (of which many are effectively zero in the rotating frame).

It is often desirable to work with operators constructed from wavefunctions that form the eigenbasis of $\mathcal {H}_0$. In such a basis coherent evolution matrix will be diagonal, $H_{st}=i\omega _{s} \delta _{s,t}$, where $\omega _s$ is the evolution frequency of operator $\rho _s$ and $\delta _{s,t}$ is the Kronecker delta that ensures that all off-diagonal elements are zero. In the absence of relaxation, coefficients will evolve as $c_s(t)=e^{-i\omega _{s}t}c_s(0)$. In this case, applying a ‘Redfield Kite’ amounts to retaining off diagonal relaxation rates only if the evolution frequencies are identical.

When considering degenerate spins, such as the three protons in a CH$_3$ group, two additional approaches are provided to assemble basis elements in RelCalc. The first is suitable for evaluating simple pulse/acquire experiments, and the second is more general and uses group theory to explicitly compute the eigenbasis, and uses these basis wavefunctions to construct a complete set of Liouvillian basis matrices. Examples of both are provided in the results section.

For the first case, consider a simple pulse/acquire NMR experiment on the *A* spin of an $AX_n$ system of spins-$\frac{1}{2}$. The density matrix after the pulse is given by $A_+$. This can be written more explicitly as $A_+ \equiv A_+ \prod _{j=1}^n \otimes ( X^j_\alpha + X^j_\beta )$ where for the passive *X* spins, $X^j_\alpha + X^j_\beta =E^j$, the identity. The binomial expansion reveals the density matrix to be a linear combination of *N*! individual terms. These can be grouped into $N-1$ unique evolution frequencies given by $\nu _A+ mJ$ where $m=\frac{1}{2}(n_\alpha -n_\beta )$ and $n_\alpha$ and $n_\beta$ are the total number of $\alpha$ and $\beta$ elements in the term. Each of the $N-1$ operators will be $\frac{(n_\alpha +n_\beta )!}{n_\alpha !n_\beta !}$ degenerate. In the absence of relaxation, the intensities of any multiplet in a pulse/acquire NMR spectrum will follow Pascal’s triangle (Appendix H). Such a basis is automatically constructed by RelCalc on request. For an $AX_2$ spin system, this will construct operators indexed as ‘paa’, ‘pab’ and ‘pbb’ where ‘pab’ state for a CH2 spin system would be the normalised linear combination of $C^1_+H^2_\alpha H^3_\beta$ and $C^1_+ H^2_\beta H^3_\alpha$. The indices can be used as inputs for $\rho _{s/t}$ to get the corresponding relaxation rates. We refer to this as the ‘single quantum Zeeman basis’ in the manuscript and is suitable if the passive spins *X* can be described purely by populations (no coherences).

For a complete description of a system containing degenerate spins, more work is needed to form the eigenbasis of $\mathcal {H}^0$, which will will contain terms such as $I_1 \cdot I_2$ (the ‘strongly J-coupled Hamiltonian’, Eq. H2). In RelCalc we first use methods from group theory (Corio 1966) to find the underlying wavefunctions termed an ‘irreducible basis’, expressed as linear combinations of isolated spin basis wave functions (Corio 1966; Müller et al. 1969; Kay et al. 1992; Jason 2003; Malcolm and Levitt 2012; Werbeck and Hansen 2014). The wavefunctions obtained from such an analysis (Appendix H) necessary follow the rules for quantum mechanical addition of angular momenta, and so naturally group into a series of manifolds each with 2J+1 members, with a total angular momentum *J* and individual projected angular momenta of $m_j$ ranging from $+J$ to $-J$. For *N* degenerate spins half, this approach will generate $2^N$ wavefunctions, where $N+1$ span a symmetric representation with maximum total angular momentum, $J=\frac{1}{2} N$. These are numbered and automatically tabulated in the PDF report. RelCalc then uses these wavefunctions via ket/bra products to construct the $4^N$ Liouvillian operators $\left| i\rangle \langle j \right|$ required to form a complete basis. These can be further combined with single spin operators for the other spins. Strings such as ‘p4-4’ for an $AX_2$ spin system will then correspond to the basis operator $A_+ \left| 4\rangle \langle 4 \right|$.

The irreducible representation provide physical insights into spin dynamics. For example, Hamiltonians for RF pulses in the rotating frame take the form of ladder (+/-) operators, which are able to cause transitions within a manifold but not between them, and the dipolar interaction tends to rapidly relax states with high projected angular momentum. Transitions can still occur between manifolds, and are ominously termed ‘forbidden’, caused either through evolution of interactions under $\mathcal {H}^0$ in carefully designed pulse sequences (Müller et al. 1969; Malcolm and Levitt 2012), or through relaxation mechanisms (Tugarinov et al. 2007). Examples of which are presented in the results section.

### Evaluation of symbolic relaxation rates

After $\mathcal {H^\textrm{1}}$ has been defined (Appendix A1), and the operators $\rho _s$ and $\rho _t$ have been supplied, relaxation rates are evaluated. There are several features within the algorithm that are collectively responsible for the efficiency of RelCalc (Appendix G). To obtain a relaxation rate, a quadruple sum is performed (Eq. A20) over all pairs of interactions and their constituent operators each with a characteristic frequency $\omega$.

Only pairs of operator $\hat{O}$ of the same rank and characteristic frequency can drive relaxation (Eq. A24), and are retained with relevant symbolic constants (Table 12) and reduced spectral density functions (Eq. 5) relevant to the specified motional model.

A calculation can be restrained at this stage to the macromolecular limit, which significantly reduces the number of potential pairs of operators capable of driving relaxation, reducing the overall computation time. This approximation sets $\tau _c>>\tau _{\text {HOP/diff}}$. If diffusion is axially symmetric, the macromolecular limit also restraints $\tau _{ax}>>\tau _{\mathrm {HOP/diff}}$ which has the effect of setting $J(q_0,q,\omega )=\tau _m(q)$ if $q>0$, and if $q=0$, and only operators with characteristic frequencies $\omega \le \omega _C$ are retained (Flemming Hansen et al. 2009). This vastly reduces the number of contributing terms, and hence the complexity of the result (and the calculation time). Though useful, such approximations can erode accuracy of the final results (Figs. 4, 5).

Similarly, the software attempts to combine spherical harmonics arising from the orientation of elements in the molecular frame. Because sums of paired spherical harmonics *k* and *l* can be combined, taking the case where both are functions only of $\theta$ (Table 7), then

10 $$\begin{aligned} & \sum _{q=-r}^r Y_q^{k(r)}(\beta ) Y_q^{l (r)}(\alpha ) = \sum _{q=-r}^r y_qy_q P^{k,(r)}_q(\cos \alpha ) P^{l(r)}_q(\cos \beta )\nonumber \\ & \quad =P_0^{(r)}(\cos (\alpha -\beta )). \end{aligned}$$

If RelCalc detects terms with Legendre polynomials $$P_0^{k(r)}P_0^{l(r)}$$, then it attempts to replace them with $$P_0^{(r)}\cos (\alpha -\beta )- \sum _{q=-r}^r (1-\delta _{q,0}) y_qy_qP_q^{k(r)}(\cos \alpha ) P_q^{l(r)}$$$$(\cos \beta )$$, provided that it can find terms in $$P_q^{k(r)}P_q^{l(r)}$$ with matching reduced spectral density functions. Particularly for isotropic tumbling, this can greatly simplify results. The appearance of Legendre polynomials in a final relaxation rate could naively imply that the rate depends on the orientation of the molecular frame. By recombining pairs of Legendre polynomials with a new Legendre polynomial that depends only on the relative angle between interactions, then the dependence on the alignment of the molecular frame is removed. Cases where this is particularly helpful include $$k=l$$ where $$P_0^{(r)}(\cos (0))=1$$, and when analysing results from two independent parts of the symmetric tensor (such as for the quadrupolar interaction), as the angle between the two is always $$90^0$$, $$P_0^{(2)}(\cos (\frac{\pi }{2}))=-\frac{1}{2}$$. Including these substitutions at an early stage reduces the amount of symbolic work the program has to do.

Having identified pairs of operators $\hat{O}_{ki/lj}$ capable of causing relaxation with their numerical and symbolic components evaluated as far as possible, the final and most time consuming part is to evaluate selection factors that are traces of double commutators (Eq. A25),

11 $$\begin{aligned} S_{ki,lj}^{st}= \langle \rho _s| \left[ \hat{O}_{lj},\left[ \hat{O}_{ki}^\dagger , \rho _t\right] \right] \rangle \end{aligned}$$

where the notation *ki*/*lj* indexes each pair of interactions (*k*, *l*), and constituent operator *O* (*i*, *j*, Table 9).

In the case of spins-$\frac{1}{2}$, the commutators and the trace can be performed symbolically (Appendix G), which vastly reduces both the memory requirements as there is no need to store a matrix representation, and the overall computation times (Fig. 8) as the results are evaluated from a library. Otherwise, the operations are performed directly on matrix representations of the various operators (which can be stored in a sparse format). The non-zero values $S_{ki,lj}^{st}$ are combined with the retained numerical and symbolic factors, with appropriate reduced spectral density terms (Eq. 5), resulting in a relaxation rate $R_{st}$ of the expected form of Eq. (3).

For efficiency, the terms in $\mathcal {H^\textrm{1}}$ are grouped first by $\hat{O}_{ki}$, then $\hat{O}_{lj}$ in order to minimise commutator operations. The first commutator is evaluated, and the second is performed only if the first is non-zero. These are evaluated for each $\rho _t$ in the list. This value is then repeatedly re-used for each $\rho _s$ operator of interest. Following the trace operation, non-zero elements are stored as a list of spectral density functions together with relevant interaction constants and numerical factors in the form of Eq. 4.

### Numerical evaluation of relaxation rates

The elements that describe the relaxation rate (Eq. 4) are stored in a dictionary indexed by the strings linked to $\rho _s$, then $\rho _t$. For numerical evaluation, a second dictionary has to be populated whose symbolic indices are linked to the symbols used in the relaxation rates, which contain values that lets RelCalc numerically evaluate the relevant symbolic terms in the rates. A numerical relaxation rate is then obtained by summing over all elements in the list for a given $\rho _{s/t}$ pair, following replacement of symbolic terms by numerical factors.

During this process, Legendre polynomials are computed from specified angles, and reduced spectral density functions are computed from relevant global and local motional time-scales parameterised by motional variables such as $\tau _c$. Nucleus types are provided for mapping to internally known gyromagnetic ratios and for quadrupolar interactions, cross sections (Table 1).

In the case where the distances in a dipolar interaction vary with a local motion (‘EXT’, Fig. 1) the calculation further requires two spherical polar co-ordinates to be provided ($r,\theta ,\phi$), one for the static ’external’ spin in the molecular frame, and one for the initial position of the mobile ‘internal’ spin that will either hop or diffuse about the symmetry axis (Appendix B1). The values of the correlation functions are then computed numerically (Appendix D).

RelCalc notes all symbols required, and should prompt a user if it cannot compute a symbolic term with the provided information. Example input files for each of the examples are provided to serve as templates from which new calculations can be constructed. Relaxation rates can be rapidly re-evaluated once the symbolic result has been computed. The dictionaries containing the relaxation rates can be saved and re-used allowing the software to act as the kernel for an efficient numerical engine, or to optimise parameters that specify the relaxation rates when fitting to data.

### Presentation of results

As an option, a PDF report can be created using LaTex (see installation instructions). These summarise the constants required to define the various rates and symbolic expressions for the relaxation rates themselves. Following conventions in the literature, attempts are made to show numerical factors as rational fractions and to highlight the effects of relaxation interference, terms that are products of CSA (*c*) and dipolar (*d*) constants, can be factorised by completing the square (where $\alpha ,\beta ,\gamma$ are constants):

12 $$\begin{aligned} & \left( \alpha d^2 \pm \beta d c + \gamma c^2\right) J\left( n,\omega \right) = \left( \frac{\beta }{2\sqrt{\gamma }}d \pm \sqrt{\gamma }c\right) ^2J(n,\omega )\nonumber \\ & \quad + \left( \alpha -\frac{\beta ^2}{4\gamma }\right) d^2J\left( n,\omega \right) . \end{aligned}$$

Constructive (+) and destructive (-) interference between CSA and dipolar interactions can then be readily discerned, where often the square is inherently complete, where $\alpha -\frac{\beta ^2}{4\gamma }=0$.

The report can be suppressed when only numerical results are required, or where large numbers of relaxation rates are required. Functions are also provided to allow computation of rates as a function of correlation time, $\tau _c$, and plots of ‘Redfield Kites’ are produced that use colour to show auto- and cross-correlated relaxation rates on a grid. Figures showing relaxation rates in this manuscript were automatically generated by RelCalc, as were the summary tables (Tables 2, 3, 4, 5 and 6).

**Table 2:** Macromolecular limit relaxation rates for an $AX_2$ group automatically generated by RelCalc, considering AX dipolar, XX dipolar, A CSA and X CSA interactions, with the local motion being diffusive

Rate	$d^2P_0^2$	$d^2P_0^2$	$dc_AP_0$	$dc_AP_0$	$c_A^2$	$c_A^2$	$d^2P_1^2$	$d^2P_2^2$	$e^2$	$ec_XP_2$	$c_X^2P_1^2$	$c_X^2P_2^2$
	$\tau _c$	$J(0,\omega _A)$	$\tau _c$	$J(0,\omega _A)$	$\tau _c$	$J(0,\omega _A)$	$\tau _m$	$\tau _m$	$\tau _m$	$\tau _m$	$\tau _m$	$\tau _m$
$R_{A_{+}\beta _{2}}$	$\frac{4}{5}$	$\frac{3}{5}$	$-\frac{8}{5}$	$-\frac{6}{5}$	$\frac{4}{5}$	$\frac{3}{5}$	$\frac{1}{3}$	$\frac{1}{20}$	$\frac{27}{160}$	$-\frac{3}{40}$	$\frac{2}{5}$	$\frac{1}{40}$
$R_{A_{+}\alpha \beta }$					$\frac{4}{5}$	$\frac{3}{5}$	$\frac{4}{5}$	$\frac{1}{48}$	$\frac{9}{160}$		$\frac{2}{5}$	$\frac{1}{40}$
$R_{A_{+}\beta _{2}}$	$\frac{4}{5}$	$\frac{3}{5}$	$\frac{8}{5}$	$\frac{6}{5}$	$\frac{4}{5}$	$\frac{3}{5}$	$\frac{1}{3}$	$\frac{1}{20}$	$\frac{27}{160}$	$\frac{3}{40}$	$\frac{2}{5}$	$\frac{1}{40}$
$R_{A_{+}\alpha \beta ,A_{+}\beta _{2}}$							$\frac{1}{\sqrt{200}}$	$\frac{1}{\sqrt{51200}}$	$-\frac{9}{\sqrt{51200}}$	$\frac{3}{\sqrt{3200}}$	$-\frac{\sqrt{2}}{5}$	$-\frac{1}{\sqrt{3200}}$
$R_{A_{+}\alpha _{2},A_{+}\beta _{2}}$									$-\frac{9}{80}$			
$R_{A_{+}\alpha \beta ,A_{+}\alpha _{2}}$							$\frac{1}{\sqrt{200}}$	$\frac{1}{\sqrt{51200}}$	$-\frac{9}{\sqrt{51200}}$	$-\frac{3}{\sqrt{3200}}$	$-\frac{\sqrt{2}}{5}$	$-\frac{1}{\sqrt{3200}}$

Relaxation rates are obtained by multiplying the constants by the symbols in the header, where the first two terms of $R_{A_{+}\beta _{2},A_{+}\beta _{2}}= \frac{4}{5}d^{2}P_{0}^{2}\tau _c + \frac{3}{5}d^{2}P_{0}^{2}J(0,\omega _{A})$

**Table 3:** Macromolecular limit auto-relaxation rates for single quantum *A* spin transitions in an irreducible basis for an $AX_2$ system (‘diagonal’ transitions in Fig. 4B).

Rate	$d^2$ $P_0^2$	$d^2$ $P_0^2$	$dc_AP_0$	$dc_AP_0$	$c_A^2$	$c_A^2$	$d^2$ $P_1^2$	$d^2$ $P_2^2$	$e^2$	$ec_XP_2$	$c_X^2$ $P_1^2$	$c_X^2$ $P_2^2$
	$\tau _c$	$J(0,\omega _A)$	$\tau _c$	$J(0,\omega _A)$	$\tau _c$	$J(0,\omega _A)$	$\tau _m$	$\tau _m$	$\tau _m$	$\tau _m$	$\tau _m$	$\tau _m$
$R_{A_{+}\left| 1\right\rangle \left\langle 1\right| }$	$\frac{4}{5}$	$\frac{3}{5}$	$\frac{8}{5}$	$\frac{6}{5}$	$\frac{4}{5}$	$\frac{3}{5}$	$\frac{1}{3}$	$\frac{1}{20}$	$\frac{27}{160}$	$\frac{3}{40}$	$\frac{2}{5}$	$\frac{1}{40}$
$R_{A_{+}\left| 2\right\rangle \left\langle 2\right| }$					$\frac{4}{5}$	$\frac{3}{5}$	$\frac{1}{3}$	$\frac{1}{24}$	$\frac{9}{80}$		$\frac{8}{15}$	$\frac{1}{20}$
$R_{A_{+}\left| 3\right\rangle \left\langle 3\right| }$	$\frac{4}{5}$	$\frac{3}{5}$	$-\frac{8}{5}$	$-\frac{6}{5}$	$\frac{4}{5}$	$\frac{3}{5}$	$\frac{1}{3}$	$\frac{1}{20}$	$\frac{27}{160}$	$-\frac{3}{40}$	$\frac{2}{5}$	$\frac{1}{40}$
$R_{A_{+}\left| 4\right\rangle \left\langle 4\right| }$					$\frac{4}{5}$	$\frac{3}{5}$	1				$\frac{4}{3}$	

The results were obtained for an $AX_2$ group rotating about its symmetry axis in a diffusive manner, with isotropic global tumbling (ISO ROT). The equations include the effects of AX dipolar interactions, XX dipolar interactions, CSA interactions from nucleus A, and CSA interactions from nucleus X

**Table 4:** Macromolecular limit relaxation rates for an $AX_3$ system that is rotating about its symmetry axis in a 3-site hopping manner, with isotropic global tumbling (ISO, ROT) with$\tau _m=\frac{1}{3k}$where *k* is the hopping rate

Rate	$d^2P_0^2$	$d^2P_0^2$	$dc_AP_0$	$dc_AP_0$	$c_A^2$	$c_A^2$	$d^2P_1^2$	$d^2P_2^2$	$e^2$	$ec_XP_2$	$c_X^2P_1^2$	$c_X^2P_2^2$
	$\tau _c$	$J(0,\omega _A)$	$\tau _c$	$J(0,\omega _A)$	$\tau _c$	$J(0,\omega _A)$	$\tau _m$	$\tau _m$	$\tau _m$	$\tau _m$	$\tau _m$	$\tau _m$
$R_{A_{+}\beta _{3}}$	$\frac{9}{5}$	$\frac{27}{20}$	$-\frac{12}{5}$	$-\frac{9}{5}$	$\frac{4}{5}$	$\frac{3}{5}$	$\frac{1}{2}$	$\frac{1}{8}$	$\frac{27}{16}$	$-\frac{9}{20}$	$\frac{3}{5}$	$\frac{3}{20}$
$R_{A_{+}\alpha \beta _{2}}$	$\frac{1}{5}$	$\frac{3}{20}$	$-\frac{4}{5}$	$-\frac{3}{5}$	$\frac{4}{5}$	$\frac{3}{5}$	$\frac{29}{30}$	$\frac{29}{120}$	$\frac{99}{80}$	$-\frac{3}{20}$	$\frac{3}{5}$	$\frac{3}{20}$
$R_{A_{+}\alpha _{2}\beta }$	$\frac{1}{5}$	$\frac{3}{20}$	$\frac{4}{5}$	$\frac{3}{5}$	$\frac{4}{5}$	$\frac{3}{5}$	$\frac{29}{30}$	$\frac{29}{120}$	$\frac{99}{80}$	$\frac{3}{20}$	$\frac{3}{5}$	$\frac{3}{20}$
$R_{A_{+}\alpha _{3}}$	$\frac{9}{5}$	$\frac{27}{20}$	$\frac{12}{5}$	$\frac{9}{5}$	$\frac{4}{5}$	$\frac{3}{5}$	$\frac{1}{2}$	$\frac{1}{8}$	$\frac{27}{16}$	$\frac{9}{20}$	$\frac{3}{5}$	$\frac{3}{20}$
$R_{A_{+}\alpha \beta _{2},A_{+}\beta _{3}}$							$\frac{\sqrt{3}}{20}$	$\frac{\sqrt{3}}{80}$	$-\frac{\sqrt{243}}{80}$	$\frac{\sqrt{27}}{20}$	$-\frac{\sqrt{3}}{5}$	$-\frac{\sqrt{3}}{20}$
$R_{A_{+}\alpha _{2}\beta ,A_{+}\beta _{3}}$									$-\frac{\sqrt{243}}{20}$			
$R_{A_{+}\alpha _{2}\beta ,A_{+}\alpha \beta _{2}}$							$\frac{1}{10}$	$\frac{1}{40}$	$-\frac{27}{40}$		$-\frac{2}{5}$	$-\frac{1}{10}$

The equations include the effects of AX dipolar interactions, XX dipolar interactions, CSA interactions from nucleus A, and CSA interactions from nucleus X

**Table 5:** Macromolecular limit auto-relaxation rates for transitions in an irreducible basis for an $AX_3$ system, where only nucleus A changes spin-state. The results were obtained for an $AX_3$ group rotating about its symmetry axis in a 3-site hopping manner, with isotropic global tumbling (ISO ROT) where $\tau _m=\frac{1}{3k}$ and *k* is the inter-site hopping rate

Rate	$d^2P_0^2$	$d^2P_0^2$	$dc_AP_0$	$dc_AP_0$	$c_A^2$	$c_A^2$	$d^2P_1^2$	$d^2P_2^2$	$e^2$	$ec_XP_2$	$c_X^2P_1^2$	$c_X^2P_2^2$
	$\tau _c$	$J(0,\omega _A)$	$\tau _c$	$J(0,\omega _A)$	$\tau _c$	$J(0,\omega _A)$	$\tau _m$	$\tau _m$	$\tau _m$	$\tau _m$	$\tau _m$	$\tau _m$
$R_{A_{+}\left| 1\right\rangle \left\langle 1\right| }$	$\frac{9}{5}$	$\frac{27}{20}$	$\frac{12}{5}$	$\frac{9}{5}$	$\frac{4}{5}$	$\frac{3}{5}$	$\frac{1}{2}$	$\frac{1}{8}$	$\frac{27}{16}$	$\frac{9}{20}$	$\frac{3}{5}$	$\frac{3}{20}$
$R_{A_{+}\left| 2\right\rangle \left\langle 2\right| }$	$\frac{1}{5}$	$\frac{3}{20}$	$\frac{4}{5}$	$\frac{3}{5}$	$\frac{4}{5}$	$\frac{3}{5}$	$\frac{1}{2}$	$\frac{1}{8}$	$\frac{27}{16}$	$\frac{3}{20}$	$\frac{11}{15}$	$\frac{11}{60}$
$R_{A_{+}\left| 3\right\rangle \left\langle 3\right| }$	$\frac{1}{5}$	$\frac{3}{20}$	$-\frac{4}{5}$	$-\frac{3}{5}$	$\frac{4}{5}$	$\frac{3}{5}$	$\frac{1}{2}$	$\frac{1}{8}$	$\frac{27}{16}$	$-\frac{3}{20}$	$\frac{11}{15}$	$\frac{11}{60}$
$R_{A_{+}\left| 4\right\rangle \left\langle 4\right| }$	$\frac{9}{5}$	$\frac{27}{20}$	$-\frac{12}{5}$	$-\frac{9}{5}$	$\frac{4}{5}$	$\frac{3}{5}$	$\frac{1}{2}$	$\frac{1}{8}$	$\frac{27}{16}$	$-\frac{9}{20}$	$\frac{3}{5}$	$\frac{3}{20}$
$R_{A_{+}\left| 5\right\rangle \left\langle 5\right| }$	$\frac{1}{5}$	$\frac{3}{20}$	$\frac{4}{5}$	$\frac{3}{5}$	$\frac{4}{5}$	$\frac{3}{5}$	$\frac{31}{30}$	$\frac{31}{120}$	$\frac{27}{16}$	$\frac{3}{4}$	$\frac{6}{5}$	$\frac{3}{10}$
$R_{A_{+}\left| 6\right\rangle \left\langle 6\right| }$	$\frac{1}{5}$	$\frac{3}{20}$	$-\frac{4}{5}$	$-\frac{3}{5}$	$\frac{4}{5}$	$\frac{3}{5}$	$\frac{31}{30}$	$\frac{31}{120}$	$\frac{27}{16}$	$-\frac{3}{4}$	$\frac{6}{5}$	$\frac{3}{10}$
$R_{A_{+}\left| 7\right\rangle \left\langle 7\right| }$	$\frac{1}{5}$	$\frac{3}{20}$	$\frac{4}{5}$	$\frac{3}{5}$	$\frac{4}{5}$	$\frac{3}{5}$	$\frac{31}{30}$	$\frac{31}{120}$	$\frac{27}{16}$	$\frac{3}{4}$	$\frac{6}{5}$	$\frac{3}{10}$
$R_{A_{+}\left| 8\right\rangle \left\langle 8\right| }$	$\frac{1}{5}$	$\frac{3}{20}$	$-\frac{4}{5}$	$-\frac{3}{5}$	$\frac{4}{5}$	$\frac{3}{5}$	$\frac{31}{30}$	$\frac{31}{120}$	$\frac{27}{16}$	$-\frac{3}{4}$	$\frac{6}{5}$	$\frac{3}{10}$

The equations include the effects of AX dipolar interactions, XX dipolar interactions, CSA interactions from nucleus A, and CSA interactions from nucleus X

**Table 6:** Macromolecular limit auto-relaxation rates $AX_4$,$AX_6$,$AX_8$,$AX_{12}$and$AX_{20}$ with spins arranged as the vertices in platonic solids (tetrahedron, octahedron, cube, icosahedron and dodecahedron, respectively)

	$AX_{4}$		$AX_{6}$		$AX_{8}$		$AX_{12}$		$AX_{20}$	
No.$\beta$’s	$d^2$	$d^2$	$d^2$	$d^2$	$d^2$	$d^2$	$d^2$	$d^2$	$d^2$	$d^2$
	$\tau _c$	$J(0,\omega _A)$	$\tau _c$	$J(0,\omega _A)$	$\tau _c$	$J(0,\omega _A)$	$\tau _c$	$J(0,\omega _A)$	$\tau _c$	$J(0,\omega _A)$
20	–	–	–	–	–	–	–	–	0	0
19	–	–	–	–	–	–	–	–	$\frac{4}{5}$	$\frac{3}{5}$
18	–	–	–	–	–	–	–	–	$\frac{144}{95}$	$\frac{108}{95}$
17	–	–	–	–	–	–	–	–	$\frac{204}{95}$	$\frac{153}{95}$
16	–	–	–	–	–	–	–	–	$\frac{256}{95}$	$\frac{192}{95}$
15	–	–	–	–	–	–	–	–	$\frac{60}{19}$	$\frac{45}{19}$
14	–	–	–	–	–	–	–	–	$\frac{336}{95}$	$\frac{252}{95}$
13	–	–	–	–	–	–	–	–	$\frac{364}{95}$	$\frac{273}{95}$
12	–	–	–	–	–	–	0	0	$\frac{384}{95}$	$\frac{288}{95}$
11	–	–	–	–	–	–	$\frac{4}{5}$	$\frac{3}{5}$	$\frac{396}{95}$	$\frac{297}{95}$
10	–	–	–	–	–	–	$\frac{16}{11}$	$\frac{12}{11}$	$\frac{80}{19}$	$\frac{60}{19}$
9	–	–	–	–	–	–	$\frac{108}{55}$	$\frac{81}{55}$	$\frac{396}{95}$	$\frac{297}{95}$
8	–	–	–	–	0	0	$\frac{128}{55}$	$\frac{96}{55}$	$\frac{384}{95}$	$\frac{288}{95}$
7	–	–	–	–	$\frac{4}{5}$	$\frac{3}{5}$	$\frac{28}{11}$	$\frac{21}{11}$	$\frac{364}{95}$	$\frac{273}{95}$
6	–	–	0	0	$\frac{48}{35}$	$\frac{36}{35}$	$\frac{144}{55}$	$\frac{108}{55}$	$\frac{336}{95}$	$\frac{252}{95}$
5	–	–	$\frac{4}{5}$	$\frac{3}{5}$	$\frac{12}{7}$	$\frac{9}{7}$	$\frac{28}{11}$	$\frac{21}{11}$	$\frac{60}{19}$	$\frac{45}{19}$
4	0	0	$\frac{32}{25}$	$\frac{24}{25}$	$\frac{64}{35}$	$\frac{48}{35}$	$\frac{128}{55}$	$\frac{96}{55}$	$\frac{256}{95}$	$\frac{192}{95}$
3	$\frac{4}{5}$	$\frac{3}{5}$	$\frac{36}{25}$	$\frac{27}{25}$	$\frac{12}{7}$	$\frac{9}{7}$	$\frac{108}{55}$	$\frac{81}{55}$	$\frac{204}{95}$	$\frac{153}{95}$
2	$\frac{16}{15}$	$\frac{4}{5}$	$\frac{32}{25}$	$\frac{24}{25}$	$\frac{48}{35}$	$\frac{36}{35}$	$\frac{16}{11}$	$\frac{12}{11}$	$\frac{144}{95}$	$\frac{108}{95}$
1	$\frac{4}{5}$	$\frac{3}{5}$	$\frac{4}{5}$	$\frac{3}{5}$	$\frac{4}{5}$	$\frac{3}{5}$	$\frac{4}{5}$	$\frac{3}{5}$	$\frac{4}{5}$	$\frac{3}{5}$
0	0	0	0	0	0	0	0	0	0	0

XX dipolar coupling and X CSA contribute only high frequency terms which do not substantially contribute to the relaxation rate in the macromolecular limit. The numerical values for the coefficients for the$\tau _c$terms can be calculated using Eq. B7

## Results

RelCalc was first tested by ensuring that it can accurately reproduce results from various sources in the literature (Kay et al. 1992; Jason 2003; Bacon et al. 1963; Flemming Hansen et al. 2009; Tugarinov et al. 2007; Müller et al. 1969). To illustrate the software, we present a series of calculations that identify relaxation interference effects in scalar coupled $X_n$ and $AX_n$ spin systems with various sets of interactions and motions in the molecular frame. These are all of experimental interest. The input files to generate these results using RelCalc are provided with the software download.

### X$_2$ (long-lived states)

**Fig. 2:**
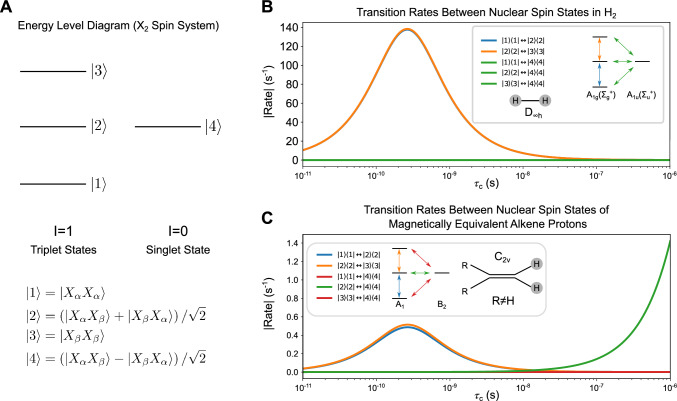
${\textbf {A}}$ The energy level diagram of an X$_{2}$ group in an external magnetic field. The irreducible wave-functions of the X spins can be arranged into a spin 1 triple ($\left| 1\right\rangle ,\left| 2\right\rangle ,\left| 3\right\rangle$) and a spin 0 singlet ($\left| 4\right\rangle$). ${\textbf {B}}$ All zero/single quantum transition rates in a diatomic hydrogen molecule. The transition rate from the singlet state ($\left| 4\right\rangle$) to any of the triplet states ($\left| 1\right\rangle ,\left| 2\right\rangle ,\left| 3\right\rangle$) is zero for the interactions considered, rendering magnetisation that can be stored in state $\left| 4\right\rangle$ extremely long-lived. The H-H bond length was taken as 0.74Å, and the symmetry labels for $D_{\infty h}$ are shown. ${\textbf {C}}$ All zero/single quantum transition rates for a pair of magnetically equivalent alkene protons. For small molecules (small $\tau _{c} \approx 10^{-10}s$), the transition rate from the singlet state ($\left| 4\right\rangle$) to any of the triplet states ($\left| 1\right\rangle ,\left| 2\right\rangle ,\left| 3\right\rangle$) is effectively zero, thereby making magnetisation placed in state $\left| 4\right\rangle$ extremely long-lived. The C-H bond length was taken as 1.09Å, a bond H-C-H bond angle of 120$^{o}$, and the CSA, $\Delta\delta _{H}$ was set to 1.0 ppm. The cross-relaxation rate between the singlet state and the $m=0$ state in the triplet becomes significant when the tumbling rate of the molecule becomes large

Two dipolar coupled degenerate spins $\frac{1}{2}$ with an axially symmetric CSA $\Delta\delta _X$ (in ppm) were arranged in the molecular frame by rotating both by a polar angle $\beta$ from the *z* axis, and rotating one spin by $\pi$ radians about the *z* axis. A third external proton was added and dipolar coupled to both nuclei leading to four interaction constants:

13 $$\begin{aligned} \begin{array}{rlccrlccrlccrl} e=\frac{\mu _{0}\hbar \gamma _X^2}{4\pi (R_{XX})^3} \quad c_X=\frac{1}{3}\gamma _XB_0 \Delta\delta _{X} \quad f = \frac{\mu _{0}\hbar \gamma _{H}\gamma _{X}}{4 \pi (R_{HX_{1}})^{3}} \quad h = \frac{\mu _{0}\hbar \gamma _{H}\gamma _{X}}{4 \pi (R_{HX_{2}})^{3}}\\ \end{array} \end{aligned}$$

where $R_{XX}$ is the distance between the two X nuclei, and $R_{HX_{1}}/R_{HX_{2}}$ are the distances between the external proton and each of the X nuclei. The other symbols are defined earlier in the text (Table 1). The irreducible representation (Fig. 2A) was evaluated, which can be summarised as $\frac{1}{2}\otimes \frac{1}{2}=1\oplus 0$ indicating two spins $\frac{1}{2}$ can be decomposed into a $J=1$ triplet, $\left| 1\right\rangle ,\left| 2\right\rangle ,\left| 3\right\rangle$ and a $J=0$ singlet, $\left| 4\right\rangle$. Each manifold carries a symmetry label that depends on the point group (Fig. 2B, C). The equilibrium operator, $\hat{X}^1_z+\hat{X}^2_z$ is equal to $\frac{1}{2}\left( \left| 1\right\rangle \left\langle 1\right| -\left| 3\right\rangle \left\langle 3\right| \right)$ and so RF pulses will inter-convert magnetisation within the $J=1$ manifold, but the singlet state cannot be created directly using pulses that uniformly excite all *X* spins. As previously noted and exploited widely, the singlet state has a remarkable auto-relaxation rate:

14 $$\begin{aligned} & R_{\left| 4\right\rangle \left\langle 4\right| }= \frac{1}{5}c_X^2P_1^2 \left( \frac{8}{3} \tau _c + 4 J\left( 0,\omega _X\right) \right) \nonumber \\ & +\frac{3}{10}\left( f-h\right) ^2\left( \tau _c+\left( J(0,\omega _H)+J(0,\omega_X+\omega _H\right) \right) \end{aligned}$$

The *XX* dipolar coupling (*e*) has vanished, leaving only CSA and external proton interactions. Moreover, if the external spin is equi-distance from the two spins of interest ($f=h$), its effects also average to zero. If the principal axis of the two axially symmetric CSA tensors point along the bond vector, $P_1=0$ ($\theta =\pi /2$), then the BWR relaxation rate is exactly zero. This is the case for para-hydrogen (Fig. 2B, green line), which has a ’long-lived’ state (Malcolm and Levitt 2012) where relaxation will come only from other weaker interactions and collisions that distort the symmetry. For comparison, the auto relaxation rate for the $m=0$ operators in the triplet state is:

15 $$\begin{aligned} & R_{\left| 2\right\rangle \left\langle 2\right| }= \frac{3}{20}\left( f-h\right) ^2\left( \tau _c + 2 J\left( 0,2\omega _X \right) \right) + \frac{3}{5} J\left( 0,\omega _X\right) e^2 \nonumber \\ & +\frac{1}{5} c_X^2\left( \frac{8}{3} \tau _c P_1^2 + 12 J\left( 0,\omega _X\right) P_0^2 + J\left( 0,\omega _X\right) P_2^2 \right) \end{aligned}$$

where the *XX* dipolar coupling term remains present and will contribute significant to relaxation. This effect using para(singlet)-hydrogen as a proton donor for hyperpolarisation NMR experiments has been exploited to great effect (Vasos et al. 2009). A related case is that of an alkene (Fig. 2C), where a similar effect is expected when two protons are (or close to being) degenerate. However, though long-lived, the singlet state’s relaxation rate will be higher than that of para-$H_2$ as the principal axis of the CSA tensors are no longer co-linear and $|P_1|>0$.

This property is not general for singlet states. Tetrahedral dipolar coupled $X_4$ spin systems contain a degenerate singlet state (E symmetry) that experiences appreciable relaxation due to internal dipolar interactions to states in adjacent T$_2$ (J=1) and A$_1$ (J=2) manifolds (Sect. 3.6). Moreover, as will be seen with $AX_2$ spin systems, introducing dipolar coupling between the A and X spins can open relaxation pathways between different *J* manifolds. While many useful properties of these spin systems can be exploited in TROSY experiments, the states will not be as ‘long-lived’.

### Quadrupolar relaxation in a DH spin system

An HD spin pair was arranged with a dipolar interaction between the two spins separated by a distance $R_{HD}$. A symmetric quadrupolar interaction was applied to the *D* spin with two components *B* and *G*, parameterised by $V_{ax}=\frac{1}{3}(V_{zz}-V_{yy})$ and $V_{ax2}=\frac{1}{3}(V_{xx}-V_{yy})$ respectively, where $V_{ii}$ are the eigenvalues of the electric field gradient tensor *V*. An anisotropic chemical shift tensor was applied to the D spin with two symmetric components $C_{ax1}$ and $C_{ax2}$ parameterised by $\Delta \delta _{ax}=\delta _{zz}-\delta _{yy}$ and $\Delta \delta _{ax2}=\delta _{xx}-\delta _{yy}$, where $\delta _{ii}$ are the eigenvalues from the second rank tensor decomposition of the chemical shift tensor, and an asymmetric component $C_{as}$ parameterised by $\Delta \delta _{as}=\sqrt{\delta _{xy}^2+\delta _{xz}^2+\delta _{yz}^2}$, derived from components of the first rank tensor decomposition. Angular dependencies of the anisotropic interactions in the molecular frame where provided, that naturally cancelled out under the effects of isotropic tumbling as described earlier. The constants used are:

16 $$\begin{aligned} \begin{array}{rlrlrl} d& =\frac{\mu _0 \hbar \gamma _H \gamma _D}{4\pi (R_{HD})^3} & B& =\frac{e V_{ax}Q}{\hbar } & G& =\frac{e V_{ax2}Q}{\hbar } \\ C_{ax}& =\frac{1}{3}\gamma _D B_0 \Delta\delta _{ax} & C_{ax2}& =\frac{1}{3}\gamma _D B_0 \Delta \delta _{2} & C_{as}& = \gamma _D B_0 \Delta \delta _{as} \\ \end{array} \end{aligned}$$

The longitudinal relaxation rate would have been naively returned with terms in $G^2+B^2+2GB P^2_0(\cos \gamma )$ where $\gamma$ is the angle between the two symmetric tensor components. As this is $90^o$, RelCalc inserted the value $P_0^2(\cos \frac{\pi }{2})=-\frac{1}{2}$ to return the following rates for the *D* spin describing longitudinal

17 $$\begin{aligned} \begin{aligned}&R_1^D=R_{D1z,D1z}= (G^2+B^2 -BG) \left( \frac{3}{40} J(0,\omega _D) + \frac{3}{10} J(0,2\omega _D) \right) \\&+(C_{ax1}+C_{ax2}^2 - C_ {ax1}C_{ax2} ) \frac{6}{5} J(0,\omega _D) +C_{as} \frac{2}{3}J^1(0,\omega _D) \\&+d^2\left( \frac{1}{10} J(0,\omega _H-\omega _D) + \frac{3}{10}J(0,\omega _D) + \frac{3}{5}J(0,\omega _H+\omega _D)\right) \end{aligned} \end{aligned}$$

and transverse relaxation rates

18 $$\begin{aligned} \begin{aligned}&R_2^D = R_{D1x,D1x}= (G^2+B^2 -BG) \left( \frac{9}{80}\tau _c \right. \\&\left. + \frac{3}{16} J(0,\omega _D) + \frac{3}{40} J(0,2\omega _D) \right) \\&+(C_{ax1}+C_{ax2}^2 - C_ {ax1}C_{ax2} ) \left( \frac{4}{5} \tau _C + \frac{3}{5} J(0,\omega _D) \right) +C_{as} \frac{1}{3}J^1(0,\omega _D) \\&+ d^2\left( \frac{1}{5}\tau _c + \frac{1}{20} J(0,\omega _H-\omega _D)\right. \\&\left. + \frac{3}{20}J(0,\omega _D) +\frac{3}{10}J(0,\omega _H) + \frac{3}{10}J(0,\omega _H+\omega _D)\right) \end{aligned} \end{aligned}$$

where $J^1$ emphasises that the rank 1 tensors should be used for the time-scale in this reduced spectral density. In the extreme narrowing limit ($\omega>>\tau _c$ and so $J(0,\omega )=\tau _c$) these reduce to

19 $$\begin{aligned} \begin{aligned} R_1^D =&\left( \frac{3}{8}(G^2+B^2 -BG) + \frac{6}{5} (C_{ax1}+C_{ax2}^2 \right. \\&\left. - C_ {ax1}C_{ax2} ) + 2 C_{as} + d^2\right) \tau _c \\ R_2^D =&\left( \frac{3}{8}(G^2+B^2 -BG) + \frac{7}{5} (C_{ax1}+C_{ax2}^2 \right. \\&\left. - C_ {ax1}C_{ax2} ) + C_{as} + d^2\right) \tau _c \end{aligned} \end{aligned}$$

Because of the anisotropic chemical shift, $R_2^D$ does not converge on $R_1^D$ in the rapid tumbling limit. Neglecting contributions from CSA asymmetry, then $R_2^D>R_1^D$ for all values of $\omega$.

There are several methods to parameterise quadrupolar tensor relaxation rates. Here, a constant of the following form arises, that describes the overall effect of the tensor $(G^2+B^2 -BG)$. If we set $B=0$ we recover $G^2$, the result expected for an axially symmetric tensor. Similarly, if $B=G$, then $V_{zz} = V_{xx}$ and the result reduces to $B^2$, consistent again with a single axially symmetric tensor. As both *G* and *B* are differences in tensorial components, when the tensor becomes symmetric both *B* and *G* tend to zero and the quadrupolar interaction stops causing relaxation. The combined effect of two components are not additive, and correlations between the two lead to partial cancellation of their effects. Noting the definitions (Appendix A1) $G=\frac{1}{3}(V_{zz}-V_{yy})$ and $B=\frac{1}{3}(V_{xx}-V_{yy})$, and either defining the root mean squared of the tensor $|V|=\sqrt{\frac{1}{3}\left( V_{xx}^2+V_{yy}^2+V_{zz}^2\right) }$ or an average of the three differences, $|V|=\sqrt{\frac{1}{9}\left( (V_{xx}-V_{yy})^2+(V_{zz}-V_{yy})^2+(V_{zz}-V_{xx})^2\right) }$ and noting that the tensor is traceless $V_{zz}=-V_{yy}-V_{xx}$, then $B^2+G^2-BG = \frac{|V|^2}{2}$.

The definitions used for the parameters by RelCalc differ to those often used when describing quadrupolar relaxation. These are $V=2V_{zz}-V_{xx}-V_{yy}=3V_{zz}$ and an asymmetry value $\eta V=\frac{1}{2}\left( V_{xx}-V_{yy}\right)$. We can relate the two definitions via $V^{ax}= V(1-\frac{1}{3} \eta )$ and $V^{ax2} = V \eta \frac{2}{3}$ (Appendix A1). With these substitutions,

20 $$\begin{aligned} G^2+B^2-BG = 3V_{zz} ( 1 + \frac{1}{3}\eta ^2), \end{aligned}$$

which is the commonly encountered form. As $V_{zz}$ is the leading term, this parameterisation partially masks the observation that relaxation depends specifically on differences between tensoral components. The same argument can be used to re-parameterise the relaxation contributions $C_{ax1}^2+C_{ax2}^2-C_{ax1}C_{ax2}$ from the CSA tensor if required. Both approaches agree that the effects of the two tensoral components are indistinguishable from relaxation effects alone when the molecule isotropically tumbling.

### *AX*

**Fig. 3:**
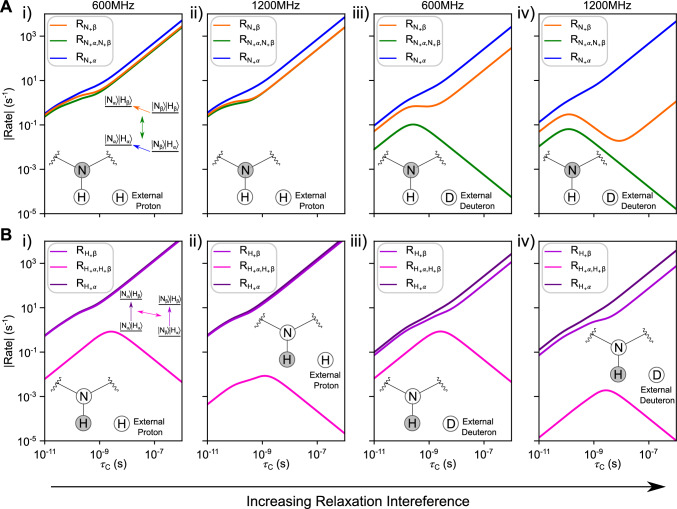
**A **Three $\hat{N}_+$ relaxation rates in a $^{15}$NH group in the presence of external protons (Ai,Aii) and external deuterons (Aiii,Aiv) in a spectrometer operating at a Larmor frequency of either 600 MHz or 1.2 GHz. External protons cross relaxing with the NH proton greatly increase the cross-relaxation rate $R_{N_{+}\alpha ,N_{+}\beta }$, which leads to an equalising of the two diagonal relaxation rates and reduction of the TROSY effect. Cross relaxation effects caused by an external proton are significant, where to an external deuteron they are almost completely eliminated, emphasising why deuteration is a key part of the TROSY method and it is desirable to perform the experiments at 1.2 GHz over a 600 MHz system (orange lines, Aiv). **B** Equivalent plots as in **A**, but for the $\hat{H}_{+}$ relaxation rates in a $^{15}$NH group. As for $^{15}N$, the difference in relaxation rates is promoted by deuteration. Rates were calculated taking R_NH_=1 Å, $\Delta\delta _{N} = -150$ ppm (Pandey 2012), $\Delta\delta _{H} = 10$ ppm, $R_{N-X_{\text {ext}}}$= 2 Å, $R_{H-X_{\text {ext}}}$ = 1.5 Å

Following uniform $^{15}N$ enrichment, the $^{15}H^1N$ pair on the protein backbone is a commonly encountered spin $\frac{1}{2}$ scalar coupled AX spin system in biomolecular NMR. When transverse *N* magnetisation is created in experiments, we need to consider evolution of the following two operators $\hat{\rho }_{A_{+}\alpha } = \hat{A}_{+}\hat{X}_{\alpha }$ and $\hat{\rho }_{A_{+}\beta } = \hat{A}_{+}\hat{X}_{\beta }$. Following Eq. 1, these states will evolve in the rotating frame, on resonance, according to:

21 $$\begin{aligned} \frac{d}{dt} \begin{pmatrix} \rho _{A_{+}\alpha } \\ \rho _{A_{+}\beta } \end{pmatrix} = - \begin{pmatrix} R_+-i\frac{J}{2} & R_{A_{+}\alpha ,A_{+}\beta } \\ R_{A_{+}\alpha ,A_{+}\beta } & R_- +i\frac{J}{2} \end{pmatrix} \begin{pmatrix} \rho _{A_{+}\alpha } \\ \rho _{A_{+}\beta } \end{pmatrix} \end{aligned}$$

where $R_\pm$ corresponds to the auto-relaxation of the $A_{+}\alpha$ and $A_{+}\beta$ states respectively, and the off-diagonal relaxation rates describe cross relaxation. The three relaxation rates were determined using RelCalc including the inter-nuclear dipolar interaction (*d*), co-linear to both axially symmetric CSA interactions from A ($c_{A}$) and X ($c_{X}$) and dipolar coupling from two external spins Y to X (f) and to A (g), where *Y* can either be a proton $f_H/g_H$ or a deuteron $f_D/g_D$:

22 $$\begin{aligned} \begin{aligned} d=&\frac{\mu _{0}\hbar \gamma _A\gamma _X}{4\pi (R_{AX})^3} \quad f_Y=\frac{\mu _{0}\hbar \gamma _X\gamma _Y}{4\pi (R_{XY})^3} \quad g_Y=\frac{\mu _{0}\hbar \gamma _A\gamma _Y}{4\pi (R_{AY})^3} \\ c_X&=\frac{1}{3}\gamma _XB_0 \Delta\delta _{X} \quad c_A=\frac{1}{3}\gamma _AB_0 \Delta\delta _{A} \end{aligned} \end{aligned}$$

Taking the macromolecular limit for simplicity, the diagonal rates take the form:

23 $$\begin{aligned} \begin{aligned} R_{\pm }=&\left( \left( d \pm 2c_A\right) ^2 +g_H^2 +\frac{8}{3}g_D^2\right) \left( \frac{1}{5} \tau _c +\frac{3}{20} J\left( \omega _A\right) \right) \\&+ \frac{1}{20}f_H^2 \tau _c + \frac{4}{5} g_D^2J\left( \omega _D\right) \end{aligned} \end{aligned}$$

Relaxation interference is apparent, with the $R_+$ resonance experiencing constructive association of CSA and AX dipolar interactions and $R_-$ experiencing destructive interference. The effects of the external proton/deuteron is to equalise the rates and ‘dilute’ the effects of the interference. As $\frac{(f_D/g_D)^2}{(f_H/g_H)^2}=\frac{\gamma _D^2}{\gamma _H^2}\approx 0.06$, having external deuterons rather than external protons enhances the differences between the two rates and so enhances TROSY. In order to observe a TROSY effect (relaxation interference), it is insufficient for the two relaxation rates two be different, we also need to have negligible cross relaxation between the spins, which will act to effectively equalise the two effective relaxation rates of the two resonances. In the macromolecular limit this rate is

24 $$\begin{aligned} \begin{array}{rl} R_{A_+\alpha ,A_+\beta }= - \frac{1}{20} f_H^2 \tau _c\\ \end{array}\end{aligned}$$

which depends only on the dipolar interaction between the X and external protons. This process is driven by ’flip-flop’ transitions caused by the zero quantum dipolar operators (eg $\hat{I}_+\hat{S}_-$, Table 9) which contributes a zero frequency term giving a linear dependence on $\tau _c$ in this limit. When the external spin is a deuteron, the corresponding contribution will depend on spectral density at a frequency of $\omega _X-\omega _D$, which will be negligible in the macromolecular limit for X=H. Deuterating the protein except for the NH protons being observed greatly reduces the cross relaxation rate, and so is a vital part of the *NH* TROSY (Pervushin et al. 1997) method (Fig. 3).

The three relaxation rates can be followed with correlation time for a $^{15}$NH system in the presence of external protons (Fig. 3Ai, Aii) and external deuterons (Fig. 3Aiii, Aiv) on spectrometers operating at proton Larmor frequency of 600 MHz and 1.2 GHz. In the presence of an external proton, the cross-relaxation rate $R_{A_{+}\alpha ,A_{+}\beta }$ (Fig. 3 A, green) is large, with the diagonal relaxation rates being dominated by the external proton and are almost identical. This completely suppresses the TROSY effect. By contrast, and as expected, deuterons barely affect the cross-relaxation rate and preserve the TROSY effects (Pervushin et al. 1997). At 1.2 GHz, the difference between the two diagonal relaxation rates is very significant, making a compelling case for studies of high molecular weight, deuterated complexes, enriched with $^{15}$N at 1.2 GHz.

In addition, Fig. 3Bi-iv contains the equivalent plots of the relaxation rates for the single quantum proton operators, where $H_{+}\alpha$/$H_{+}\beta$ represent the $H_{+}N_{\alpha }$/$H_{+}N_{\beta }$ operators respectively. While deuteration enables a TROSY effect for these coherences, the enhancement is marginal when compared to the benefits obtained in the $^{15}$N dimension.

### $AX_2$

**Fig. 4:**
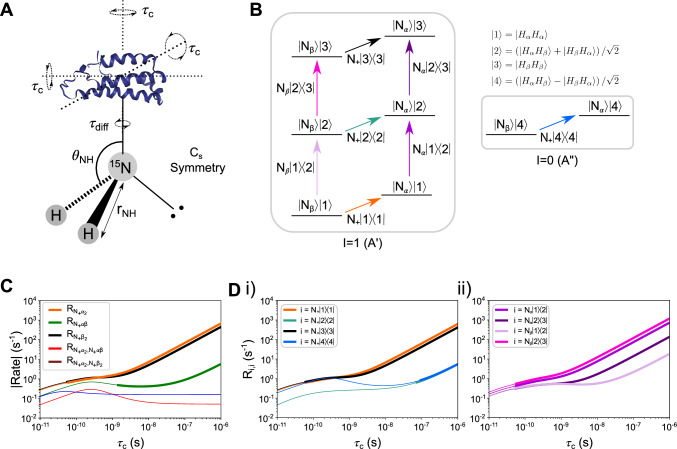
$\textbf{A}$ Diffusive motion of an $^{15}$NH$_{2}$ group about a symmetry axis with a time scale $\tau _{\text {diff}}$ in a protein that is tumbling isotropically with a time scale $\tau _{c}$. $\textbf{B}$ The energy level diagram of a $^{15}$NH$_{2}$ group with C$_{s}$ symmetry in an external magnetic field. The eigenstates of the X spins are given by $\left| 1\right\rangle -\left| 4\right\rangle$, with symmetries of A’ for the triplet states and A”for the $J=0$ singlet state. Each diagonal arrow represents single quantum transitions available to $^{15}$N, and vertical arrows represent single quantum transitions available to $^{1}$H when $^{15}$N is locked in the $\alpha$ or $\beta$ state. All transitions (arrows) can be associated with a specific Liouville operator, e.g. $\hat{N}_+\left| 2\right\rangle \left\langle 2\right|$. $\textbf{C}$ Comparison of single quantum nitrogen relaxation rates of a $^{15}$NH$_2$ group that is rotating about its symmetry axis in a diffusive manner and undergoing isotropic global motion. The plots were produced using the exact equations generated from RelCalc, and the bold regions indicate regions where the macromolecular result is within 20% of the exact result. Note that the macromolecular relaxation rates are not plotted. The $R_{N_{+}\alpha \beta }$ coherences show notably slow relaxation. $\textbf{D}$ Plots of the exact auto-relaxation rates for all diagonal (i) and vertical (ii) transitions shown in A, calculated for a $^{15}$NH$_{2}$. The thicker lines indicate regions where the macromolecular rates are within 20% of the exact rates. The spectrometer frequency was taken to be 600 MHz, $\theta _{NH}$ = $109.5^o$, $R_{NH}$ = 1Å, $\Delta\delta _N$ = 20 ppm, $\Delta\delta _H$ = 10 ppm, $\tau _{\text {diff}}$ = 25 ps

Placing the *A* spin at the origin, there are two chemically reasonably arrangements might be expected for an AX$_{2}$ group of spins $\frac{1}{2}$. If the three atoms form a plane that includes the *z* axis we have C$_{2v}$ symmetry and $R_{XX}=2R_{AX}\textrm{cos}\left( \beta \right)$ where $\beta$ is the angle between the AX bond and the positive *z* axis (eg an alkene CH$_{2}$). For biomolecular NMR applications, an alternative arrangement owing to the presence of a lone pair, we have an approximately tetrahedral arrangement and $\sqrt{3}R_{AX}\textrm{sin}\left( \beta \right)$ giving C$_s$ symmetry (eg a $^{15}$NH$_{2}$ amine). In both cases we expect free rotation about the *z* axis (Fig. 4A). Considering AX and XX dipolar interactions, together with axially symmetric CSA interactions aligned the bonds for the *X* spin and the *z* axis for the *A* spin, the various relaxation rates require the following constants:

25 $$\begin{aligned} \begin{aligned} d=&\frac{\mu _{0}\hbar \gamma _A\gamma _X}{4\pi (R_{AX})^3} \quad e=\frac{\mu _{0}\hbar \gamma _X^2}{4\pi (R_{XX})^3} \quad f_Y=\frac{\mu _{0}\hbar \gamma _X\gamma _Y}{4\pi (R_{XY})^3} \quad g_Y=\frac{\mu _{0}\hbar \gamma _A \gamma _Y}{4\pi (R_{AY})^3}\\&c_X=\frac{1}{3}\gamma _XB_0 \Delta\delta _{X} \quad c_A=\frac{1}{3}\gamma _AB_0 \Delta\delta _{A} \end{aligned} \end{aligned}$$

The two degenerate *X* spins can be described in the irreducible basis (Fig. 4B), having the same basis functions and operators as for $X_2$ following the decomposition $\frac{1}{2}\otimes \frac{1}{2}=1\oplus 0$. Considering the evolution of single quantum *A* magnetisation, we need 3 spin Zeeman basis operators, $\rho _{A_{+}\alpha _{2}}= \hat{A}_+\hat{X}_\alpha ^1 \hat{X}_\alpha ^2$, $\rho _{A_{+}\alpha \beta }= \frac{1}{\sqrt{2}}\hat{A}_+\left( \hat{X}_\alpha ^1 \hat{X}_\beta ^2 + \hat{X}_\beta ^1 \hat{X}_\alpha ^2 \right)$ and $\rho _{A_{+}\beta _{2}} = \hat{A}_+\hat{X}_\beta ^1 \hat{X}_\beta ^2$. The three Zeeman operators will evolve in the rotating frame on resonance according to

26 $$\begin{aligned} & \frac{d}{dt} \left( \begin{array}{c} \rho _{A_{+}\alpha _{2}} \\ \rho _{A_{+}\alpha \beta } \\ \rho _{A_{+}\beta _{2}} \\ \end{array} \right) =-\left( \begin{array}{cccc} R_{+1 } -iJ & R_{A_{+}\alpha _{2}, A_{+}\alpha \beta } & R_{A_{+}\alpha _{2},A_{+}\beta _{2}} \\ R_{A_{+}\alpha \beta , A_{+}\alpha _{2}} & R_{0 } & R_{A_{+}\alpha \beta ,A_{+}\beta _{2}} \\ R_{A_{+}\beta _{2}, A_{+}\alpha _{2}} & R_{A_{+}\beta _{2}, A_{+}\alpha \beta } & R_{-1 }+iJ \\ \end{array} \right) \nonumber \\ & \left( \begin{array}{c} \rho _{A_{+}\alpha _{2}} \\ \rho _{A_{+}\alpha \beta } \\ \rho _{A_{+}\beta _{2}} \\ \end{array} \right) \end{aligned}$$

The diagonal relaxation rates are auto-relaxation, and the off-diagonal rates represent cross-relaxation involving either one or two ’spin flips’. When the macromolecular limit is applied, we obtain the summarised relaxation rates (Table 2, automatically generated by RelCalc). To identify relaxation interference, we can index the diagonal relaxation rates $R_{A_{+}\alpha _{2}}$, $R_{A_{+}\beta _{2}}$ and $R_{A_{+}\alpha \beta }$ by their projected angular momentum ($+1,-1,0$). The relaxation rates when including the effects of an external proton and deuteron are:

27 $$\begin{aligned} \begin{aligned} R_{\pm }&= \frac{1}{5}\left( dP_0 \pm c_A\right) ^2 \left( 4 \tau _c +3 J\left( 0,\omega _A\right) \right) \\&+ \frac{1}{5}\tau _m\left( \frac{1}{32}\left( 3e \pm 2c_XP_2\right) ^2 + 2 c_X^2P_1^2 +\frac{d^2}{4}\left( 6 P_1^2 + P_2^2 \right) + \frac{9}{16}e^2 \right) \\&+ R_{\textrm{ext}}\\ R_0&= \frac{1}{5} c_A^2 \left( 4 \tau _c +3 J\left( 0,\omega _A\right) \right) \\&+ \frac{1}{5}\tau _m\left( c_X^2\left( 2 P_1^2 + \frac{1}{40} P_2^2\right) +\frac{d^2}{24}\left( \frac{9}{4}P_2^2 + 92 P_1^2 \right) +\frac{9}{32} e^2 \right) \\&+ R_{\textrm{ext}} \end{aligned}\end{aligned}$$

where the contribution to relaxation because of an external proton and an external deuteron is approximately

28 $$\begin{aligned} \begin{array}{rl} R_{\textrm{ext}}&=\left( g_H^2 +\frac{8}{3} g_D^2\right) \left( \frac{1}{5} \tau _c +\frac{3}{20} J\left( \omega _A\right) \right) + \frac{1}{10} \tau _cf^2_{H} \end{array}\end{aligned}$$

As $|dP_0|> |c_A|$ at 600 MHz, in the macromolecular limit, the relaxation rate for $R_{0}$ will be far lower than either rate $R_{\pm 1}$ and so the central resonance is optimal for TROSY. Relaxation rates were simulated for a $^{15}$NH$_{2}$ inside an isotropically tumbling protein (Fig. 4C), where rotation about the symmetry axis is assumed to be diffusive. This plot highlights the strong relaxation interference. The effect of external protons however is substantial, effectively dominating the relaxation rates via the $\frac{1}{10}f_{H}^2\tau _c$ term. To maximise the difference in relaxation rates, there is a need to deuterate the protein and so effectively remove this term.

As for the *AX* spin system, it is insufficient to have a difference in relaxation rates, the cross relaxation rates must also be small for there to be useful TROSY effects. In the macromolecular limit, all cross relaxation rates are zero when there is an external deuteron. When there is an external proton, all transitions involving $\Delta m=1$ ($R_{A_+\alpha \beta ,A_+\alpha _{2}}=R_{A_+\alpha \beta ,A_+\alpha _{2}}$) caused by ’flip-flop’ transitions between an X proton and the external are approximately equal to $-\frac{1}{\sqrt{200}} \tau _c f_{H}^2$.

Relaxation rates were simulated for $^{15}NH_2$ including diffusive motion about the symmetry axis in the irreducible basis (macromolecular limit expressions Table 3, Fig. 4D). Both $m_j=0$ states in the singlet and triplet states have favourable relaxation properties in carbon that become indistinguishable in the macromolecular limit.

Analysis of the single quantum proton transitions reveals both fast and slow relaxation rates where the $N_\alpha \left| 2\right\rangle \left\langle 3\right|$ and $N_\beta \left| 1\right\rangle \left\langle 2\right|$ states lead to significantly lower relaxation than the other two transitions. It would be desirable to keep these from mixing in a TROSY based experiment (Jason 2003) by avoiding $90^o$ pulses on proton. Significant deviations were observed between rates calculated in macromolecular limit (thick lines) and the exact expressions for correlation times that cover proteins ($\tau _c$ 1–100 ns), suggesting that while macromolecular limit relaxation rates provide physical insight, they should be treated with care when analysing data.

### $AX_3$

**Fig. 5:**
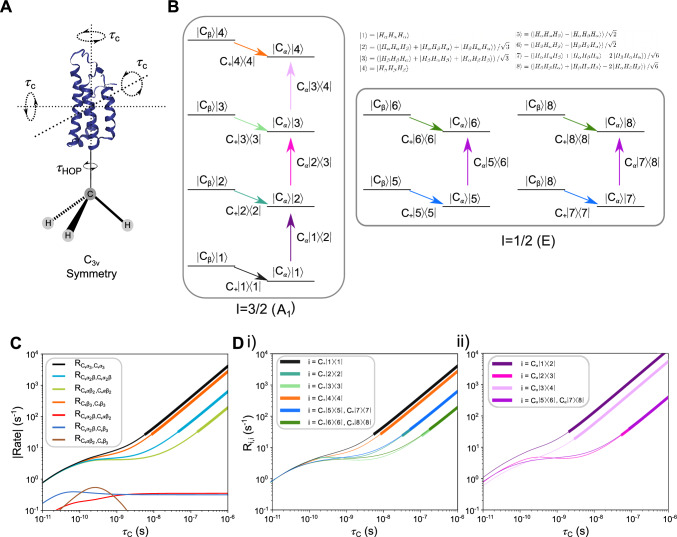
$\textbf{A}$ Geometry of a $^{13}CH_3$ group undergoing 3-site hopping motion about a symmetry axis in a protein molecule that is isotropically tumbling with time-scale $\tau _c$. $\textbf{B}$ The energy level diagram of a $^{13}$CH$_{3}$ group linked to the irreducible representation. The irreducible wave-functions $\left| 1\right\rangle -\left| 8\right\rangle$ are related to the single spin wave-functions. The arrows and stated coherences correspond to single quantum carbon (horizontal) and single quantum hydrogen (vertical) arrows. $\textbf{C}$ A comparison of the single quantum carbon Zeeman relaxation rates of an isolated $^{13}CH_3$ group that is undergoing ISO-ROT motion as depicted in A. The set of cross-relaxation rates were below $10^{0}$ s$^{-1}$ for all values of $\tau _c$. When the macromolecular results are within 20% of the exact results, the value is indicated by a thick line. The rates are in strong agreement only when $\tau _c>$ 100 ns. $\textbf{D}$ Auto-relaxation rates for single quantum carbon (i) and proton (ii) transitions in terms of the irreducible basis. The thicker lines indicate regions where the macromolecular limit rates are within 20% of the exact rates. All relaxation rates were simulated at a field strength corresponding to a proton Larmor frequency of 600 MHz and assuming the $^{13}$CH$_{3}$ group is rotating in a 3-site hopping manner, with isotropic global tumbling, $\beta _{CH}$ = 111.6$^o$, $R_{CH}$ = 1.09Å, $\Delta\delta _C$ = 20 ppm, $\Delta\delta _H$ = 1 ppm, $\tau _{HOP}=(1/3k)$ = 50 ps (Flemming Hansen et al. 2009) (note $\tau _{HOP}=3\tau _F$ where $\tau _F$ is the definition in Flemming Hansen et al. (2009))

An AX$_{3}$ group was arranged with three *AX* bonds at an angle $\beta$ from the positive *z* axis, distributed equally about the azimuthal plane conforming to $C_{3v}$ symmetry such that the inter-proton distance is given by: $R_{XX}=\sqrt{3}R_{AX}\textrm{sin}\beta$. AX and XX dipolar, and axially symmetric X and A CSA interactions were included with the X CSA parallel with the AX bonds and the A CSA parallel with the *z* axis. The relaxation rates within the system were parametrised in terms of the same constants as for AX$_{2}$ (Eq. 25). The four single quantum Zeeman basis operators are $\rho _{A_{+}\alpha _{3}}= \hat{A}_+^1\hat{X}_\alpha ^2 \hat{X}_\alpha ^3 \hat{X}_\alpha ^4$, $\rho _{A_{+}\alpha _{2}\beta }= \frac{1}{\sqrt{3}}\hat{A}_+^1\left( \hat{X}_\alpha ^2 \hat{X}_\alpha ^3 \hat{X}_\beta ^4+\hat{X}_\alpha ^2 \hat{X}_\beta ^3 \hat{X}_\alpha ^4+\hat{X}_\beta ^2 \hat{X}_\alpha ^3 \hat{X}_\alpha ^4\right)$, $\rho _{A_{+}\alpha \beta _{2}} = \frac{1}{\sqrt{3}}\hat{A}_+^1\left( \hat{X}_\alpha ^2 \hat{X}_\beta ^3 \hat{X}_\beta ^4+\hat{X}_\beta ^2 \hat{X}_\alpha ^3 \hat{X}_\beta ^4+\hat{X}_\beta ^2 \hat{X}_\beta ^3 \hat{X}_\alpha ^4\right)$ and $\rho _{A_{+}\beta _{3}} = \hat{A}_+^1\hat{X}_\beta ^2 \hat{X}_\beta ^3 \hat{X}_\beta ^4$ which will evolve in the rotating frame on resonance according to

29 $$\begin{aligned} & \frac{d}{dt} \left( \begin{array}{c} \rho _{A_{+}\alpha _{3}} \\ \rho _{A_{+}\alpha _{2}\beta } \\ \rho _{A_{+}\alpha \beta _{2}} \\ \rho _{A_{+}\beta _{3}} \\ \end{array} \right) \nonumber \\ & = - \left( \begin{array}{cccc} R_{A_{+}\alpha _{3}}-i\frac{3J}{2} & R_{A_{+}\alpha _{3}, A_{+}\alpha _{2}\beta } & R_{A_{+}\alpha _{3},A_{+}\alpha \beta _{2}} & 0 \\ R_{A_{+}\alpha _{2}\beta , A_{+}\alpha _{3}} & R_{A_{+}\alpha _{2}\beta }-i\frac{J}{2} & R_{A_{+}\alpha _{2}\beta ,A_{+}\alpha \beta _{2}} & R_{A_{+}\alpha _{2}\beta , A_{+}\beta _{3}} \\ R_{A_{+}\alpha \beta _{2}, A_{+}\alpha _{3}} & R_{A_{+}\alpha \beta _{2}, A_{+}\alpha _{2}\beta } & R_{A_{+}\alpha \beta _{2}}+i\frac{J}{2} & R_{A_{+}\alpha \beta _{2},A_{+}\beta _{3}} \\ 0 & R_{A_{+}\beta _{3}, A_{+}\alpha _{2}\beta } & R_{A_{+}\beta _{3}, A_{+}\alpha \beta _{2}} & R_{A_{+}\beta _{3}}+i\frac{3J}{2} \\ \end{array} \right) \nonumber \\ & \left( \begin{array}{c} \rho _{A_{+}\alpha _{3}} \\ \rho _{A_{+}\alpha _{2}\beta } \\ \rho _{A_{+}\alpha \beta _{2}} \\ \rho _{A_{+}\beta _{3}} \\ \end{array} \right) \end{aligned}$$

where the diagonal rates are auto-relaxation rates, the off-diagonal rates represent cross-relaxation caused by either one or two spin flips, and J is the scalar coupling constant. The zeroes in the matrix are caused by the inability to have a triple spin-flip from $\rho _{A_{+}\alpha _{3}}$ to $\rho _{A_{+}\beta _{3}}$ using the two spin operators available for the interactions in $\mathcal {H^\textrm{1}}$. The degenerate spins span an irreducible basis that spans 3 manifolds according to $\frac{1}{2}\otimes \frac{1}{2}\otimes \frac{1}{2}=\frac{3}{2}\oplus \frac{1}{2}\oplus \frac{1}{2}$.

Relaxation rates for isotropic global tumbling with rapid rotation about the 3-fold axis in the macromolecular limit are given in the Zeeman (Table 4) and irreducible (Table 5) bases. Setting the X CSA value to zero in the Zeeman basis recovers results presented previously (Flemming Hansen et al. 2009) and neglecting tumbling about the methyl axis allows us to recover rates described by Jason (2003). Relaxation interference effects between the XX dipolar interaction and the X CSA can be seen (Table 4), though in the case of $CH_3$ groups, the value of the X CSA will be small.

The quantitative picture between the single quantum Zeeman basis and the irreducible basis (Fig. 5C, Di) is similar when analysing relaxation rates associated with a $^{13}$C transition, where having a lower $m_j$ for the attached protons leads to slower relaxation as observed previously (Jason 2003), with carbon CSA-dipolar TROSY favouring one specific term (Flemming Hansen et al. 2009). The proton relaxation rates fall into ‘fast’ (from or to an $|m_j|=\frac{3}{2}$ state) and ‘slow’ (between $|m_j|=\frac{1}{2}$ states) categories as previously observed (Jason 2003) (Fig. 5Dii), where for TROSY it is desirable to avoid $^1$H pulses that cause these states to mix. Relaxation rates for various coherences are compared to those in the macromolecular limit (Fig. 5C), revealing surprisingly poor agreement even for reasonably small proteins ($\tau _c$ 1–100 ns).

**Fig. 6:**
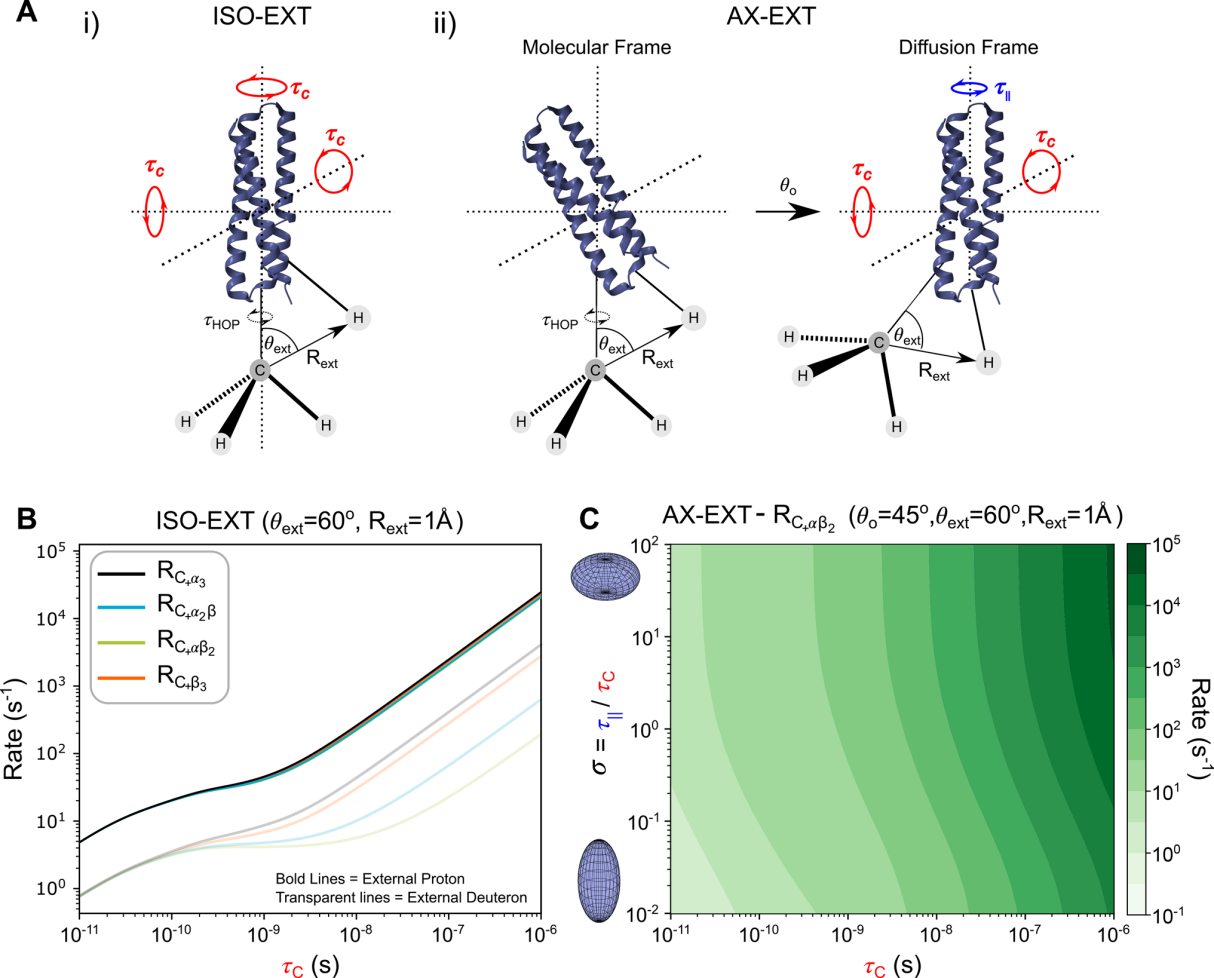
$\textbf{A}$ The geometry of a methyl group and external proton/deuteron within a protein undergoing both isotropic tumbling characterised by $\tau _c$ (i) and axially symmetric tumbling (ii), where $\tau _{\parallel }$ and $\tau _{c}$ are the rotational correlation times of the unique and degenerate axes, respectively. $\textbf{B}$ The single quantum carbon relaxation rates in a $^{13}$CH$_{3}$ group using a Zeeman basis, undergoing isotropic global tumbling. The transparent/solid lines show the relaxation rates in the presence of an external deuteron/proton, respectively. The external proton dominates relaxation and provides a mechanism for the four coherences to cross-relax, equalising the relaxation rates and suppressing the methyl-TROSY effect. $\textbf{C}$ The $R_{C_{+}\alpha \beta _2}$ relaxation rate in a $^{13}$CH$_{3}$ group undergoing axially symmetric global tumbling and interacting with an external proton. The relaxation rate is shown as a function of $\tau _c$ and $\sigma =\frac{\tau _\parallel }{\tau _c}$. Relaxation follows the fastest global tumbling time for oblate tumbling ($\sigma>1$), so the rates are largely independent of $\sigma$ for a fixed $\tau _c$ value. For prolate tumbling, $\sigma <1$ and relaxation rates track closely with $\sigma$, following the fastest tumbling rate ($\tau _\parallel$ in this regime). B and C were simulated at a field strength corresponding to a proton Larmor frequency of 600 MHz and assuming the $^{13}$CH$_{3}$ group is rotating in a 3-site hopping manner. The parameters used to simulate the curves were: $\theta _{CH}$ = 111.6$^o$, $R_{CH}$ = 1.09Å, $R_{HH}$ = 1.76Å, $\Delta\delta _C$ = 20.0 ppm, $\Delta\delta _H$ = 1.0 ppm. Images of the prolate and oblate spheroids in C were adapted from https://commons.wikimedia.org/wiki/File:Spheroids.svg (Creative Commons Attribution-Share Alike 4.0 International).

In the absence of external protons, cross-relaxation rates are uniformly low (Fig. 5C) revealing the advantages of the methyl TROSY method. When external protons are introduced, this ceases to be the case (Fig. 6B), as cross-relaxation between the individual spin states becomes efficient the relaxation rates of the various states are averaged. The transitions are primarily driven by ’flip flop’ transitions which rely on the external proton evolving at the same frequency as the methyl protons. The same transitions are significantly slower when the external protons are replaced by deuterons, rationalising the importance of deuteration in the methyl TROSY experiment (Jason 2003).

To illustrate the effects of axially symmetric rotation, the relaxation rate of the ‘TROSY’ coherence $R_{C_{+}\alpha \beta _2}$ was followed as a function of $\tau _c$ and the shape factor, $\sigma$ (Fig. 6C) where the methyl axis is co-linear with the unique diffusion axis. Relaxation is dominated by the fastest global tumbling rate in all cases. When the molecule is oblate, the fastest rotation is about the perpendicular axis, with a rotational correlation time of $\tau _c$, so relaxation rates are independent of $\sigma$ for a fixed value of $\tau _c$. By contrast, when the molecular is prolate, the fastest rotation is about the unique axis, with a correlation time of $\tau _\parallel$, and so the rates strongly depend on $\sigma$ when $\tau _c$ is held constant.

### Application to the platonic solids

**Fig. 7:**
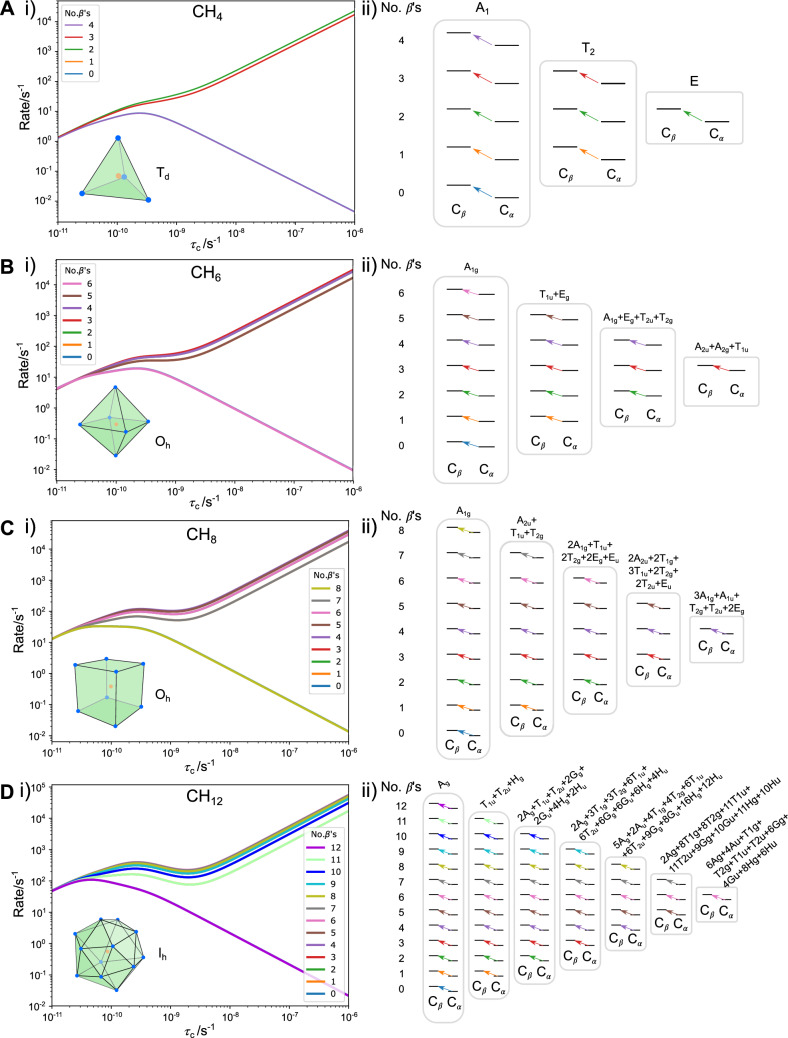
Variation of the single quantum carbon relaxation rates in the Zeeman basis (i), and the corresponding irreducible representations (ii) for $CH_n$ symmetric clusters $n=4,6,8,12$ arranged with spins in the vertices of the platonic solids (**A–D**). The plots were created including all CH and HH dipolar and H CSA interactions. The nearest C-H distance was 1.09Å and $\Delta\delta _{H} =$ 1.0 ppm. Rates are indexed by the number of hydrogen nuclei that are in the $\beta$ state for the relevant state: for CH$_{4}$, the operator $C_{+}\alpha \alpha \alpha \beta$ (including all permutations) will have an index of No.$\beta 's=1$. The relaxation rates of the high magnitude angular momentum states (No.$\beta 's=0,N$) have complete cancellation of dipolar coupling and so have remarkably slow relaxation times in the macromolecular limit

**Fig. 8:**
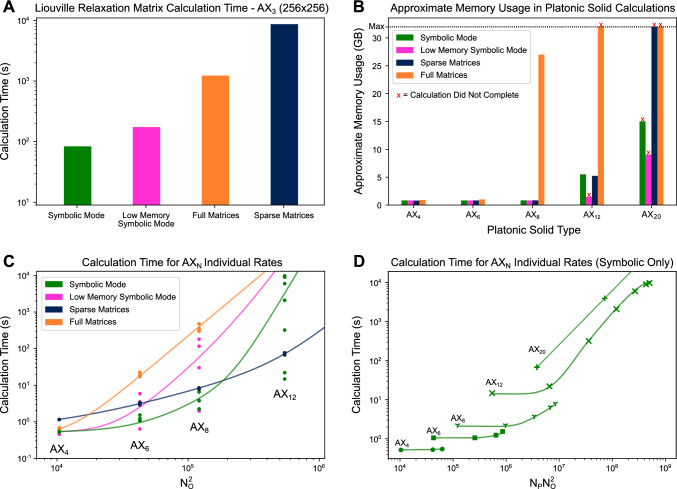
$\textbf{A}$ A comparison of the time taken to determine symbolic expressions for all 256x256 relaxation rates for a complete Cartesian Liouville basis suitable for simulating $^{13}$CH$_{3}$ groups. AX and XX dipolar interactions are included, as are A and X CSAs, with both A and X dipolar coupled to an external proton. Following calculation of the symbolic relaxation rates, recalculation of the rates for new parameters (following saving the state of the program) is rapid, taking 1.093 seconds. The calculation time for the symbolic mode is one order of magnitude faster than when using full matrices, which is one order of magnitude faster than using sparse matrices. The exact calculation times will depend on the chipset/implementation of NumPy used, but the times nevertheless serve as guides. A 14-inch Apple Macbook Pro laptop (2021) with an M1 Pro chipset was used for the benchmarking described here. $\textbf{B}$ Variation in approximate memory usage when performing as RelCalc calculation when calculating single quantum A spin relaxation rates for the Platonic solids (Fig. 7). The maximum memory on the benchmarking laptop was 32 Gb. Red crosses indicate calculations that were not run to completion due to infeasibility because of memory (matrix representations) or time ($AX_{12}$/$AX_{20}$ low memory, and $AX_{20}$ symbolic modes) limitations. $\textbf{C}$ The calculation times to evaluate expressions for each single quantum A relaxation rate in the platonic solids AX$_{4}$ to AX$_{12}$ as a function of $N_{O}^{2}$, where $N_{O}$ is the number of operators in the relaxation Hamiltonian for the system. The calculation times scale approximately as $\log (t)=m\log (N_o^2)$. The time taken for symbolic mode exceeds that of a sparse matrix calculation for $AX_{12}$ and beyond, as the number of elements in the symbolic operator increases combinatorially. Nevertheless, as discussed in the text, symbolic mode is always the fastest method to calculate a relaxation rate when the operators $\rho _{s/t}$ can be expressed as single elements by at least one order of magnitude. $\textbf{D}$ The calculation time for relaxation rates run in symbolic mode for each single quantum A relaxation rate, scaling approximately with $\log (t)=m\log (N_pN_o^2)$ where $N_{P}$ is the number of permutations of the operator the relaxation rate and $N_{O}$ is the number of elements in $\rho _{s/t}$

$AX_n$ clusters were arranged with the A spin at the centre, and the *X* spins arranged to form symmetric clusters $AX_4$, $AX_6$, $AX_8$, $AX_{12}$ and $AX_{20}$ adopted the form of the platonic solids, the tetrahedron, octahedron, cube, icosahedron and dodecahedron. AX and XX dipolar interactions, and X CSA interactions were considered for the single quantum Zeeman basis. Results are given in the macromolecular limit (Table 6) and shown exactly versus $\tau _c$ (Fig. 7), together with the symmetries of the corresponding irreducible representations. The coherences corresponding to the highest projected angular momentum ($N_\beta =0$ or *N*) were found to have the slowest relaxation rates, owing to the near-complete cancellation of AX dipolar interactions, making them attractive targets for TROSY experiments.

## Calculation times and memory requirements

A series of computations were performed to benchmark performance of RelCalc. We sought to compute all 65,536 relaxation rates for a $CH_3$ methyl group (including A, X CSA, AX, XX dipolar and A/X dipolar coupling to an external proton). Doing this using symbolic algebra was over an order of magnitude faster than using full matrix representations, which itself is one order of magnitude faster than using sparse matrices (timings are illustrative, computed on a 2021 Macbook pro). Once the symbolic relaxation rates were computed by any method, subsequent recalculation of relaxation rates was significantly faster, taking here just 1.093 seconds (Fig. 8A).

The computations for the platonic solids is substantially more demanding, as the underlying basis matrices have a size of $2^N \times 2^N$. The complex matrices necessary to describe the interactions exceeded 20 Gb for $AX_8$. This is reduced significantly by using sparse matrices, where memory requirements have still exceeded 20 Gb by $AX_{20}$. The symbolic mode does not require matrices to be stored, and so is very memory efficient (Fig. 8B).

An optional low memory symbolic mode is available in RelCalc, which takes each combination of operators in $\rho _s$ and $\rho _t$ independently, as opposed to calculating the double commutators with $\rho _t$ and combining results with the same operator before working through the traces. This formulation minimises memory pressure at the expense of taking significantly longer (Fig. 8C). This provides a route to working with very large spin systems using this framework where memory is a limiting factor.

The calculations for the Platonic solids were designed to test the utility of the symbolic mode to destruction (Fig. 8C). To compute the relaxation rates of the symmetric representation requires looping over $N_O=(N_\alpha +N_\beta )!/N_\alpha !N_\beta !$ individual operators that are effectively treated independently (e.g. $\hat{A}_+\alpha _{10}\beta _{10}$ contains 184,756 terms). This combinatorial requirement results in slower relaxation rates for the symbolic mode than for sparse matrix computation in this specific case between $AX_8$ and $AX_{12}$. While for $AX_3$, the full matrix computation was faster than sparse (though symbolic was substantially faster than both), sparse matrices were faster for all of the computations on the Platonic solids. For $AX_6$ and below, the symbolic mode computes the relaxation rates by an order of magnitude faster than matrix based methods. For the Platonic solids, computation time for relaxation rates using the symbolic mode scales with $\log (t)=m \log (N_pN_O^2)$ (Fig. 8D) where $N_O$ is the number of terms in the operator, and $N_P$ is the number of auto-relaxation rates required ($N+1$). For simulation purposes, when calculating relaxation rates for a complete basis described by single element operators, $\rho _{s/t}$ values will have a single element, the symbolic mode will be the fastest method.

## Discussion

RelCalc is a python engine for rapid symbolic and numerical calculation of relaxation rates for analysing magnetic resonance experiments. Its speed allows relaxation rates for complete Liouvillian evolution matrices to be computed efficiently, then stored and re-used for numerical re-evaluation. The interface requires a user to input minimal details that define the spin system, interactions of interest, together with their orientation in the molecular frame and motional model. A report is optionally generated in LaTex, presenting results in a symbolic form, and modules are present to obtain complete Liouvillian evolution matrices for complete spin physics calculations. The software is highly efficient, and when studying systems of spins $\frac{1}{2}$, the operator algebra is performed symbolically, allowing large spin systems to be analysed with a very low memory footprint.

RelCalc is applied here to investigate a series of relaxation interference effects of experimental interest, including an examination of long-lived singlet states in $X_2$ and TROSY effects in $AX_n$ spin systems. Expressions for relaxation rates are obtained rapidly even when spins are allowed to undergo complex local motions and engage in interactions with external spins within the molecule. In applications to biomolecular NMR, we demonstrate that making the macromolecular approximation, though extremely useful in enabling physical interpretation of relaxation rates, can lead to numerical values that diverge from exact BWR results for molecules with correlation times in the range 1–100 ns.

The results suggest general rules for selecting coherences in TROSY experiments. For symmetric $AX_n$ clusters, high projected angular momentum states will be free of AX dipolar interactions (Eq. B7), such as the $N_+\alpha _4$ resonance in $NH_4^+$ (Flemming Hansen et al. 2009) and the $P_+\alpha _6$ coherence in $PF_6^-$ where the symmetry largely cancels the AX dipolar interactions. The opposite is true for groups rotating about a symmetry axis such as $NH_2$ and $CH_3$ groups in proteins (e.g. $\hat{C}_+\alpha \beta _{2}$ coherence in $CH_3$ methyl groups), where low projected angular momentum states are required and dipolar coupling is minimised simply through having fewer aligned passive spins. In all cases, as noted previously, external spins elsewhere in the molecule capable of cross relaxation act to average out relaxation rates and ‘dilute’ TROSY effects (Jason 2003).

The software is straightforward to extend, so motional models corresponding to alternative spectral density functions can easily be integrated (Levine et al. 1973, 1974; Wittebort and Szabo 1978). While it has become common to analyse relaxation rates from biomolecules using ‘model free’ approaches (Lipari and Szabo 1982), we are hopeful that through careful inspection of relaxation rates and the underlying correlation functions it may still be possible to link relaxation rate measurements more directly to the underlying motions experienced by macromolecules in solution (Philippopoulos et al. 1997; Scott 2007; Dedmon et al. 2005). Templates that calculate relaxation rates for all spin systems described in this manuscript are provided with RelCalc, and a sample script is included (Appendix I). The software is free for academic use and can be downloaded from https://RelCalc.chem.ox.ac.uk.

## Figures and Tables

**Fig. 9:**
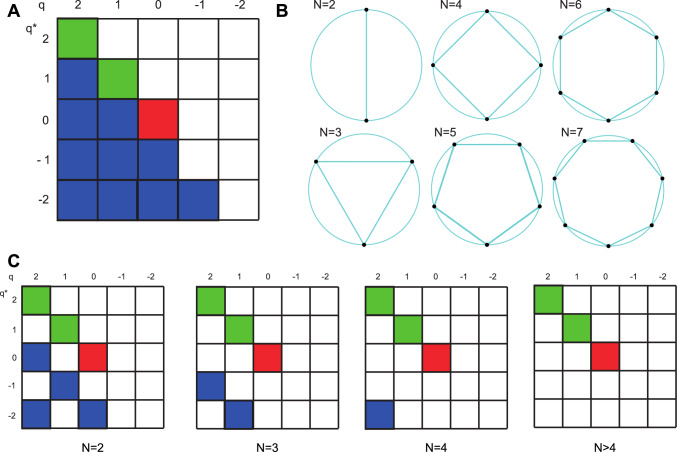
The correlation function requires evaluation of double sums of the form $$\sum _{q,q^*=-r}^r M^{q_0}_{q,q^*}Y_{q}^kY^{l*}_{q^*}$$, which for $$r=2$$ has 25 complex terms, as indicated. Owing to the symmetries in *M* and *Y*, $$(q,q^*)$$ and $$(-q,-q^*)$$ are complex conjugates, which can be combined in to single real Q functions, three with $$q=q^*$$ (red, green) and 10 otherwise, allowing the sum to be written in terms of 13 elements $$\sum _{q=0}^r Q^{q_0}_{q,q} + \sum _{q^*=1}^{q}Q^{q_0}_{q,q^*}$$. The term with $$q=q^*=0$$ is a special case and is already real, so does not need to be combined. Care is taken in the definition of *Q* to ensure that this term is not double counted. When considering rotational motion about a symmetry axis via a hopping rate constant *k* (Appendix C), the acting time-scales are described by regular polygons transcribed in a circle of radius 2*k*, where the vertical position at the vertices indicate the time-scale. This is exactly the result for the energy levels obtained in the quantum mechanical model for a particle ring and is identical to the results obtained for looking at energy levels formed by aromatic $$\pi$$ systems in aromatic rings (Hückel 1933). This picture allows us to replace sums such as $$\sum _\lambda ^Ne^{i\frac{2\pi }{N}\lambda (\beta -\alpha )}$$ with $$2\cos (\frac{2\pi }{N}\lambda (\beta -\alpha ))$$, providing we use $$n_\lambda$$ to deal with the fact that the state will be singly degenerate for $$\lambda =0$$ and if *N* is even, also when $$\lambda =N/2$$ (Eq. C7). When considering the model $$AX-ROT$$, a sum of the above type has to be evaluated, but via the exponential sum formula, different terms end up being taken out of the sum depending on the choice of *N*. Off diagonal terms are retained only of $$\delta _{q-q^*-nN}$$ is satisfied (Eq. B25), where *n* is an integer. For $$N=2,3,4$$, 4, 2 and 1 terms are allowed respectively. For the case $$N>4$$, the situation resembles free diffusion and none of the off diagonal terms are allowed. In this case, the sum contains only terms where $$q=q^**$$ (Eq. B26). These differences are responsible for modest differences in relaxation rates and allow a hopping, in principle, to be distinguished from a freely rotating motion about a symmetry axis.

## Data Availability

No datasets were generated or analysed during the current study.

## Appendix A: Derivation of key results

The relaxation super-operator ${\hat{\hat{\Gamma }}}$ governs the return of the system back to equilibrium which to second order is:

A1 $$\begin{aligned} {\hat{\hat{\Gamma }}}=\int _t^{t+\tau } \langle \left[ \mathcal {H^\textrm{1}}(t),\left[ \mathcal {H}^{1\dagger }(t+\tau ),\right] \right] \rangle d\tau , \end{aligned}$$

where $\mathcal {H^\textrm{1}}$ is the rapidly fluctuating time-dependent Hamiltonian that drives relaxation where $|\mathcal {H}^0|>>|\mathcal {H^\textrm{1}}|$ and $\langle \mathcal {H^\textrm{1}}\rangle =0$. This will be a sum over individual interactions. For a BWR computation we need to represent $\mathcal {H^\textrm{1}}$ in the eigenbasis for chemical shift evolution, and in a form amenable to rotation. We then need to evaluate Eq. 3 using the super-operator in A1 giving relaxation rates of the form stated in Eq. 4.

### Constructing and rotating the interaction Hamiltonian

Interactions relevant for magnetic resonance are of the form

A2 $$\begin{aligned} \mathcal { H} = I \cdot A \cdot S = \textrm{Tr}\left( A \cdot T \right) \end{aligned}$$

where *A* is a second rank spatial tensor, $I=\left( \hat{I}_x,\hat{I}_y,\hat{I}_z\right)$ and $S=\left( \hat{S}_x,\hat{S}_y,\hat{S}_z\right)$ are vectors of spin operators and $T=S\otimes I$, a dyadic (outer) product. Three types of interaction have been implemented that are typically required for solution state relaxation rates, that serve as templates for considering any other. These describe interactions between two nuclei (a bi-linear term such as the dipolar interaction), between a single spin and the static field (a linear term, in the case of CSA where *S* operator is the *B* field) or a single spin interacting with itself (a quadratic term where $S=I$, as is the case of the quadrupolar interaction). The Hamiltonian needs to be in a form that can be easily rotated, which we achieve by projecting both *A* and *T* into the spherical tensor basis, represented by 9 orthonormal matrices $M_q^{(r)}$ (Eq. F3), (Mueller (2011); Pascal (2013)) using $A_p^{(r)}=\textrm{Tr}\left( M_p^{(r)\dag } A\right)$. The Cartesian spatial tensor is then reconstructed using $A= \sum _{r=0}^2\sum _{q=-r}^r A_{q}^{(r)}M_q^{(r)}$. The spherical basis coefficients have the symmetry $A_q^{(r)*} = (-1)^{r-q} A_{-q}^{(r)}$. The Hamiltonian is then described either in a Cartesian ($A_{ij}$ where $ij=xyz$) or the spherical basis (Appendix F),

A3 $$\begin{aligned} \mathcal {H}= \sum _{ij}A_{ij}\hat{T}_{ji} = \sum _{ij }\hat{I}_i A_{ij} \hat{S}_j = \sum _{r=0}^2\sum _{q=-r}^r (-1)^r A_{q}^{(r)} \hat{T}_{q}^{(r)*} \end{aligned}$$

The irreducible tensor operators $\hat{T}_{q}^{(r)*}$ are defined by linear combinations of two spin operators (Table 8). The spherical tensors basis provide a convenient means for implementing rotations. There are conflicting descriptions of their definitions in the literature and text books (discussed e.g. Mueller (2011); Pascal (2013)) and so the conventions used by RelCalc are explicitly stated (Appendix F). The effects of rotations using these definitions is equivalent to rotations performed in Cartesian space using Rodriguez’ formula. A rotation of $\mathcal {H}$ can be an active rotation of the spatial tensor (*A*) or a passive rotation of the basis (*T*). In the spherical tensor basis, Wigner rotation matrices describe a rotation about the ZYZ axes by angles $\alpha$, $\beta$, $\gamma$ respectively, using elements $D_{q,p}^{(r)}(\alpha ,\beta ,\gamma )=e^{-iq\alpha }d_{q,p}^{(r)}(\beta )e^{-ip\gamma }$, where the identities of the ‘small’ elements $d_{q,p}(\theta )$ are widely tabulated (Mueller 2011; Pascal 2013). An active rotation of each element of the spatial tensor is

A4 $$\begin{aligned} R A_q^{(r)}M_q^r R^{-1} = \sum _{p=-r}^r D_{q,p}^{(r)}A_p^{(r)}M_p^r \end{aligned}$$

The rotated Hamiltonian is computed from pairing spatial tensors in the new frame with basis tensors in the original ($\hat{T}_{q}^{(r)*}$),

A5 $$\begin{aligned} \mathcal {H}^\prime = \textrm{Tr}( R A R^{-1} T) = \sum _{r=0}^2 \sum _{qp=-r}^r (-1)^r D_{q,p}^{(r)}A_p^{(r)} \hat{T}_{q}^{(r)*} \end{aligned}$$

To make progress with a BWR calculation we first arrange a general Cartesian spatial tensor in a carefully chosen principle axis frame (PAF), from which we rotate the interaction to line up with the molecular frame and then compute the Hamiltonian. We need the Hamiltonian to be in a form that is easy to rotate, and can be easily rotated to a physical description of the interaction, for example to compare results to a Cartesian tensor from a quantum mechanical computation. In the majority of cases, interactions can simply be approximated as axially symmetric (which is always the case for the dipolar interaction). In this limit, much the following complexity regarding tensor manipulations can be neglected as the tensor reduces to a simple form. We first note a Cartesian tensor can be decomposed into a sum of rank 0, 1 and 2 tensors

A6 $$\begin{aligned} A=A^{(0)}+A^{(1)}+A^{(2)}. \end{aligned}$$

The rank 0 or isotropic component is $A^{(0)}=A^{\textrm{iso}} E$ where *E* is the identity matrix and $A^{\textrm{iso}}=\frac{1}{3}\textrm{Tr}(A)$. The rank 1 asymmetric component is $A^{(1)}=\frac{1}{2}\left( A-A^\dag \right)$, with elements $A_{ij}^{(1)}=-A_{ji}^{(1)}$ and zero on the diagonal. The rank 2 component is symmetric, $A^{(2)}=\frac{1}{2}\left( A+A^\dag \right) -A^{(0)}$ where elements $A^{(2)}_{ij}=A^{(2)}_{ji}$. The components contain 1, 3 and 5 degrees of freedom respectively, and can be visualised as a scalar, a vector and an ellipsoid. We can identify the following 4 magnitudes associated with individual components,

A7 $$\begin{aligned} \begin{array}{rl} A^{\textrm{iso}} & = \frac{1}{3}\left( A_{xx}+A_{yy}+A_{zz}\right) \\ A^{\textrm{as}}& =\sqrt{A_{xy}^{(1)2}+A_{yz}^{(1)2}+A_{xz}^{(1)2}} \\ A^{\textrm{ax}}& =\frac{1}{3}\left( A_{zz}^{(2)}-A_{yy}^{(2)}\right) \\ A^{\textrm{ax2}}& =\frac{1}{3}\left( A_{xx}^{(2)}-A_{yy}^{(2)}\right) \\ \end{array} \end{aligned}$$

where $A_{xx}^{(2)}$, $A_{yy}^{(2)}$ and $A_{zz}^{(2)}$ are the eigenvalues of the second rank tensor (equal to $A_{xx}$, $A_{yy}$ and $A_{zz}$ if the tensor is diagonal and traceless). In BWR computations any isotropic component (e.g. from the chemical shift tensor) is first subtracted and included with $\mathcal {H}^0$. We define the RelCalc PAF to be where either the largest or unique (if axially symmetric) component of the symmetric tensor is aligned with the positive *z* axis (which ensures that the *xz* and *yz* components of the second rank tensor are zero). In this frame, the 1 st and 2nd rank components of the Cartesian tensor can be expressed in terms of irreducible basis matrices $M_q^{(r)}$ (Eq. F3):

A8 $$\begin{aligned} \begin{array}{rl} A^{(2)}& = \sqrt{6} A^{\textrm{ax}} M^{(2)}_0 + \sqrt{6} A^{\textrm{ax2}} R(\frac{\pi }{2},\phi _{ax2}) M^{(2)}_0 R^{-1}(\frac{\pi }{2},\phi _{ax2}) \\ A^{(1)}& =\sqrt{2} A^{\textrm{as}} R(\theta _{as},\phi _{as}) M^{(1)}_0 R^{-1}(\theta _{as},\phi _{as})\\ \end{array} \end{aligned}$$

requiring six parameters; three interaction strengths, $A^{\textrm{ax}}$, $A^{\textrm{ax2}}$ and $A^{\textrm{as}}$, a rotation $\phi _{ax2}$ about the *z* axis that aligns $A_{xx}$ and $A_{yy}$ of $A^{(2)}$ in the *xy* plane (setting $\phi _{ax2}=0$ renders $A^{(2)}$ diagonal), and *yz* rotation angles that orient the asymmetric component vector, $\theta _{as},\phi _{as}$. Where $\theta _{as}=\phi _{as}=0$, then the rank 1 tensor has 1 non-zero component, $A_{xy}^{(1)}=-A_{yx}^{(1)}=A^{\textrm{as}}$.

In relaxation rate computations interactions are typically treated as axially symmetric which provides considerable simplification as the PAF is defined by a single parameter $A^{\textrm{ax}}$ (as $A_{yy}=A_{xx}$ and $A^{\textrm{ax2}}=A^{\textrm{as}}=0$). The dipolar interaction is inherently axially symmetric and so is always of this form. The quadrupolar interaction is traceless and symmetric ($A^{\textrm{iso}}=A^{\textrm{as}}=0$), requiring $A^{\textrm{ax}}$ (and optionally also $A^{\textrm{ax2}}$ and $\phi _{ax2}$). The CSA interaction can require the maximal set of 6 parameters, $A^{\textrm{ax}}$, the 2nd optional symmetric contribution $A^{\textrm{ax2}}$, $\phi _{ax2}$, and a third optional asymmetric contribution $A^{\textrm{as}}$, $\theta _{as}$ and $\phi _{as}$ (which is typically neglected). The next task is to express the Hamiltonian in the irreducible basis ($\hat{T}_q^{(r)*}$, Table 8) and perform an active rotation about the YZ axis using Wigner elements to orient the PAF

A9 $$\begin{aligned} \begin{aligned}&\mathcal {H}^{PAF} =-\sqrt{2} A^{\textrm{as}} \sum _{q=-1}^1 D_{q,0}^{(1)}\left( \theta _{as},\phi _{as}\right) \hat{T}_q^{(1)*}\\&+\sqrt{6} \left( A^{\textrm{ax}} \hat{T}_0^{(2)*} + A^{\textrm{ax2}} \sum _{q=-2}^2 D_{q,0}^{(2)}\left( \frac{\pi }{2},\phi _{ax2}\right) \hat{T}_q^{(2)*}\right) . \end{aligned} \end{aligned}$$

The active Wigner elements are related to the spherical harmonic functions $D_{q,0}^{(r)} = Y_q^{(r)*}$ (Table 1). Then,

A10 $$\begin{aligned} \begin{aligned}&\mathcal {H}^{PAF} =-\sqrt{2} A^{\textrm{as}} \sum _{q=-1}^1 Y_{q}^{(1)*}\left( \theta _{as},\phi _{as}\right) \hat{T}_0^{(1)*} \\&+\sqrt{6} \left( A^{\textrm{ax}} \hat{T}_0^{(2)*} + A^{\textrm{ax2}} \sum _{q=-2}^2 Y_{q}^{(2)*}\left( \frac{\pi }{2},\phi _{ax2}\right) \hat{T}_q^{(2)*}\right) \\ \end{aligned} \end{aligned}$$

Any angular relationships in the molecular frame in relaxation rates are typically described by Legendre polynomials. Spherical harmonics are defined in terms of these polynomials (Table 1), which motivates us defining the Hamiltonian this way. An alternative description in common usage parameterises the symmetric tensor $A^{(2)}$ by $B=2A_{zz}-A_{yy}-A_{xx} = 3 A_{zz}$ and an asymmetry factor $\eta B = \frac{1}{2}(A_{xx}-A_{yy})$. The contribution to the Hamiltonian in the spherical tensor basis (temporarily neglecting $\phi _{ax2}$) of both parameterisations are

A11 $$\begin{aligned} \begin{aligned}&\mathcal {H}^{(2)PAF}(\phi _{ax2}=0) = \sqrt{6} \left( A^{\textrm{ax}}+\frac{1}{2} A^{\textrm{ax2}} \right) \hat{T}_0^{(2)*} \\&+ \frac{3}{2} A^{\textrm{ax2}} \left( \hat{T}_2^{(2)*} + \hat{T}_{-2}^{(2)*}\right) \\&= \sqrt{6} B \hat{T}_0^{(2)*} + \eta B \left( \hat{T}_2^{(2)*} + \hat{T}_{-2}^{(2)*}\right) \end{aligned} \end{aligned}$$

The two parameterisations can be related via the following substitutions $A^{ax}= B\left( 1-\frac{1}{3}\eta \right)$ and $A^{ax2} = B \eta \frac{2}{3}$. Defining $A^{ax}$ and $A^{ax2}$ in terms of differences of eigenvalues of the second rank tensor delivers a physical link to the interaction tensor *A* that emphasises relaxation effects are caused by differences in tensorial components. As spherical harmonics naturally arise from this formulation, it enables computations using their the algebra, which facilitate implementations of symbolic algebra.

The next step is to rotate the Hamiltonian in Eq. A10 into the molecular frame by a further active YZ rotation by angles $\theta _R,\phi _R$,

A12 $$\begin{aligned} \begin{aligned} \mathcal {H^\textrm{1}} =&-\sqrt{2} A^{\textrm{as}} \sum _{q=-1}^1 \hat{T}_q^{(1)*}\sum _{p=-1}^1 D_{q,p}^{(1)}\left( \theta _R,\phi _R\right) Y_{p}^{(1)*}\left( \theta _{as},\phi _{as}\right) \\&+\sqrt{6}\sum _{q=-2}^2 \hat{T}_q^{(2)*} \left( A^{\textrm{ax}}Y_{q}^{(2)*}(\theta _R,\phi _R) + A^{\textrm{ax2}}\sum _{p=-2}^2 \right. \\&\left. D_{q,p}\left( \theta _R,\phi _R\right) Y_{p}^{(2)*}\left( \frac{\pi }{2},\phi _{ax_2}\right) \right) \\ \end{aligned} \end{aligned}$$

To simplify this result we note that the rotation of any spherical harmonic function will be a new spherical harmonic function about adjusted angles (indicated by $\prime$, Appendix E):

A13 $$\begin{aligned} \sum _{q=-r}^r D_{p,q}^{(r)}(\theta _R,\phi _R)Y_{q}^{(r)*}\left( \theta ,\phi \right) = Y_p^{(r)*}(\theta ^{\prime },\phi ^{\prime }) \end{aligned}$$

we can express the completed Hamiltonian in the molecular frame using three sets of spherical harmonics:

A14 $$\begin{aligned} & \mathcal {H} =-\sqrt{2} A^{\textrm{as}} \sum _{q=-1}^1 \hat{T}_q^{(1)*} Y_{q}^{(1)*}\left( \theta _{as^{\prime }},\phi _{as^{\prime }}\right) \nonumber \\ & +\sqrt{6}\sum _{q=-2}^2 \hat{T}_q^{(2)*} \left( A^{\textrm{ax}}Y_{q}^{(2)*}(\theta _R,\phi _R) + A^{\textrm{ax2}} Y_{q}^{(2)*}\left( \theta _{R^{\prime }},\phi _{R^{\prime }}\right) \right) \end{aligned}$$

where $(\theta _{as^{\prime }},\phi _{as^{\prime }})$ performs the same rotation as the two sequential rotations $(\theta _{as},\phi _{as})$ followed by $(\theta _R,\phi _R$), and $(\theta _{R^{\prime }},\phi _{R^{\prime }})$ is the same rotation as from performing $(\frac{\pi }{2},\phi _{ax2})$ followed by $(\theta _R,\phi _R$) (Appendix E), which in the case where $\phi _{ax2}=0$, $\theta _{R^\prime }=\frac{\pi }{2}+\theta _R$ and $\phi _{R^\prime }=\phi _R$. This allows us to write each of the three contributions in a common form

A15 $$\begin{aligned} \mathcal {H}_k= (-1)^r\sqrt{r(r+1)} A_k \sum _{q=-r}^r Y_{kq}^{(r)*}\hat{T}_q^{(r)*}, \end{aligned}$$

specified by an interaction constant ($A^{\textrm{ax}}$, $A^{\textrm{ax2}}$, $A^{\textrm{as}}$) and angles that orient the interaction in the molecular frame ($Y_k$). The next task is to introduce molecular motion, by computing the Hamiltonian at at time *t* following a random rotation

A16 $$\begin{aligned} \mathcal {H}^1_k(t) = (-1)^r \sqrt{r(r+1)} a_k A_k(t) \sum _{p=-r}^r \hat{T}_p^{(r)*} \sum _{q=-r}^r D_{p,q}^{(r)}(t)Y_{kq}^{(r)*} \end{aligned}$$

where we have further separated the interaction constants into a scalar (strength), *a*, a component *A*(*t*) that will account for time dependent variation in distances for the dipolar interaction where relevant (EXT, figure 1, Table 1) and 1 otherwise. By defining a time dependent spatial function for each interaction *k*,

A17 $$\begin{aligned} G_{kp}^{(r)}(t)=A_k(t)\sum _{q=-r}^r D_{p,q}^{(r)}(t) Y_{kq}^{(r)*} \end{aligned}$$

we can write the perturbing Hamiltonian for any interaction *k* at any time *t*,

A18 $$\begin{aligned} \mathcal {H}_k^{1}(t) = (-1)^r \sqrt{r(r+1)} a_k \sum _{p=-r}^r G_{kp}^{(r)}(t) \hat{T}_p^{(r)*}. \end{aligned}$$

If only isotropic tumbling is required and the interactions are static in the molecular frame, *G* contains only one random rotation. Where a more elaborate motional model is required, more rotations are introduced (Table 10), with multiple time-scales indexed by *p*.

Finally, for a BWR calculation we will need to incorporate the evolution of chemical shift. The spherical tensor operators $T^{(r)*}$ are specified by (up to) 9 unique operators $O_i$ (Table 9), each linked to a characteristic evolution frequency ($\omega _i$), a scalar $o_i$ that maps the operator to the irreducible representation and an integer $p_i$ that maps to the projected angular momentum change of the parent spherical tensor, $\hat{T}_p^{(r)*}= \sum _{i=1}^9 \delta _{p_i,p} o_i O_{i}$. The form of the interacting Hamiltonian used by RelCalc is

A19 $$\begin{aligned} \mathcal {H}_{k}^1(t)=\left( -1\right) ^r\sqrt{r(r+1)}a_k \sum _{i=1}^9 o_i \hat{O}_{ki} G_{k,p_i}^{(r)}(t). \end{aligned}$$

where the overall perturbing Hamiltonian $\mathcal {H^\textrm{1}}(t)=\sum _k \mathcal {H}_k^1(t)$. The details are transparent to a user, and each interaction (quadrupolar, dipolar, CSA) is added to a calculation by adding up to 3 terms. These are introduced with relevant constants and angles that orient the interaction in the molecular frame at $t=0$ (symbolic or numerical), together with a specification that the interaction is either static in the molecular frame (ST), there is local motion about a symmetry axis that leads to a variation in e.g. the length of a dipolar vector (EXT) or there is local motion about a symmetry axis where only orientation changes (ROT) that allow the relevant spatial function to be interpreted in terms of spectral densities (Table 10).

**Table 7:** The spherical harmonic functions used by RelCalc to orient interactions in the molecular frame are parameterised by two angles, $\theta _R,\phi _R$, according to $Y_q^{(r)}=y_q^{(r)}z_q^{(r)}$, where $z_q^{(r)}(\theta ,\phi )= P_q^{(r)} e^{+iq\phi }$ and the Legendre polynomials $P_q$ are defined in Table 1

*q*	$y_q^{(1)}$	$y_q^{(2)}$
-2		$\frac{1}{2\sqrt{6}}$
-1	$\frac{1}{\sqrt{2}}$	$\frac{1}{\sqrt{6}}$
0	1	1
1	$-\frac{1}{\sqrt{2}}$	$- \frac{1}{\sqrt{6}}$
2		$\frac{1}{2\sqrt{6}}$

The definition here ensures that $Y_q^{(r)*}=D_{q,0}^{(r)}$, where $D_{q,0}^{(r)}$ is a Wigner matrix rotation element. Two spherical harmonic functions neatly combine according to $\sum _{q=-r}^r Y_q^{(r)} Y_q^{\prime (r)}=P_0^{(r)}(\cos \zeta )$ where $\cos \zeta$ is the dot product of the two unit vectors that orient *Y* and $Y^\prime$

**Table 8:** The complex conjugates of the irreducible rotational representation for $\hat{T}_q^{(r)*}=(-1)^{r-q} \hat{T}_{-q}^{(r)}$ used as basis operators, in terms of two spin operators in the Zeeman basis derived in Appendix F

*q*	$\hat{T}_q^{(1)*}$	$\hat{T}_q^{(2)*}$
-2		$\frac{1}{2}\hat{I}_-\hat{S}_-$
-1	$\frac{1}{2}\left( \hat{I}_-\hat{S}_z - \hat{I}_z\hat{S}_-\right)$	$\frac{1}{2}\left( \hat{I}_z\hat{S}_-+\hat{I}_-\hat{S}_z\right)$
0	$\frac{1}{2\sqrt{2}}\left( \hat{I}_-\hat{S}_+-\hat{I}_+\hat{S}_-\right)$	$\frac{1}{2}\sqrt{\frac{8}{3}}\left( \hat{I}_z\hat{S}_z-\frac{1}{4}\left( \hat{I}_+\hat{S}_-+\hat{I}_-\hat{S}_+\right) \right)$
1	$\frac{1}{2}\left( \hat{I}_+\hat{S}_z - \hat{I}_z\hat{S}_+\right)$	$\frac{1}{2}\left( -\hat{I}_z\hat{S}_+-\hat{I}_+\hat{S}_z\right)$
2		$\frac{1}{2}\hat{I}_+\hat{S}_+$

The numerical co-factors on the operators become the coefficients $o_i$ in Table 9. $r=2$ corresponds to the symmetric part of the interaction tensors, and $r=1$, the anti-symmetric

**Table 9:** The two spin operators and numerical pre-factors that characterise the dipolar (bilinear), chemical shift anisotropy (linear) and quadrupolar (quadratic) perturbing Hamiltonians

*i*	$o_i$	$p_i$	$|\omega _i|$	$\hat{O}_i^{\textrm{dipolar}}$	$\hat{O}^{\textrm{CSA}}_i$	$\hat{O}^{\textrm{quad}}_i$	$\hat{O}_i^{CSA,as}$
1	$\frac{1}{2}$	-2	$\omega _I+\omega _S$	$\hat{I}_-\hat{S}_-$		$\hat{I}_-\hat{I}_-$	
2	$\frac{1}{2}$	-1	$\omega _S$	$\hat{I}_z\hat{S}_-$		$\hat{I}_z\hat{I}_-$	
3	$\frac{1}{2}$	-1	$\omega _I$	$\hat{I}_-\hat{S}_z$	$\hat{I}_-$	$\hat{I}_-\hat{I}_z$	$\hat{I}_-$
4	$\frac{1}{2}\sqrt{\frac{8}{3}}$	0	0	$\hat{I}_z\hat{S}_z$	$\hat{I}_z$	$\hat{I}_z\hat{I}_z$	
5	$-\frac{1}{8}\sqrt{\frac{8}{3}}$	0	$\omega _I-\omega _S$	$\hat{I}_+\hat{S}_-$		$\hat{I}_+\hat{I}_-$	
6	$-\frac{1}{8}\sqrt{\frac{8}{3}}$	0	$\omega _I-\omega _S$	$\hat{I}_-\hat{S}_+$		$\hat{I}_-\hat{I}_+$	
7	$-\frac{1}{2}$	1	$\omega _S$	$\hat{I}_z\hat{S}_+$		$\hat{I}_z\hat{I}_+$	
8	$-\frac{1}{2}$	1	$\omega _I$	$\hat{I}_+\hat{S}_z$	$\hat{I}_+$	$\hat{I}_+\hat{I}_z$	$\hat{I}_+$
9	$\frac{1}{2}$	2	$\omega _I+\omega _S$	$\hat{I}_+\hat{S}_+$		$\hat{I}_+\hat{I}_+$	

All interactions causing relaxation in NMR can be expressed as a sum of (up to) nine operators ($\hat{O}_{i}$) in the Zeeman basis (Eq. A19). Each term is associated with a factor $o_i$ and index $p_i$ that relate the operator to the irreducible representation (Table 8), and a characteristic evolution frequency $\omega _{i}$. When expressed in this form, Hamiltonians can be both easily rotated, and evolved under chemical shift, features needed for progress in a BWR calculation. The CSA interaction has the spin of interest coupling to the static magnetic field assumed along the *z* axis, and so only terms in $\hat{S}_z$ appear. In the case of the quadrupolar interaction, $\omega _S=\omega _I$

**Table 10:** The time dependent spatial functions ($G_{ki}^{(r)}(t)$) that rotate interaction *k* from the PAF into a laboratory frame

Local motion	Tumbling	$G_{ki}^{(r)}(t)$
Static	Isotropic	$A_k(t)\sum _{q=-r}^r D_{p_i,q}^T(t) Y_{q}^{k*}$
Rotating	Isotropic	$A_k(t)\sum _{q=-r}^r D_{p_i,q}^T(t) D_{q,q}^R(t) Y_{q}^{k*}$
Static	Axially symmetric	$A_k(t)\sum _{q,q_o=-r}^r D_{p_i,q_o}^T(t) D_{q_o,q}^O Y_{q}^{k*}$
Rotating	Axially symmetric	$A_k(t)\sum _{q,q_o=-r}^r D_{p_i,q_o}^T(t) D_{q_o,q}^O D_{q,q}^R(t) Y_{q}^{k*}$

$Y_q^{k*}$ rotates from the PAF into the molecular frame ($\theta _M,\phi _M)$, $D_{q,q}^R$ is a random time dependent rotation about a symmetry axis ($\phi _R$), $D_{q_o,q}^O$ orients the molecular frame (and symmetry axis) into the diffusion frame ($\theta _O,\phi _O$), and $D_{a,b}^T$ describes time dependent random molecular tumbling. The index $p_i$ reflects the value for the *i*th component of interaction *k* (Table 9). The diagonal Wigner elements for rotation about the symmetry axis in the molecular frame are characterised by a single angle, $D_{q,q}=e^{-iq\phi }$. $A_k$ is either equal to $\frac{1}{r^3}$ if *k* is a dipolar interaction and it varies with the molecular motion or unity otherwise (Table 1). This distinction allows for $r^3$ to be optionally time-dependent, as encountered in the case where the distance characterising a dipolar interaction varies due to rotation about a symmetry axis (EXT)

### Evaluation of the relaxation super-operator

With the relaxation Hamiltonian defined by summing over Eq. A19 we are ready to evaluate Eq. 3 using Eq. A1. This requires a double sum over first all pairs of terms in $\mathcal {H^\textrm{1}}$ (*kl*), and all combinations of their (up to) 9 constituent operators (*ij*) resulting in a quadruple sum

A20 $$\begin{aligned} R_{st} = \left\langle \rho _s\right| {\hat{\hat{\Gamma }}} \rho _t\rangle =\sum _{ijkl} R_{ki, lj}^{st}, \end{aligned}$$

where each pair of *ki* and *lj* contributes

A21 $$\begin{aligned} & R_{ki,lj}^{st}= r(r+1) \delta _{r_k,r_l} a_ka_lo_io_j \int _t^{t+\tau }\nonumber \\ & \langle \rho _s| \left[ G_{lj}^{(r)}(t) e^{i\mathcal {H}^0t}\hat{O}_{lj}e^{-i\mathcal {H}^0t},\left[ G_{ki}^{(r)*}(t+\tau ) e^{i\mathcal {H}^0(t+\tau )} \hat{O}_{ki}^\dagger e^{-i\mathcal {H}^0(t+\tau )},\rho _t\right] \right] \rangle d\tau . \end{aligned}$$

The rank *r* of *k* and *l* will be either 1 (anti-symmetric component) or 2 (symmetric component) and $\delta _{r_k,r_l}$ reflects the condition (to be derived shortly) that the ensemble averages over rotation operators will be zero unless the tensorial ranks of *k* and *l* are identical. As the operators $\hat{O}$ are in the chemical shift eigenbasis, evaluating the evolution under $\mathcal {H}^0$ is straightforward: $e^{i\mathcal {H}^0\tau } \hat{O}_{ki}^\dagger e^{-i\mathcal {H}^0\tau }= \hat{O}_{ki}^\dagger e^{-i\tau \omega _{ki}}$. Applying the assumption that evolution of the spin operators will be slow when compared to decay of the correlation functions (the ’weak collision’ limit), the spatial and spin parts can be separated. Defining the correlation function between two spatial functions as $C_{ki,lj}^{(r)}(\tau )=(2r+1)\langle G_{lj}^{(r)}(t)G_{ki}^{(r)*}(t+\tau )\rangle$, we obtain:

A22 $$\begin{aligned} & R_{ki,lj}^{st}= \frac{r(r+1)}{2r+1} a_ka_lo_io_j \langle e^{it(\omega _{lj}-\omega _{ki})} \rangle \nonumber \\ & \left( \int _t^{t+\tau } C_{ki,lj}^{(r)}(\tau ) e^{-i\tau \omega _{ki}} d\tau \right) \langle \rho _s| \left[ \hat{O}_{lj},\left[ \hat{O}_{ki}^\dagger , \rho _t\right] \right] \rangle \end{aligned}$$

The ensemble average of frequencies will rapidly average to zero unless $\omega _{lj}=\omega _{ki}$ and so $\langle e^{it(\omega _{lj}-\omega _{ki})}\rangle = \delta _{\omega _{ki},\omega _{{lj}}}$. Defining the overall spectral density function as

A23 $$\begin{aligned} J_{ki,lj}^{\prime (r)}\left( \omega _{ki}\right) = \int _t^{t+\tau } C_{ki,lj}^{(r)}(\tau ) e^{-i\tau \omega _{ki}} d\tau \end{aligned}$$

our final expression for the relaxation rate is

A24 $$\begin{aligned} R_{ki,lj}^{st}= \frac{r(r+1)}{2r+1} a_ka_lo_io_j \delta _{r_k,r_l}\delta _{\omega _{ki},\omega _{{lj}}} J^{\prime (r)}_{ki,lj}\left( \omega _{ki}\right) S_{ki,lj}^{st} \end{aligned}$$

where the selection factors $S_{ki,lj}^{st}$ requires evaluation of the trace of a double commutator

A25 $$\begin{aligned} S_{ki,lj}^{st}= \langle \rho _s| \left[ \hat{O}_{lj},\left[ \hat{O}_{ki}^\dagger , \rho _t\right] \right] \rangle \end{aligned}$$

Because of the number of evaluations required, this is the most demanding aspect of a BWR calculation. Each non-zero $R_{ki,lj}^{st}$ term is defined by a series of symbolic constants, numerical factors (including $S_{ki,lj}^{st}$) and a symbolic spectral density. The spectral density function $J^\prime$ depends on the motional model (Table 12), those of which incorporated into RelCalc are explicitly derived over the next sections. RelCalc can be readily expanded to include new motional models as required.

## Appendix B: Derivation of the spectral density functions

We evaluate the form of the correlation functions for the various motional models (Table 12) and then calculate their corresponding spectral density functions in terms of sums of reduced spectral density functions, revealing that all BWR relaxation rates with these motional models will take the form of Eq. 5.

### Correlation functions

We first apply the final two assumptions of BWR theory: that ‘noise’ is stationary, and so the correlation does not depend on the choice of *t*, and the cross-correlation function decays to zero faster than the time taken for the spin evolution, allowing us to set $t=0$ and extend the upper limit of the integration to $\infty$. Noting that (typically very small) imaginary parts of the Fourier transform in Eq. A23 are subtracted and factored into $\mathcal {H}^0$ where they contribute to the chemical shift as ‘dynamic frequency shifts’ and so we retain only the real parts of the Fourier transform

B1 $$\begin{aligned} \begin{aligned} J'(\omega )&=\int _0^{\infty } C (\tau ) \cos \left( \tau \omega \right) d\tau \\ \end{aligned} \end{aligned}$$

There are two independent, time dependent sources of random motion analysed by RelCalc, the effects of global tumbling, and the effects of local motion about a symmetry axis. As will be proven shortly (Appendix B3), and as noted by Lipari and Szabo (1982), all motional models considered here result in a sum over *n* exponential decays with distinct time constants ($\tau _n$)

B2 $$\begin{aligned} C(\tau )=C(0) \sum _nS_n e^{-\frac{\tau }{\tau _n}} \end{aligned}$$

Where *C*(0) describes the relative orientation of interactions at $\tau =0$, and the order parameters $S_n$ sum to unity, $\sum _nS_n=1$. With this general definition for the spectral density, we can evaluate $J'(\omega )$ (Eq. B1):

B3 $$\begin{aligned} \begin{aligned} J'(\omega )&=C(0)\sum _n \int _0^{\infty } S_{n}e^{-\frac{\tau }{\tau _n}}\cos \left( \tau \omega \right) d\tau =C(0) \sum _n S_{n} J(\tau _n,\omega )\\ \end{aligned} \end{aligned}$$

where we finally recover the reduced spectral density function, *J* (Eq. 5). The number of time-scales *n* and their values $\tau _n$ will depend on the specific motional model, but each, together with a weighting $C(0)S_n$ contributes one reduced spectral density term $J(\tau ,\omega )$ to the final relaxation rate. This demonstrates relaxation rates will be of the form of Eq. 4. Our final task is to examine the specific motional models (Table 12) and prove that they adopt the form given by Eq. B2.

### The isotropic, static correlation function and spectral density

We first analyse the simplest case, where global tumbling is isotropic, and where there is no local motion in the molecular frame (ISO ST, Table 10), which is the form most commonly encountered,

B4 $$\begin{aligned} \begin{aligned} C_{ki,lj}^{ISO,ST}(\tau )= (2r+1) A_kA_l\langle \sum _{q,q^*=-2}^2 D_{p_i,q}^{(r)*}(\tau )D^{(r)}_{p^*_j,q^*}(\tau ) Y_q^{k}Y_{q^*}^{l*} \rangle .\\ \end{aligned} \end{aligned}$$

The ensemble average for isotropic global tumbling evaluates as $\langle D^{(r)}_{p,q}D^{(r)*}_{p^*,q^*} \rangle =\frac{1}{2r+1}e^{-r(r+1)D_R\tau }\delta _{r,r^*}\delta _{p,p^*}\delta _{q,q^*}$ where $D_R$ is the rotational diffusion coefficient (Woessner 1962). The rotational correlation time $\tau _c=\frac{1}{6D_R}=\frac{4\pi \eta R_H^3}{3kT}$ where $R_H$ is the hydrodynamic radius, and $\eta$ is the dynamic viscosity, and

B5 $$\begin{aligned} \begin{aligned} C_{ki,lj}^{ISO,ST}(\tau )= A_kA_l e^{-\frac{\tau }{\tau _{c}}} \sum _{q=-r}^r Y_q^kY_{q}^{l*} \\ \end{aligned} \end{aligned}$$

Finally, using the spherical addition formula, $\sum _{q=-r}^r Y^{k(r)}_qY^{l(r)*}_q=P_0^{(r)}(\cos \zeta _{kl})$, where $\cos \zeta _{kl}$ is the dot product between the two normal vectors that orient *k* and *l* and writing $\tau _c$ in terms of the generalised time-scale (Eq. 7),

B6 $$\begin{aligned} C_{ki,lj}^{ISO,ST}(\tau )= A_kA_l e^{-\frac{\tau }{\tau (0,0)}} P_0\left( \cos \zeta _{kl}\right) . \end{aligned}$$

Taking the real Fourier transform (Eq. B1) reveals the reduced spectral density (Eq. 5)

B7 $$\begin{aligned} J_{ki,lj}^{\prime ISO,ST}= A_kA_l P_0\left( \cos \zeta _{kl}\right) J(0,0,\omega _{ki}) \end{aligned}$$

Which in the macromolecular limit ($\frac{1}{\tau _c}>> \omega _{ki}$) further reduces to $A_kA_l P_0\left( \cos \zeta _{kl}\right) \tau _c$. Finally, when $k=l$, this contribution is simply $A_k^2\tau _c$. The remaining five expressions for $J^\prime$ (Table 12) will now be derived.

### Model specific correlation functions

**Table 11:** Values of the rotational coefficients used to described anisotropic global diffusion, defined as $M^{q_o}_{q,q_2}=(1-\frac{1}{2}\delta _{q_0,0})(d_{q_0,q}d_{q_0,q_2}+d_{-q_0,q}d_{-q_0,q_2})$ for cases where $q=q_2$ for rank 2 interactions used for rotating interactions from the molecular frame to the diffusion frame

$M_{q,q}^{q_o}$	$q=0$	1	2
$q_o=0$	$P_0^2$	$\frac{1}{6}P_1^2$	$\frac{1}{24}P_2^2$
1	$\frac{1}{3} P_1^2$	$\frac{2}{18}(8P_0^2-P_0+2)$	$\frac{1}{9} P_2(2+P_0)$
2	$\frac{1}{12}P_2^2$	$\frac{1}{9} P_2(2+P_0)$	$\frac{1}{18}(P_0^2+10P_0+7)$

These are expressed in terms of Legendre polynomials, parameterised by $\cos \theta _O$, the polar rotation angle into the diffusion frame. In the absence of local motion, $q=0$, and $M_{0,0}^0=\frac{1}{4}\left( 3\cos \theta _O-1\right) ^2$, $M_{0,0}^1=\frac{3}{4}\sin ^2(2\theta _O)$, $M_{0,0}^2=\frac{3}{4}\sin ^4\theta _O$ (encountered e.g in Bouguet et al. (2005)). Following from properties of the Wigner matrix elements, $\sum _{q_0=0}^2M^{q_o}_{p,q}=\delta _{p,q}$, $M^{q_0}_{q,q2}=(-1)^{q+q2}M^{q_0}_{-q,-q2}$ and $M^{q_0}_{q,q2}=M^{q_0}_{q_2,q}$

We seek spectral density functions that include the effects of motion in the molecular frame and anisotropic tumbling. We assume first that local motion is entirely uncorrelated with global motion, allowing us to deal with the two parts separately. Firstly, we have an ensemble average over global tumbling to evaluate. Including the cases where we have either isotropic or axially symmetric tumbling we have the following to evaluate, indexed *ki*, *lj*Woessner (1962),

B8 $$\begin{aligned} & \langle (2r+1) \sum _{q_o,q_o^*=-2}^2 D^{T(r)}_{p_i,q_o}D^{T(r)*}_{p_j^*,q_o^*} \rangle = \delta _{r,r^*}\delta _{p_i,p_j^*}\delta _{q_o,q_o^*}\nonumber \\ & \sum _{q_o=-r}^r e^{-\left( r(r+1)D_{\perp }+q_o^2\left( D_\perp -D_\parallel \right) \right) \tau }=\delta _{r,r^*}\delta _{p_i,p_j^*}\delta _{q_o,q_o^*}\sum _{q_o=-r}^r e^{-\frac{\tau }{\tau \left( q_0,0\right) }} \end{aligned}$$

where $\tau _{c}$ is either the globally isotropic tumbling time 1/6*D*, or the correlation time of the unique axis for axially symmetric rotation $1/6D_\perp$. We define the time-scale $\tau _{ax}=\frac{1}{D_{||}-D_{\perp }}=6\left( \tau _{\perp }^{-1}-\tau _{c}^{-1}\right) ^{-1}=6\tau _c\left( \frac{1}{\sigma }-1\right) ^{-1}$ where $\sigma =\frac{\tau _{\parallel }}{\tau _c}$. When we approach the isotropic tumbling limit, $1/\tau _{ax}$ tends to zero and the generalised time-scale (Eq. 7) returns to the isotropic limit. The most general correlation function considered in this work, including anisotropic diffusion effects and having applied $\delta _{r,r^*}\delta _{p_i,p_j^*}\delta _{q_o,q_o^*}$ (where $D^O$ orients the molecular frame with the tumbling frame) is

B9 $$\begin{aligned} \begin{aligned} C_{ki,lj} (\tau )&= \sum _{q_o=-r}^r e^{-\frac{\tau }{\tau \left( q_0,0\right) }} \sum _{q^*,q=-r}^r D_{q_o,q}^{O*}D_{q_o,q^*}^{O}\\&\langle A^k(0) A^l(\tau )Y_q^k(0)Y_{q^*}^{l*}(\tau )\rangle \\ \end{aligned} \end{aligned}$$

We will consider a local motion where atoms follow a circular motion about *N* sites along a symmetry axis and express the ensemble average over the local motion in terms of the time dependent probability of arriving at site $\beta$ given starting on site $\alpha$, $p_{\alpha \beta }(\tau )$ (Appendix C),

B10 $$\begin{aligned} \begin{aligned} C_{ki,lj} (\tau )&= \sum _{q_0=-r}^r e^{-\frac{\tau }{\tau (q_o,0)}} \sum _{q^*,q=-r}^r D_{q_o,q}^{O*}D_{q_o,q^*}^{O}\sum _{\alpha ,\beta } \\&\frac{1}{N^2} p_{\beta \alpha }(\tau ) A^k_\beta A^l_\alpha Y_{q,\beta }^kY_{q^*,\alpha }^{l*} \\ \end{aligned} \end{aligned}$$

As various quantities in this function are complex, it isn’t obvious that the correlation function will be real. We will first prove that this is the case and reduce the number of terms (from currently 125 for $r=2$) by combining terms in the triple sum ($q_0,q,q^*$) that are complex conjugates of each other into real elements. We will do this in two stages, first combining terms $\pm q_0$ with constant $(q,q^*)$ that collects the rotations into the diffusion frame into a new functions $M^{q_0}_{q,q^*}$ (Table 11), and then combining these in $(\pm q,\pm q^*)$ pairs creating a new real function $Q_{q_0,q,q^*}$. These reduce the number of terms in the sum to 13 for $r=2$ (Figure 9A).

First, we separate the Wigner function that aligns the molecular frame with the diffusion frame ($D^O_{q_o,q}=e^{-i q_0\phi _O}d_{q_o,q}$) into its ’small’ element $d_{q_o,q}$, that is real and depends only on $\theta _O$ Wigner (1931) and the complex exponential that describes rotation about the *z* axis. As these occur already as products of complex conjugates with the same $q_0$, the complex part will be $e^{i(q_0-q_0)\phi _o}=1$ and so there will be no dependence on $\phi _O$. We can combine the remaining small elements in $\pm q_o$ pairs and define a new function handling the rotation into the diffusion frame

B11 $$\begin{aligned} M^{q_0}_{q,q_2}=\left( 1-\frac{1}{2}\delta _{q_0,0}\right) \left( d_{q_o,q}^Od_{q_o,q_2}^{O*}+d_{-q_o,q}^Od_{-q_o,q_2}^{O*}\right) \end{aligned}$$

where the sum over $q_0$ goes from 0-2 and the $\delta$ function will prevent double counting of the $q_0=0$ term (Table 11), reducing the sum to 75 terms:

B12 $$\begin{aligned} \begin{aligned} C_{ki,lj} (\tau )&= \sum _{q_0=0}^r e^{-\frac{\tau }{\tau \left( q_o,0\right) }} \sum _{q^*,q=-r}^r M_{q,q^*}^{q_o} \sum _{\alpha ,\beta } \frac{1}{N^2} p_{\beta \alpha }(\tau ) A^k_\beta A^l_\alpha Y_{q,\beta }^kY_{q^*,\alpha }^{l*} \\ \end{aligned} \end{aligned}$$

With this definition, $\sum _{q_o=0}^rM^{q_o}_{q,q^*}=\delta _{q,q^*}$ (arising from the closure relation for Wigner elements Wigner (1931)) and we can immediately reduce to the isotropic limit $\sum _{q_0=0}^r e^{-\frac{\tau }{\tau \left( q_o,0\right) }} M_{q,q^*}^{q_o} =e^{-\frac{\tau }{\tau _c}}\delta _{q,q^*}$. To combine the remaining elements, we can picture the double sum (25 elements for $r=2$) over $q,q^*$ as a square (Fig. 9A). If we combine terms $(q_o,q,q^*)$ and $(q_o,-q,-q^*)$ then the complex parts of the spherical harmonics will combine with their complex conjugates, revealed by defining a new real function that handles combined rotation into the molecular frame and then the diffusion frame,

B13 $$\begin{aligned} \begin{aligned} Q_{q_o,q,q_2}^{kl}&= \left( 1-\frac{1}{2}\delta _{q_2,0}\delta _{q,0}\right) \left( M^{q_0}_{q,q_2}Y^k_qY^{l*}_{q^*} + M^{q_0}_{-q,-q_2}Y^k_{-q}Y^{l*}_{-q_2}\right) \end{aligned} \end{aligned}$$

where the $\delta$ function prevents double counting of the case where $q_2=q=0$. Because $M^{q_0}_{q,q^*}=(-1)^{q+q^*}M^{q_0}_{-q,-q^*}$ and $(-1)^{q+q^*} y_qy_{q^*}=y_{-q}y_{-q^*}$, we can take the complex part of the spherical harmonics and create a real cosine

B14 $$\begin{aligned} \begin{aligned} Q_{q_o,q,q_2}^{kl}&= \left( 2-\delta _{q,0}\delta _{q_2,0}\right) y_qy_{q_2} P^k_{q}P^l_{q_2} M^{q_o}_{q,q_2} \cos \left( q\phi _{k}-q_2\phi _{l}\right) \\ \end{aligned} \end{aligned}$$

where $P_q$ are the real Legendre polynomials. This factorisation reduces the sum to 13 elements comprising three terms where $q=q^*$ (red, green) and the ten remaining combinations that form the off-diagonal triangle (blue) (Fig. 9A). The triple sum has been reduced in the $r=2$ case from 125 complex terms, to 39 real ones. A further simplification can be achieved by noting

B15 $$\begin{aligned} \begin{aligned} Q_{q_o}^{\prime kl}=\sum _{q=0}^r Q_{q_o,q,q}^{kl} + \sum _{q=-r}^r\sum _{q^*=q+1}^rQ_{q_o,q,q^*}^{kl} \end{aligned} \end{aligned}$$

which states that two consecutive rotations (into the diffusion frame and into the molecular frame), starting from the positive *z* axis, can be replaced by a single rotation with composite ‘effective’ angles $\theta _{\textrm{eff}}$/$\phi _{\textrm{eff}}$ (indicated by the $\prime$, expressions for which are in section E), which follows from the spherical harmonic relation $\sum _{q=-r}^rD_{q_o,q}^OY_{q}=Y^\prime _{q_o}$. Taken together, the correlation function has been simplified

B16 $$\begin{aligned} \begin{aligned}&\sum _{q_o,q,q^*=-r}^r D^{O*}_{q_o,q}D^O_{q_o,q^*}Y^k_qY^{l*}_{q^*}= \sum _{q_0=0}^r \sum _{q,q^*=-r}^r M^{q_0}_{q,q^*} Y^k_qY^{l*}_{q^*}\\ =&\sum _{q_o=0}^r\left( \sum _{q=0}^r Q_{q_o,q,q}^{kl} + \sum _{q=-r}^r\sum _{q^*=q+1}^rQ_{q_o,q,q^*}^{kl}\right) =\sum _{q_0=0}^r Q_{q_o}^{\prime kl} \end{aligned} \end{aligned}$$

In the case of isotropic tumbling, the time-scale no longer depends on $q_0$ and the closure relation can be applied leading to

B17 $$\begin{aligned} \sum _{q_0=0}^r Q^{kl}_{q_0,q,q} = (1-\frac{1}{2}\delta _{q,0})\left( Y_q^kY_q^{l*}+Y_{-q}^kY_{-q}^{l*}\right) = Q_{q}^{kl} \end{aligned}$$

which can be written in terms of Legendre polynomials

B18 $$\begin{aligned} \begin{aligned} Q_{q}^{kl}&= \left( 2-\delta _{q,0}\right) y_q^2 P^k_{q}P^l_{q} \cos \left( q(\phi _{k}-\phi _{l})\right) \\ \end{aligned} \end{aligned}$$

which retain the property $\sum _{q=0}^r Q_q^{kl}=P_0\left( \cos \zeta _{kl}\right)$. This result allows us to re-write the correlation function where the indices $\alpha$ and $\beta$ indicate that the interaction should be placed in the molecular frame, adjusted to the specified point in the local motion, then rotated into the diffusion frame, allowing the general correlation function to be reduced to a sum containing 3 real elements (for $r=2$)

B19 $$\begin{aligned} \begin{aligned} C_{ki,lj} (\tau )&= \sum _{q_0=0}^r e^{-\frac{\tau }{\tau (q_0,0)}} \sum _{\alpha ,\beta } \frac{1}{N^2}A^k_\beta A^l_\alpha p_{\beta \alpha }(\tau ) Q_{q_o}^{\prime kl,\beta \alpha } \end{aligned} \end{aligned}$$

Finally, we can insert $p_{\alpha \beta }(\tau )$ which gives us the time dependent probability of the system arriving in position $\beta$ having started at position $\alpha$ which requires $3\Lambda$ unique time-scales (Appendix C), where $\Lambda$ is given by $\textrm{floor}((N+1)/2)$. Merging the time-scale with the generic time-scale used in this work (Eq. 7), we finally obtain the explicit form of the most general correlation function in RelCalc describing local motion following *N* steps around a circle, where the interaction vector changes with length depending on the motion,

B20 $$\begin{aligned} \begin{aligned}&C_{ki,lj}^{AX,EXT}(\tau ) = \sum _{q_o=0}^r \sum _{\lambda }^\Lambda e^{-\frac{\tau }{\tau (q_o,\lambda )}}\\&\sum _{\beta ,\alpha }^N\frac{n_\lambda }{N^2} A^k_\beta A^l_\alpha \cos \left( \frac{2\pi }{N}\lambda (\beta -\alpha )\right) Q^{\prime kl,\beta \alpha }_{q_o} \end{aligned} \end{aligned}$$

Equation B20 is implemented in RelCalc as $AX-EXT$ (Table 12). This is evaluated numerically in RelCalc requiring spherical polar co-ordinates $(r,\theta ,\phi )$ for the initial position of the two interacting atoms. The vectors describing the interactions between *k* and *l* are computed and averaged over the trajectory (section D). This most general form is required for, for example, a methyl proton undergoing free rotation being relaxed by dipolar coupling to a static proton in the molecular frame, where the overall molecular tumbling is axially symmetric.

We will now apply simplifications to the correlation function to obtain the remaining models. In the case of isotropic tumbling $1/\tau _{ax}=0$, and so the time-scale is no longer dependent on $q_o$. As $\sum _{q_0=0}^r M^{q_o}_{q,q^*}=\delta _{q,q_o}$, and following the addition formula for spherical harmonics, $\sum _{q=0}^rQ_q=P_0\left( \cos \zeta \right)$ we obtain the following, implemented in RelCalc as $ISO-EXT$.

B21 $$\begin{aligned} \begin{aligned}&C_{ki,lj}^{\textrm{ISO,EXT}}(\tau ) = \sum _{\lambda }^\Lambda e^{-\frac{\tau }{\tau (0,\lambda )}} \\&\sum _{\beta ,\alpha }^N \frac{n_\lambda }{N^2}\cos \left( \frac{2\pi }{N}\lambda \left( \beta -\alpha \right) \right) A^k_\beta A^l_\alpha P_0\left( \cos \zeta _{kl,\beta \alpha }\right) \end{aligned} \end{aligned}$$

If the interaction strengths, $A^k$ and $A^l$ and the angle between the vectors that orient *k* and *l* is independent of $\beta$ and $\alpha$, such as the AX or XX dipolar interactions in an AX$_n$ model undergoing hopping about the symmetry axis where there is no change in the length of the vector during the motion, we can simplify the expressions considerably. Returning to Eq. B12, in the spherical harmonic functions we can separate the rotation about the *z* axis and insert explicit expressions for the rotations about $\alpha$ and $\beta$

B22 $$\begin{aligned} \begin{aligned} C_{ki,lj} (\tau )&= A^k A^l \sum _{q_0=0}^r e^{-\frac{\tau }{\tau \left( q_o,0\right) }} \sum _{q^*,q=-r}^r M_{q,q^*}^{q_o} Y_{q}^kY_{q^*}^{l*}\\&\sum _{\alpha ,\beta } \frac{1}{N^2} p_{\beta \alpha }(\tau ) e^{i\frac{2\pi }{N}(q\beta - q^*\alpha )} \\ \end{aligned} \end{aligned}$$

The angular terms and the probability can be evaluated together

B23 $$\begin{aligned} & \sum _{\alpha ,\beta =0}^{N-1}\frac{1}{N^2}p_{\alpha \beta }(\tau )e^{i \frac{2\pi }{N}( q\beta - q^*\alpha ) } = \frac{1}{N^2}\sum _{\lambda =0}^{N-1} e^{-\frac{\tau }{\tau _m(\lambda )}}\nonumber \\ & \sum _{\alpha ,\beta =0}^{N-1} e^{i \frac{2\pi }{N}(\beta (\lambda +q)-\alpha (\lambda +q^*))} \end{aligned}$$

The exponential sums over $\alpha$ and $\beta$ form a geometric series around a circle. This will be zero unless $\lambda +q=nN$ and $\lambda +q^*=mN$ where *n* and *m* are integers. These impose $q-q^*=nN$ and $\lambda =\frac{m}{2}N-q$. Because $\tau _m(\lambda )=\tau _m(-\lambda )$ and $\tau (\lambda )=\tau (N-\lambda )$, the latter condition can be simplified to $\lambda =-q$ and

B24 $$\begin{aligned} \begin{aligned} C_{ki,lj} (\tau )&= A^k A^l \sum _{q_0=0}^r\sum _{q=-r}^r e^{-\frac{\tau }{\tau \left( q_o,q \right) }} \\&\sum _{n,q^*=-r}^r M_{q,q^*}^{q_o} Y_{q}^kY_{q^*}^{l*} \delta _{(q-q^*-nN)}\\ \end{aligned} \end{aligned}$$

where the sum over the integer *n* ensures all possible combinations are retained. Combining the terms as before ($\pm q,\pm q^*$) we arrive at the correlation function implemented as ‘AX-ROT’

B25 $$\begin{aligned} \begin{aligned}&C_{kl}^{\textrm{AX,ROT}}(\tau ) = A^k A^l \sum _{q,q_o=0}^r e^{-\frac{\tau }{\tau (q_o,q)}}\\&\left( Q_{q_o,q,q}^{kl}+\sum _{q^*=-r}^r\sum _{n=1}^r\delta _{(q-q^*-nN)}Q_{q_o,q,q^*}^{kl}\right) \end{aligned} \end{aligned}$$

The delta function selects for ‘allowed’ rotational terms. For all values of $N>4$ the delta function will always be zero (which includes the case where the group is freely rotating and $N\rightarrow \infty$), and there will be just 3 $Q_{q_0,q,q}$ terms and 9 discrete time-scales. For $N=4$ one additional term appears ($q=2,q^*=-2,n=1$), for $N=3$ there are two, ($q=2,1$, $q^*=-1,-2$, $n=1,1$) and for $N=2$ there are four (Fig. 9c, blue). Thus simple site hopping with $N<=4$, and freely diffusing motion ($N\rightarrow \infty$) are in principle distinguishable as they will lead to marginally different expressions for relaxation rates. In the freely diffusing limit,

B26 $$\begin{aligned} \begin{aligned} C_{kl}^{\textrm{AX,ROT,}N\rightarrow \infty }(\tau )&= A^k A^l \sum _{q,q_o=0}^r e^{-\frac{\tau }{\tau (q_o,q)}} Q_{q_o,q,q}^{kl} \end{aligned} \end{aligned}$$

and within the time-scale function, $\frac{1}{\tau _m(q)}=\frac{q^2}{2\tau _{\text {Diff}}}$ where $\tau _{\text {diff}}=\frac{1}{2k}\left( \frac{N}{2\pi }\right) ^2$ is the lifetime per squared angle, a measure of diffusion in a ring. In RelCalc, freely diffusion or *N* site hopping can be specified. If we impose isotropic global tumbling, the condition $\delta _{q,q^*}$ is immediately imposed (Eq. B8) and we obtain ‘ISO-ROT’

B27 $$\begin{aligned} \begin{aligned}&C_{kl} ^{\textrm{ISO,ROT}}(\tau ) = A^k A^l\sum _{q=0}^r e^{-\frac{\tau }{\tau (0,q)}}Q_{q}^{kl} = A^k A^l \\&\left( P_0^kP_{0}^le^{-\frac{\tau }{\tau _c}}+2\sum _{q=1}^r y_q^2P_q^kP_{q}^l\cos (q(\phi _k-\phi _l)) e^{-\frac{\tau }{\tau (0,q)}} \right) \\ \end{aligned} \end{aligned}$$

Finally, we consider the case where there is no rotation about the symmetry axis. With axially symmetric global tumbling where the molecular frame has only one arrangement, and where we consider rotations from the PAF directly to the diffusion frame:

B28 $$\begin{aligned} \begin{aligned} C_{kl}^{\textrm{AX,ST}}(\tau )&= A^k A^l \sum _{q_o=0}^r e^{-\frac{\tau }{\tau (q_o,0)}} Q_{q_o}^{\prime kl} \\ \end{aligned} \end{aligned}$$

In this case, as the static molecular frame can be pre-aligned with the diffusion frame this is no requirement to keep the molecular frame and the diffusion frame separate. In the case where $k=l$, the values of $Q_{q_o}^\prime$ reduce to $P_0^2$, $\frac{1}{3}P_1^2$ and $\frac{1}{12}P_2^2$, for $q_o=0,1,2$ respectively as for example stated in Bouguet et al. (2005), and the results depend only on the angle between the positive *z* axis and the diffusion tensor. Finally, in the isotropic static case, as, $\sum _{q=0}^rQ_{q}=P_0\left( \cos \zeta \right)$ we recover Eq. B6.

### Spectral density functions

**Table 12:** Spectral densities for the six motional models implemented in RelCalc expressed as sums over reduced spectral density functions (Eq. 5)

ID	Time-scales	$J^\prime _{(ki),(lj)}(\omega )$		
AX EXT	3$\Lambda$	$\sum _{q_o=0}^2\sum _{\lambda }^\Lambda$	$J(q_o,\lambda ,\omega )$	$\sum _{\beta ,\alpha }^N \frac{n_\lambda }{N^2} A_{\beta }^kA_\alpha ^l\cos$ $\left( \frac{2\pi }{N}\lambda (\beta -\alpha )\right) Q_{q_o}^{\prime kl,\beta \alpha }$
AX ROT	9	$A_kA_l\sum _{q_o=0}^2\sum _{q=0}^2$	$J(q_o,q,\omega )$	$\left( Q_{q_o,q,q}^{kl}+\sum _{q^*=-2}^2\sum _{n=1}^2\delta _{(q-q^*-nN)}Q_{q_o,q,q^*}^{kl}\right)$
AX ST	3	$A_kA_l\sum _{q_o=0}^2$	$J(q_o,0,\omega )$	$Q_{q_o}^{\prime kl}$
ISO EXT	$\Lambda$	$\sum _{\lambda }^\Lambda$	$J(0,\lambda ,\omega )$	$\sum _{\beta ,\alpha }^N\frac{n_\lambda }{N^2} A_{\beta }^kA_\alpha ^l\cos$ $\left( \frac{2\pi }{N}\lambda (\beta -\alpha )\right) P_0\left( \cos \zeta _{kl,\beta \alpha }\right)$
ISO ROT	3	$A_kA_l\sum _{q=0}^2$	$J(0,q,\omega )$	$Q_q^{kl}$
ISO ST	1	$A_kA_l$	$J(0,0,\omega )$	$P_0\left( \cos \zeta _{k,l}\right)$

These cover axially (AX) symmetric and isotropic (ISO) global tumbling, zero local motion (ST), where the interaction strength does not vary because of the local motion (ROT) and the case involving a dipolar interaction to an external spin where the distance in the dipolar interaction is time dependent (EXT). Each model carries a different number of characteristic time-scales, each of which will contribute a separate$J(\tau ,\omega )$ term in the final expression for the final relaxation rates. For the ’EXT’ models, the number of time-scales $\Lambda$ is $\textrm{floor}((N+1)/2)$ where *N* is the number of hopping sites sampled by the motion and the floor function rounds down to the nearest integer. For a methyl group ’hopping’ between sites,$N=3$,$\Lambda =2$. The expressions for $Q_{q_o,q,q^*}$ and $Q_{q}$ are given in Eqs. B14, B21 and B25. In RelCalc, results for ST and ROT are expressed symbolically. In the case of EXT, the spectral density function is held as a constant$C_{ext}(\omega )$and evaluated numerically when required. No matter the motional model, the spectral densities produce results of the form $\sum _{\gamma }\kappa _\gamma J\left( \tau _\gamma ,\omega _{\gamma }\right)$, as anticipated by Eq. 4

The correlation functions for the six motional models (Eqs. B6, B20, B21, B25, B27, B28) are all of the form of Eq. B2, and lead, via Eq. B3 to explicit forms for spectral densities $J^{'}$ (Table 12), and result in relaxation rates of the general form (Eq. 4). These are evaluated internally by RelCalc. For the static and rotating cases (STAT,ROT), where the angles that arrange spins in the molecular frame are specified symbolically, results for relaxation rates are returned in terms of Legendre polynomials $P_q$ (Eq. B18). In the case where axially symmetric global tumbling, results are parameterised by coefficients *M* that rotate the molecular frame into the diffusion frame (Table 11). In the ’external’ case EXT, owing to the complexity of the expressions, relaxation rates are returned keeping the spectral density and characteristic frequency $\omega$, $C_{ext}(\omega )$, and are evaluated numerically.

### Special cases of spectral density functions

We reduce these expressions to forms commonly encountered. In the case of static atoms within an isotropically tumbling molecule (Eq. B7) where $\omega =0$, $J_{ki,lj}^{\prime ,ISO,ST}=A_kA_lP_0(\cos \zeta _{kl}) \tau _c$. When interactions are arranged at the magic angle in the molecular frame, they cannot contribute to relaxation as $P_0(\cos \zeta )=0$. If $k=l$, $\zeta =0$ and $P_0(\cos \zeta )=1$, this reduces simply to $A_k^2\tau _c$.

In the case where all atoms are rotating in the molecular frame, and the molecule is tumbling isotropically, the expression splits into three terms:

B29 $$\begin{aligned} \begin{aligned}&J_{ki,lj}^{\prime ,ISO,ROT} = A_kA_l\\&\left( P_{0}^kP_{0}^l J(0,0,\omega _{ki}) +2\sum _{q=1}^r y_q^2 P_{q}^kP_{q}^l\cos \left( q(\phi _l-\phi _k)\right) J(0,q,\omega _{ki})\right) \\ \end{aligned} \end{aligned}$$

where the spherical harmonic has been expanded into Legendre polynomials to make the angular dependence explicit. In the case of a methyl group in a slowly tumbling molecule undergoing hopping between three sites rather than continuous rotational diffusion $\tau (0,1)=\tau (0,2)=3k$ and so we have only two time-scales to consider. Defining $S^2_{axis}=P_{0}^kP_{0}^l$, then as the time-scales of the $q=1$ and $q=2$ are the same the spectral density reduces to a straightforward and commonly used form Kay et al. (1992):

B30 $$\begin{aligned} \begin{aligned}&J_{ki,lj}^{\prime ,ISO,ROT,AX_3dipolar} = A_kA_l \\&\left( S_{axis}^2 J(0,0,\omega _{ki}) + \left( P_0(\cos \zeta _{k,l})-S_{axis}^2\right) J(0,1,\omega _{ki})\right) \\ \end{aligned} \end{aligned}$$

If we consider an interaction parallel with the CH bond, such as the CH dipolar interaction, $S^2_{axis}$ takes the value $\frac{1}{9}$ where the four bonds are arranged in a perfect tetrahedron. In the case where there are time-dependent interaction strengths, such as where there is a dipolar interaction between the methyl protons and an external proton, and the global tumbling is isotropic and hopping is the mechanism for mass transport around the ring then we have only two time scales to consider, and:

B31 $$\begin{aligned} \begin{aligned} J_{ki,lj}^{\prime ,ISO,EXT,AX_3}&= C_{kl}(0)\left( S^2 J(\tau (0,0),\omega _{ki}) +(1-S^2) J(\tau (0,1),\omega _{ki})\right) \\ \end{aligned} \end{aligned}$$

where

B32 $$\begin{aligned} \begin{aligned} C(0)&=\sum _\alpha ^N\frac{1}{N} A_{\alpha }^kA_{\alpha }^l P_0\cos (\zeta _{kl,\alpha })\\ \end{aligned} \end{aligned}$$

and the order parameter, the ‘proportion’ of the spectral density decayed by global motion:

B33 $$\begin{aligned} \begin{aligned} S^2&=\frac{1}{C(0)}\frac{1}{N^2} \left( \sum _{\alpha ,\beta }^N A_{\alpha }^kA_{\beta }^l P_0(\cos \zeta _{kl,\beta \alpha }) \right) \\ \end{aligned} \end{aligned}$$

This result has been analysed previously for $k=l$Dellwo and Wand (1993), where there are closed form expressions for ensemble averages such as $\langle r_k^{-3}(0)Y_{q}^k(0)\rangle$.

## Appendix C: Time dependent transition probabilities

Local motion in RelCalc is treated by circular motion hopping around the *N* sites about a symmetry axis. We seek to derive the probability of a spin being in site $\beta$ given starting in site $\alpha$ at time $\tau$, $p_{\alpha \beta }(\tau )$ and the characteristic time-scales. Each ’hop’ is characterised by the rate constant $k=\frac{1}{\tau _{HOP}}$. If the ensemble averaged population in each site are given by a vector $\overline{p}$, then the rate equation for their evolution will be $\frac{d\overline{p}}{dt}=R \overline{p}$, where *R* is a square circulant matrix with $-2k$ on each diagonal, *k* on the two adjacent diagonals, and a further *k* in the top right and top left position completing the circular hopping motion. For example, in the case of an $AX_3$ methyl group:

C1 $$\begin{aligned} \frac{d}{d\tau } \left( \begin{array}{c} p_1 \\ p_2 \\ p_3 \\ \end{array} \right) = \left( \begin{array}{ccc} -2k & k & k \\ k & -2k & k \\ k & k & -2k \\ \end{array} \right) \left( \begin{array}{c} p_1 \\ p_2 \\ p_3 \\ \end{array} \right) \end{aligned}$$

The solution to this equation of motion is obtained by taking a matrix exponential, which can be expressed in terms of a matrix of eigenvectors *J* and a diagonal matrix of eigenvalues *D*.

C2 $$\begin{aligned} \begin{aligned} p(\tau )&=\exp \left( R \tau \right) p(0)=J^{-1}\exp \left( -D \tau \right) J p(0) \end{aligned} \end{aligned}$$

There will be *N* eigenvalues and vectors from the circulant matrix, index by $\lambda$ extending from 0 to $N-1$:

C3 $$\begin{aligned} D_\lambda =2k\left( 1-\cos \left( \frac{2\pi \lambda }{N}\right) \right) =\frac{1}{\tau _{loc}(\lambda )} \end{aligned}$$

The corresponding eigenvectors in the square matrix are given by:

C4 $$\begin{aligned} J_{\beta \lambda }=\frac{1}{\sqrt{N}}\exp \left( \frac{2\pi i \lambda \beta }{N}\right) \end{aligned}$$

whose inverse is given by the complex conjugate. If we write the expression for the population in matrix form, the probability of finding a particle at site $\beta$:

C5 $$\begin{aligned} p_\beta (\tau )=J_{\beta \lambda }e^{-D_{\lambda }\tau }J^{-1}_{\lambda \alpha }P_{\alpha }(0) \end{aligned}$$

where $P_\alpha (0)$ gives the populations of sites where the particle started. If we impose the initial condition that the particle starts on site $\alpha$, then the conditional probability of ending on $\beta$ given starting on $\alpha$ at time *t* becomes

C6 $$\begin{aligned} p_{\beta \alpha }(\tau )=\sum _{\lambda =0}^{N-1}\exp \left( -\frac{\tau }{\tau _{loc}(\lambda )}+i\frac{2\pi }{N}\lambda (\beta -\alpha ) \right) \end{aligned}$$

There is a natural degeneracy in the eigenvalues (Fig. 9b) that leads to $\tau _{loc}(\lambda )=\tau _{loc}(N-\lambda )$ and there being $\Lambda =N/2+1$ unique frequencies if *N* is even, and $\Lambda =(N+1)/2$ otherwise. When $\lambda =0$, and if *N* is even, also when $\lambda =N/2$, the time-scale will be unique ($n_\lambda =1$), but doubly degenerate otherwise ($n_\lambda =2$), giving the result for the time dependent transition probabilities, the condition probability for arriving in site $\beta$ having started in site $\alpha$, in a given unit of time $\tau$.

C7 $$\begin{aligned} p_{\beta \alpha }(\tau )=\sum _{\lambda =0}^{\Lambda }n_\lambda \cos \left( \frac{2\pi }{N}\lambda (\beta -\alpha )\right) \exp \left( -\frac{\tau }{\tau _{loc}(\lambda )} \right) \end{aligned}$$

At $\tau =0$, $p_{\beta ,\alpha }=0$ unless $\beta =\alpha$, and as $\tau$ moves to infinity, the probability becomes uniform where the probability of finding a spin in site $\beta$ is independent of where it started. The factor $\exp \left( -\frac{\tau }{\tau _{loc}(\lambda )} \right)$ has included in the generic time-scale (Eq. 7). When discussing a ‘hopping’ model we define $\tau _m=\tau _{\textrm{Hop}}=\frac{1}{Nk}$.

Finally, we obtain the free diffusion limit by taking Eq. C3 to the $N\rightarrow \infty$ limit, and $\frac{1}{\tau _{loc}(\lambda )}= 2k\left( \frac{2\pi }{N}\right) ^2\lambda ^2= D \lambda ^2$. Noting that $2k\left( \frac{2\pi }{N}\right) ^2=D=\frac{2}{\tau _{\textrm{Diff}}}$ has the units of a diffusion coefficient, change in squared angle per unit time, we have recovered the free diffusion limit (Eq. B16). When this is used in computations we set $\tau _m=\tau _{\textrm{Diff}}$. For $N>4$ this is equivalent to performing the following motional average when evaluating local motion models

C8 $$\begin{aligned} \langle D_{qq}(\tau )D_{pp}(0)\rangle =\delta _{q,p} \exp \left( -\tau D q^2\right) \end{aligned}$$

## Appendix D: Numerical evaluation correlation function with variable distances

In RelCalc, the correlation functions are numerically evaluated in the ’EXT’ case, where either of the interactions *k* or *l* are dipolar, and the distance varies depending on the local motion, such as where a proton in a methyl group is relaxed by an external spin not participating in the same local motion. In this case, where global tumbling is either anisotropic (AX) or isotropic (ISO), to calculate the required values of *Q*, we need to evaluate the angles $\theta$ and $\phi$ that characterise the interactions *k* and *l* at all points in the motion.

To do this, we explicitly calculate the vector between the spin undergoing local motion (*E*) located at $(r_E,\theta _E,\phi _E+\frac{2\pi }{N}b)$, where the integer *b* tracks the rotation of the spin moving around a circle in the *xy* plane, and a spin that is static in the molecular frame (*D*), located at $(r_D,\theta _D,\phi _D)$. The vector between the two in a Cartesian basis will be:

D1 $$\begin{aligned} \begin{aligned} x&= r_E\sin \theta _E\cos \left( \phi _E+\frac{2\pi }{N}b\right) -r_D\sin \theta _D\cos \phi _D\\ y&= r_E\sin \theta _E\sin \left( \phi _E+\frac{2\pi }{N}b\right) -r_D\sin \theta _D\sin \phi _D\\ z&= r_E\cos \theta _E-r_D\cos \theta _D\\ \end{aligned} \end{aligned}$$

Whose magnitude, the distance between the spins is:

D2 $$\begin{aligned} \begin{aligned} A&=r_E^2 + r_D^2 - 2r_E r_D \left( \cos \theta _E\cos \theta _D\right) \\ B&=2r_E r_D \sin \theta _E\sin \theta _D\cos (\phi _E-\phi _D)\\ C&=2r_E r_D \sin \theta _E\sin \theta _D\sin (\phi _E-\phi _D)\\ r_{ED}&=A-B\cos \left( \frac{2\pi }{N}b\right) +C\sin \left( \frac{2\pi }{N}b\right) \\ \end{aligned} \end{aligned}$$

In spherical polar co-ordinates, this vector will be $\overline{V}_{ext}=(r_{ED},\theta ,\phi )$ where $\cos \theta =\frac{z}{r_{ED}}$, and $\tan 2\phi =\frac{x}{y}$. With $\cos \theta$ and $\phi$ for all sites, *Q* can be calculated for each position in the motion, allowing $J^\prime$ to be calculated. Where *k* or *l* are dipolar, $A_k$ or $A_l$ will be $1/r_{ED}^3$. The spherical vectors in the molecular frame can be then combined with rotations into the diffusion frame to account for anisotropic diffusion.

## Appendix E: Mapping successive rotations

In multiple instances when computing relaxation rates, the sums over multiple rotations can be simplified by replacing successive rotations with a single one. This can be important for orienting an interaction in the molecular frame as well as simplifying results. The effects of this are straight forward to evaluate using rank 1 Wigner rotations and spherical Harmonics, where a spherical Harmonic describes rotation from a PAF into a molecular frame by the angles $\theta _O$ and $\phi _O$, following by a second rotation, $\theta _R,\phi _R$.

E1 $$\begin{aligned} \sum _{q=-1}^1 D_{p,q}^{(1)}(\theta _R,\phi _R)Y_{q}(\theta _O,\phi _O)= Y^\prime _{p}(\theta ^\prime ,\phi ^\prime ) \end{aligned}$$

The result is a new spherical harmonic characterised by the angles $\theta ^\prime ,\phi ^\prime$.

E2 $$\begin{aligned} \begin{aligned}&\theta ^\prime = \arccos \left( \cos \theta _O \cos \theta _R-\sin \theta _O \cos \phi _O \sin \theta _R\right) \\&\phi ^\prime = \arctan _2\\&\left( \begin{array}{ll}\sin \theta _O \left( \cos \phi _O \cos \theta _R \cos \phi _R- \sin \phi _O \sin \phi _R\right) +\cos \theta _O \sin \theta _R \cos \phi _R,\\ \sin \phi _R \left( \sin \theta _O \cos \phi _O \cos \theta _R+\cos \theta _O \sin \theta _R\right) +\sin \theta _O \sin \phi _O \cos \phi _R\end{array} \right) \end{aligned} \end{aligned}$$

The $\arctan _2(x,y)$ function follows the mathematica definition where $x=\cos \phi ^\prime$ and the second is $y=\sin \phi ^\prime$.

## Appendix F: Spherical tensor definitions

Owing to non-standardised and in some places conflicting definitions for the methods to map spherical and Cartesian tensors, for completeness, the relations used in RelCalc are explicitly stated below which can be tested for consistency (using e.g. mathematica or similar). By first defining the SO(3) rotation generators

F1 $$\begin{aligned} \begin{array}{rlrlrl} M_x & = \left( \begin{array}{ccc}0& 0& 0\\ 0& 0& -1\\ 0& 1& 0 \end{array} \right) , & M_y & = \left( \begin{array}{ccc}0& 0& 1\\ 0& 0& 0\\ {-}1& 0& 0 \end{array} \right) , & M_z & = \left( \begin{array}{ccc}0& -1& 0\\ 1& 0& 0\\ 0& 0& 0 \end{array} \right) \end{array} \end{aligned}$$

which satisfy the commutation relation $[M_x,M_y]=M_z$ and its cyclic permutations. We can then define the normalised irreducible basis matrices

F2 $$\begin{aligned} \begin{array}{rlrl} & & M_{\pm 2}^{(2)}& = \frac{1}{2}\left( M_x^2-M_y^2 \pm i [M_y^2,M_z]\right) \\ M_{\pm 1}^{(1)}& =\frac{1}{2}\left( -M_y \pm i M_x\right) & M_{\pm 1}^{(2)}& = \frac{1}{2}\left( \pm [M_z^2,M_y ] -i[M_x,M_y^2]\right) \\ M_0^{(1)}& =-\frac{i}{\sqrt{2}}M_z & M_0^{(2)}& = \frac{1}{\sqrt{6}}\left( 2 M_z^2 -M_x^2 -M_y^2\right) \end{array} \end{aligned}$$

Which explicitly are

F3 $$\begin{aligned} \begin{array}{rlrlrl} & & & & M_{\pm 2}^{(2)} & = \frac{1}{2}\left( \begin{array}{ccc} 1& \pm i& 0\\ \pm i& -1& 0\\ 0& 0& 0\\ \end{array}\right) \\ & & M_{\pm 1}^{(1)} & = \frac{1}{2}\left( \begin{array}{ccc} 0& 0& - 1\\ 0& 0& \mp i\\ 1& \pm i& 0\\ \end{array}\right) & M_{\pm 1}^{(2)} & = -\frac{1}{2}\left( \begin{array}{ccc} 0& 0& 1\\ 0& 0& \pm i\\ 1& \pm i& 0\\ \end{array}\right) \\ M_{0}^{(0)} & = \frac{1}{\sqrt{3}} \left( \begin{array}{ccc} 1& 0& 0\\ 0& 1& 0\\ 0& 0& 1\\ \end{array}\right) & M_{0}^{(1)} & = \frac{i}{\sqrt{2}}\left( \begin{array}{ccc} 0& 1& 0\\ -1& 0& 0\\ 0& 0& 0\\ \end{array}\right) & M_{0}^{(2)} & = \frac{1}{\sqrt{6}}\left( \begin{array}{ccc} -1& 0& 0\\ 0& -1& 0\\ 0& 0& 2\\ \end{array}\right) \\ \end{array} \end{aligned}$$

These have symmetries $M_q^{(r)\dag } = (-1)^q M_{-q}^{(r)}$, $M_q^{(r)*} = (-1)^{k-q} M_{-q}^{(r)}$, $(M_q^{(r)})^T = (-1)^{k} M_{q}^{(r)}$, and are orthonormal ($\textrm{Tr}\left( M_q^{(r)\dag } M_p^{(k)}\right) =\delta _{r,k}\delta _{p,q}$). The Hamiltonian is given by $\mathcal {H}=\textrm{Tr}\left( A. T\right)$ where $T=S\otimes I$ has elements of spin operators, $\hat{T}_{ij}=\hat{I}_j\hat{S}_i$, where $i,j=xyz$ (the first index goes with the *S* spin and the second index goes with the *I* spin). The Cartesian representations two tensors are

F4 $$\begin{aligned} \begin{array}{lr} A=\left( \begin{array}{ccc} A_{xx} & A_{xy} & A_{xz} \\ A_{yx} & A_{yy} & A_{yz} \\ A_{zx} & A_{zy} & A_{zz} \\ \end{array}\right) & , \\ T=\left( \begin{array}{ccc} \hat{T}_{xx} & \hat{T}_{xy} & \hat{T}_{xz} \\ \hat{T}_{yx} & \hat{T}_{yy} & \hat{T}_{yz} \\ \hat{T}_{zx} & \hat{T}_{zy} & \hat{T}_{zz} \\ \end{array}\right) \\ \end{array} \end{aligned}$$

and the Hamiltonian is $\mathcal {H}=\sum _{i,j}A_{ij}\hat{T}_{ji}=\sum _{i,j=x,y,z} \hat{I}_i A_{ij} \hat{S}_j$. We can project both *A* and *T* into the spherical tensor basis using $B^{(r)}_p=\textrm{Tr}\left( M^{(r)\dag }_p B\right)$ (where *B* can be either *A* or *T*) to yield:

F5 $$\begin{aligned} & B_{ \pm 2}^{(2)} = \frac{1}{2}\left( B_{xx}- B_{yy} \mp i\left( B_{xy} + B_{yx}\right) \right) \nonumber \\ & B_{ \pm 1}^{(1)} = \frac{1}{2} \left( B_{zx} - B_{xz} \pm i \left( B_{yz} - B_{zy}\right) \right) B_{ \pm 1}^{(2)} \nonumber \\ & = \frac{1}{2} \left( \mp \left( B_{zx} + B_{xz}\right) + i \left( B_{zy} + B_{yz}\right) \right) \nonumber \\ & B_{0}^{(0)} = \frac{1}{\sqrt{3}} (B_{xx}+B_{yy}+B_{zz}) B_{0}^{(1)} = \frac{i}{\sqrt{2}}\left( B_{yx}- B_{xy}\right) B_{0}^{(2)} \nonumber \\ & = \frac{1}{\sqrt{6}}\left( 2 B_{zz} -B_{xx} - B_{yy} \right) \end{aligned}$$

These have the symmetry $$B_q^{(r)*} = (-1)^{k-q} B_{-q}^{(r)}$$. When applying this to *T*, we can further make the substitutions $$\hat{I}_x=\frac{1}{2}(\hat{I}_++\hat{I}_-)$$ and $$\hat{I}_y=\frac{i}{2}(\hat{I}_--\hat{I}_+)$$ for both *I* and *S* spins to obtain the spherical tensor operators written in the Zeeman basis (Table 8). The Hamiltonian in the spherical tensor basis is $$\mathcal {H}=\sum _{r=0}^2\sum _{p=-r}^r (-1)^{p} A^{(r)}_p \hat{T}_{-p}^{(r)}$$$$= \sum _{r=0}^2\sum _{p=-r}^r (-1)^{r} A^{(r)}_p \hat{T}_{p}^{(r)*}$$.

For completeness, the relations that enable returning from the spherical basis back to the Cartesian are computed. We require that *A* and *T* are returned to the Cartesian basis in a manner that restores the Hamiltonian to the state prior to the transform:

F6 $$\begin{aligned} \mathcal {H} = \sum _{k,p} (-1)^p A_p^{(k)} \hat{T}_{-p}^{(k)} = \sum _{ab}A_{ab}\hat{T}_{ba} \end{aligned}$$

Noting that the coefficients of the spherical basis $A_p^{(k)} = \textrm{Tr}(M_p^{(k)\dag } \sum _{ab}A_{ab}C_{ab})$, where $C_{ab}$ are the 9 real single element basis matrices that define the Cartesian basis and so:

F7 $$\begin{aligned} \begin{array}{rl} H & =\sum _{abcd} A_{ab} \hat{T}_{cd} \sum _{k,p} \textrm{Tr}(M_p^{(k)\dag }C_{ab}) \textrm{Tr}((-1)^p M_{-p}^{(k)\dag } C_{cd}) \\ & =\sum _{abcd} A_{ab} \hat{T}_{cd} \sum _{k,p} \textrm{Tr}(M_p^{(k)\dag }C_{ab}) \textrm{Tr}( M_{p}^{(k)} C_{cd}) \end{array} \end{aligned}$$

The sum of the product of traces is zero unless $C_{cd}^\dag = C_{ab}$, which is achieved if $\delta _{c,b}$ and $\delta _{a,d}$ and so:

F8 $$\begin{aligned} \begin{array}{rl} H&=\sum _{ab} A_{ab} \hat{T}_{ba} \sum _{k,p} \textrm{Tr}(M_p^{(k)\dag }C_{ab}) \textrm{Tr}( M_{p}^{(k)} C_{ba}^\dag ) \\ & \quad = \sum _{ab} A_{ab}\hat{T}_{ba} \end{array} \end{aligned}$$

as required. This implies that we can map the spatial part onto the Cartesian basis matrices using the commonly encountered projection operator, $$A_{ab}= \sum _{kp}A_p^{(k)}$$ $$ \textrm{Tr}(C_{ab}^\dag M_p^{(k)})$$ . The basis tensors however require an additional transpose and so $$\hat{T}_{cd}= \sum _{kp}\hat{T}_p^{(k)} $$ $$\textrm{Tr}(C_{dc}^{\dag } M_p^{(k)})$$ , which we can express as an ‘adjusted’ projection operator, $$\hat{T}_{cd}= \sum _{kp}\hat{T}_p^{(k)} $$ $$\textrm{Tr}(C_{cd}^{*} M_p^{(k)})$$ . Noting that $$(M_p^{k})^T $$ $$= (-1)^k $$ $$M_p^{(k)}$$ then $$\hat{T}_{cd}= \sum _{kp}\hat{T}_p^{(k)} $$ $$\textrm{Tr}(C_{cd}^{\dag } (-1)^k $$ $$M_p^{(k)} )$$ , which reveals using the normal projection operator for the tensors will invert the sign of the rank 1 components of the tensor in the Hamiltonian, amounting to a transpose. Using the more typical projection operator for the spatial part and the adjusted one for the tensors allow the elements of the Cartesian tensors to be reconstructed using the following (where *B* can be either *A* or *T*),

F9 $$\begin{aligned} \begin{array}{rl} B_{xx} & = \frac{1}{\sqrt{3}}B_0^{(0)} - \frac{1}{\sqrt{6}}B_0^{(2)} +\frac{1}{2} \left( B_{-2}^{(2)}+B_{2}^{(2)}\right) \\ B_{yy} & = \frac{1}{\sqrt{3}}B_{0}^{(0)}-\frac{1}{\sqrt{6}}B_{0}^{(2)} -\frac{1}{2}\left( B_{-2}^{(2)}+ B_{2}^{(2)}\right) \\ B_{zz} & = \frac{1}{\sqrt{3}}B_{0}^{(0)}+\sqrt{\frac{2}{3}} B_{0}^{(2)} \\ B_{xy} & = \frac{i}{\sqrt{2}} B_{0}^{(1)}+ \frac{1}{2}i ( B_{2}^{(2)} - B_{-2}^{(2)} ) \\ B_{yx} & = -\frac{i }{\sqrt{2}}B_{0}^{(1)} +\frac{1}{2}i\left( B_{2}^{(2)} - B_{-2}^{(2)}\right) \\ B_{xz} & = \frac{1}{2} \left( -B_{-1}^{(1)}-B_{1}^{(1)}+B_{-1}^{(2)}- B_{1}^{(2)}\right) \\ B_{zx} & = \frac{1}{2}\left( B_{-1}^{(1)}+B_{1}^{(1)}+B_{-1}^{(2)}-B_{1}^{(2)}\right) \\ B_{yz} & = \frac{1}{2}i\left( B_{-1}^{(1)}- B_{1}^{(1)}- B_{-1}^{(2)}- B_{1}^{(2)}\right) \\ B_{zy} & = \frac{1}{2}i\left( - B_{-1}^{(1)}+ B_{1}^{(1)}- B_{-1}^{(2)}- B_{1}^{(2)}\right) \end{array} \end{aligned}$$

From these identities it is straightforward to prove Eq. F6 and results in either a Cartesian or spherical basis can be mapped back and forth in a self-consistent manner (Mueller (2011); Pascal (2013)). In the more common case where the interaction tensor is symmetric, then the naive projection operator can applied to map the spherical basis back to the Cartesian and additional complexity due to the rank 1 tensor does not arise.

### Active and passive rotations

Active and passive rotations are accomplished using Wigner matrix elements as follows. We prove their equality and demonstrate that equally rotating both basis and spatial tensor leaves the Hamiltonian unchanged. An active rotation of each element of the spatial tensor is

F10 $$\begin{aligned} R A_q^{(r)}M_q^{(r)} R^{-1} = \sum _{p=-r}^r D_{q,p}^{(r)}A_p^{(r)}M_p^{(r)} \end{aligned}$$

and the equivalent passive rotation of the basis is achieved by

F11 $$\begin{aligned} \begin{array}{rl} R^{-1} \hat{T}_{q}^{(r)}M_{q}^{(r)} R&= \sum _{p=-r}^r D_{q,p}^{\prime (r)} \hat{T}_{p}^{(r)}M_p^{(r)} . \end{array} \end{aligned}$$

where the order of the axis of rotations and the sign of the angles are reversed relative to the active case, indicated by the prime$^\prime$. In the active case, the rotated Hamiltonian is computed from pairing spatial tensors in the new frame with basis tensors in the original ($\hat{T}_{q}^{(r)*}$),

F12 $$\begin{aligned} \mathcal {H}^\prime = \textrm{Tr}( R A R^{-1} T) = \sum _{r=0}^2 \sum _{qp=-r}^r (-1)^r D_{q,p}^{(r)}A_p^{(r)} \hat{T}_{q}^{(r)*} \end{aligned}$$

and in the passive rotation the Hamiltonian is computed from pairing spatial tensors in the original frame ($A_q^{(r)}$) with the transformed basis tensors

F13 $$\begin{aligned} \begin{array}{rl} \mathcal {H}^\prime & = \textrm{Tr}( A R^{-1} T R) = \sum _{r=0}^2 \sum _{qp=-r}^r (-1)^{q} A_q^{(r)} D_{-q,-p}^{\prime (r) }\hat{T}_{-p}^{(r)} \\ & = \sum _{r=0}^2 \sum _{qp=-r}^r (-1)^{q} A_q^{(r)} (-1)^{p-q} D_{p,q}^{(r) }\hat{T}_{-p}^{(r)} \\ & = \sum _{r=0}^2 \sum _{qp=-r}^r (-1)^{r} A_q^{(r)} D_{p,q}^{(r) }\hat{T}_{p}^{(r)*} \\ \end{array} \end{aligned}$$

where we note that $D_{p,q}^\prime (-\gamma ,-\beta ,-\alpha )=(-1)^{q-p}D_{-q,-p}(\alpha ,\beta ,\gamma )$. The results are identical confirming passive and active rotations result in the same Hamiltonian. Finally, if we rotate both spatial and basis tensors identically then

F14 $$\begin{aligned} \begin{array}{rl} \mathcal {H}^{\prime \prime } & = \textrm{Tr}( R A R^{-1} RT R^{-1}) = \sum _{r=0}^2 \sum _{qpp^\prime =-r}^r (-1)^q D_{q,p}^{(r)}A_p^{(r)} D_{-q,-p^\prime }^{\prime (r)}\hat{T}_{-p^\prime }^{(r)} \\ & = \sum _{r=0}^2 \sum _{pp^\prime =-r}^r (-1)^{p^\prime } A_p^{(r)} \hat{T}_{-p^\prime }^{(r)} \sum _{q=-r}^r D_{q,p}^{(r)} D_{q,p^\prime }^{(r)*} \\ & = \sum _{r=0}^2 \sum _{p=-r}^r (-1)^p A_p^{(r)} \hat{T}_{-p}^{(r)} =\mathcal {H} \\ \end{array} \end{aligned}$$

and the Hamiltonian is unchanged, as expected. The orthonormality condition $\sum _q D_{q,p}^{(r)} D_{q,p^\prime }^{(k)*} = \delta _{r,k}\delta _{p,p^\prime }$ and $D_{p,q}^{(r)}=D_{q,p}^{(r)*}$ were used.

## Appendix G: Symbolic matrix algebra

BWR calculations require large numbers of selection factors $S_{ki,lj}^{st}$ to be evaluated (equation 11). These require evaluation of commutator ($[\hat{O},\rho ]=\hat{O}\rho - \rho \hat{O}$) and trace ($\langle A|B \rangle$) operations. Such computations in RelCalc occur in three stages

G1 $$\begin{aligned} \begin{array}{rl} A & =\left[ \hat{O}_{ki}^\dagger ,\rho _t\right] \\ B& = \left[ \hat{O}_{lj},A \right] \\ S_{ki,lj}^{st}& = \langle \rho _s| B \rangle \end{array} \end{aligned}$$

After $\mathcal {H^\textrm{1}}$ is defined, elements are sorted and grouped first by $O_{ki}$ and then by $O_{lj}$. Evaluation of $S_{kl,lk}^{st}$ is aborted if either *A* or *B* is zero. By saving the result of *B*, further efficiency is achieved by retaining *B* and looping over all required $\rho _t$. This is the case whether using symbolic or matrix descriptions of the operators.

For systems of spins $\frac{1}{2}$, the commutation and trace operations can be performed symbolically removing the need to construct matrix representations. For the commutators, as the operators for the interactions $\hat{O}$ are linear, quadratic or bi-linear, there will be (up to) two elements for spins equal to either $z,+,-$ (Table 9), with the element corresponding to any other spins in the system being described by the identity. This allows evaluation of the commutator be restricted to only the subspace containing the (up to) two elements, as all other elements will not be affected by the commutator. For spin halves, where the operators for each spin in $\rho$ take the value x,y,z,E,+,-,$\alpha$ or $\beta$, when combined with the allowed operators for $\hat{O}$, there are exactly 660 non-zero commutators. These combinations and result are stored in a symbolic library in RelCalc. To evaluate the commutator, we take the elements of $\hat{O}$, find the operators that ‘line up’ from the operator on the right of the expression, evaluate the double commutator using the look-up table and copy up the remaining values. For example, to evaluate $[C^1_zH^2_z,H^2_xH^3_z]$ we note that $H^3_z$ is a spectator, and the commutator to evaluate is $[zz,xE]=yz$. The result then is then $C^1_zH^2_yH^4_z$.

Similar logic could be applied to evaluation of the double commutator, *B*. Brute force computation reveals that there are 88,560 non-zero double commutators for spin halves that involve a maximum of 4 spins. In practice time taken for typical calculations was orders of magnitude longer when using a library of double commutators, than when using the library of single commutators (where computation can be halted at *A* if it returns zero).

Finally, the trace of a matrix can be written as a product of single element traces where the operators *S* and *T* are outer products of individual elements then

G2 $$\begin{aligned} {<}S |T> =\prod _i^N <\hat{S}_i\hat{T}_i>. \end{aligned}$$

For the set of basis operators described above, there are 20 pairs of operators that give non-zero results for single spin evaluations

G3 $$\begin{aligned} \begin{array}{cccccc} \langle E|E\rangle =2 & \langle \beta |\beta \rangle =1 & \langle E|\alpha \rangle =1 & \langle z|\beta \rangle =-\frac{1}{2} & \langle -|x\rangle =\frac{1}{2} & \langle +|y\rangle = -\frac{i}{2}\\ \langle x|x\rangle =\frac{1}{2} & \langle +|+\rangle =1 & \langle E|\beta \rangle =1 & \langle \beta |z\rangle =-\frac{1}{2} & \langle x|-\rangle =\frac{1}{2} & \langle y|+\rangle = \frac{i}{2}\\ \langle y|y\rangle =\frac{1}{2} & \langle \alpha |\alpha \rangle =1 & \langle \beta |E\rangle =1 & \langle z|\alpha \rangle =\frac{1}{2} & \langle +|x\rangle =\frac{1}{2} & \langle -|y\rangle = \frac{i}{2}\\ \langle z|z\rangle =\frac{1}{2} & \langle -|-\rangle =1 & \langle \alpha |E\rangle =1 & \langle \alpha |z\rangle =\frac{1}{2} & \langle x|+\rangle =\frac{1}{2} & \langle y|-\rangle = -\frac{i}{2}\\ \end{array} \end{aligned}$$

These combinations are retained in memory and used to evaluate equation G2 by working through each product sequentially, returning zero the moment any individual element in the product is found to be zero.

As the operators for the trace and commutators in the libraries are restricted to single spin results, the situation becomes more challenging when the operator is expressed as a linear combination. RelCalc allows for this, where an operator for $\rho _s$ or $\rho _t$ can be stored as a list of operator strings, each linked to a number. For example, in the irreducible basis the $J=1$, $m_j=0$ population operator for an $X_2$ spin system will be $\frac{1}{2}\alpha \beta +\beta \alpha> <\alpha \beta +\beta \alpha$, which when expressed in terms of basis operators requires 4 terms $\frac{1}{2}\left( \hat{X}_\alpha \hat{X}_\beta +\hat{X}_\beta \hat{X}\alpha + \hat{X}_-\hat{X}_+ + \hat{X}_+\hat{X}_-\right)$. A symbolic treatment requires each element of the list to be treated independently, with results from each evaluation being combined at the end of the computation. When considering the irreducible representations of the Platonic $AX_n$, the number of individual operators can get very large. As a matrix representation does not have to consider individual operators separately, there is a point where matrices become more efficient. If, for example, computing a complete Liouvillian matrix for a spin system using a shift basis, each basis operator will have a single term, and so symbolic computation of rates will always be more efficient, increasingly so the larger the spin system gets.

The symbolic mode is optional. Where spins > $\frac{1}{2}$ are required, either full or sparse matrix representations of the operators are required, where the Cartesian and shift operators will be automatically constructed. A similar approach to the above for spins $>\frac{1}{2}$ could be implemented.

## Appendix H: Basis choice

Relaxation rates $R_{st}$ link basis operators $\rho _s$ and $\rho _t$. The choices of these, as discussed in the text, will depend on the requirements. It is often desirable to work with operators constructed from wave-functions that span the eigenbasis of $\mathcal {H}^0$. When analysing a spin system with distinguishable spins with weak coupling only, the eigenbasis of the combined system can be constructed from outer products of the single spin eigenfunctions. For spin halves, taking outer products of the +, −, $\alpha$ and $\beta$ elements for each spin will form a complete shift basis, where each operator is an eigenfunction of combined spin Hamiltonian. In the case where there are degenerate spins, this is no longer the case. RelCalc provides two methods for dealing with these situations.

### Single quantum Zeeman basis

When analysing the effect of, for example, a $90^o$ *A* pulse on an $AX_n$ spin system (setting the density matrix to $A_+$), the resulting spectrum is well described by the single quantum zeeman basis. The density matrix is then given by $\hat{A}_+ \equiv \hat{A}_+ \Pi _{i=1}^n\otimes \hat{X}_E^i$. For each nucleus of spin *S*, the identity $X_E$ can be described by a sum over the $2S+1$ single element population matrices corresponding to the possible angular momentum operators $\hat{X}_E^i=\sum _{m=-S}^{S} \hat{X}_{m}^i$, where each single element operator $\hat{X}_m^i$ is a matrix with 1 on the relevant diagonal, and zero elsewhere (for a spin $\frac{1}{2}$, $X_E=X_\alpha +X_\beta$). For *n* coupled spins,

H1 $$\begin{aligned} \hat{A}_+ = \hat{A}_+ \Pi _{i=1}^n\otimes \left( \sum _{m=-S}^{S} \hat{X}_{m}^i\right) . \end{aligned}$$

Expanding the outer product will yield a total of $(2S+1)^n$ individual operators with $A_+$ and then all permutations and combinations of $X_m$ for the each of the *X* spins. These can be grouped into $2nS+1$ sets that each evolve with a unique frequency given by the common projection of the angular momentum. This is the basis for ‘tree’ diagrams used to predict coupling patterns in chemical NMR, which reduces to Pascal’s triangle where *X* is spin $\frac{1}{2}$. A function is provided to return this basis either in matrix form, or symbolically as sums of individual single spin operators. This basis is suitable for describing the *A* spin where the *X* spin state can be described purely by populations (no coherences).

### Irreducible basis

A complete basis for a degenerate $X_n$ spin system will require $(2S+1)^N$ wave-functions. In this case, the Hamiltonian contains the total angular momentum operator, and so we seek basis wavefunctions that form an eigenbasis of this operator. The total angular momentum operator $\hat{J}^2= \sum _{ij}\hat{J}_i\hat{J}_j$Corio (1966). This can be further expanded

H2 $$\begin{aligned} & \hat{J}^2= \sum _{i=1}^N \hat{J}_i^2 + \sum _{j=i+1}^N 2 \hat{J_i}\cdot \hat{J_j} =\sum _i \hat{J}_i^2 \nonumber \\ & + \sum _{j=i+1}^N 2\hat{J}_{iz}\hat{J}_{jz} + \hat{J}_{+i}\hat{J}_{-j} + \hat{J}_{-i}\hat{J}_{+j}. \end{aligned}$$

The operators $\hat{J}_{+i}\hat{J}_{-j} + \hat{J}_{-i}\hat{J}_{+j}$ imply that the action of the total angular momentum operator on a single spin wave-function is to cause pairwise permutations of the form $\alpha \beta \rightarrow \beta \alpha$, and its reverse. The eigenbasis for a given projected angular momentum will necessarily span a range of permutations. The set of ortho-normal wavefunctions for spin halves that form the eigenbasis of $\mathcal {H}^0$ is often termed the irreducible basis are computed as described below and numbered in RelCalc from 1 to $2^N$. The $(2S+1)^{2N}=4^N$ Liouvillian basis operators of the form $i\rangle \langle j$ can then be constructed to form a complete basis to describe spin dynamics that can be interpreted in terms of an ‘energy level’ picture. We next describe how using group theoretical methods we can assemble the irreducible wavefunctions, and then how these are combined to make a complete basis in RelCalc.

Under the total angular momentum (strong J coupling) Hamiltonian, we can obtain eigenfunctions following the rules for addition of quantum mechanical sources of angular momentum (Corio 1966). These will be irreducible wavefunctions $\Phi _{J,m_J}$Corio (1966), described by linear combinations of single spin wave-functions $\psi _{l,m_l}^i$ that will collect into manifolds with total angular momentum *J*, each with $2J+1$ members with a projected angular momentum value in the range, $m_J=-J,-J+1,...,J$. For spin halves, RelCalc uses group theory to compute the wave-functions that span the irreducible representation (Corio 1966). First, the $2^n$ single spin wave-functions with all combinations of $\alpha$ and $\beta$ states for the *n* spins are assembled and placed into $N+1$ groups each containing $N!/N_\alpha !N_\beta !$ single spin wave-functions where $N_\alpha$ and $N_\beta$ are the total number of $\alpha$ and $\beta$ elements in the group. The groups are all eigenfunctions of the projected angular momentum operator, sharing the same value of $j_z = \langle \psi | \hat{J}_z \psi \rangle =\frac{1}{2}\left( N_\alpha -N_\beta \right)$.

A point group is assigned to the spin system as a whole, and the matrix representations $\hat{T}_h$ are calculated for each of its *h* symmetry operations that permute the positions. A reducible representation is constructed by taking each wave-function in the group in turn, applying all symmetry operations to it and then counting the number of spins that are unchanged for this operation, creating a vector $P_h$. The number of times each irreducible representation *r* spans the group is given by $C_r=\frac{1}{h}\sum _h \chi _{h,r} P_h$, where $\chi _{h,r}$ is the character for the irreducible representation *r* for symmetry operation *h*. The final task is to represent the irreducible wave-functions in terms of linear combinations of the original single spin wave-functions. This is accomplished by calculating symmetry adjusted wavefunctions from the projection formula, $\phi _i=\sum _i\sum _h\chi _hT_h\psi _i$. The resulting wave-functions are not intrinsically ortho-normal, and so a Gramm-Schmidt procedure is iteratively applied to construct suitable linear combinations that are orthonormal:

H3 $$\begin{aligned} \Phi _i = \phi _i - \sum _{i}^j\frac{\langle \phi _j|\phi _i\rangle }{\langle \phi _j|\phi _j\rangle } \phi _j \end{aligned}$$

resulting in a final set of wave-functions $\Phi _i$ from the set of symmetry adjusted functions $\phi _i$. While these functions are eigenvectors of the total angular momentum operator, the above procedure alone is not guaranteed to generate wave-functions connected within a manifold by the raising and lowering operators. To accomplish this, we first set our target angular momentum $j_{z,target}=j_{z,max}=N/2$ which will be a single degenerate state described by an outer product of all single spin wavefunctions taking their maximum value. This state will be a member of the symmetric representation. The lowering operator is then used to create new wave-functions, members of the same *J* manifold, until $j_z=-j_{max}$ is reached. $j_{target}$ is then reduced by 1. The irreducible wave-functions for this state are obtained from the projection operator as described above. If the irreducible representation is singly degenerate, the lowering operator is applied, and the manifold is assembled as for the symmetric representation. If the representation is degenerate, the first irreducible representation is taken, and the rest are found via gram-schmidt othogonalisation. The resulting wavefunctions are then exposed to the lowering operator, and checked for orthogonality. This repeats until $j_{target}$ is less than zero, in which case $2^N$ symmetry adapted orthonormal irreducible wavefunctions $\Phi$ will have been found.

The advantage of using point groups rather than straight application of the permutation operator to compute these is that the results tend to form linear combinations in terms of rational numbers, which is numerically pleasing. The wavefunctions are numbered 1 to $2^N$ in the above order, normalised, with the symmetry label of the irreducible representation together with total and projected angular momenta of each wavefunction being retained. The explicit forms are provided in the report for reference. Examples are presented for $AX_n$ in a range of symmetries including the platonic solids (Fig. 7).

Taking two spin halfs, $X_2$, as an example, the irreducible representation will require $2^2=4$ wavefunctions. The procedure above yields two manifolds, one with $J=1$ (a triplet with wavefunctions $m_j=1,0,-1$) and one with $J=0$ (a singlet with a wavefunction $J=0$ and $m_j=0$) (Fig. 2A). For example, the singlet state for $X_2$ returned by RelCalc has the wave-function

H4 $$\begin{aligned} \left|4\right>=\frac{1}{\sqrt{2}}\left( \left|\psi _{\alpha }^1\psi _{\beta }^2\right>-\left|\psi _{\beta }^1\psi _{\alpha }^2\right>\right) \end{aligned}$$

Having computed an irreducible basis of wavefunctions of degenerate spins, the final task is to combine these via outer products to form $4^N$ Liouvillian basis matrices. Taking an example,

H5 $$\begin{aligned} \left| 4\right\rangle \left\langle 4\right| =\frac{1}{2} \left( \left| \alpha \beta \right\rangle -\left| \beta \alpha \right\rangle \right) \left( \left\langle \alpha \beta \right| -\left\langle \beta \alpha \right| \right) . \end{aligned}$$

This can be expanded and expressed in terms of single spin density matrices, where for any spin $\left| \alpha \right\rangle \left\langle \alpha \right| =X_\alpha$, $\left| \beta \right\rangle \left\langle \beta \right| =X_\beta$, $\left| \alpha \right\rangle \left\langle \beta \right| =X_-$ and $\left| \beta \right\rangle \left\langle \alpha \right| =X_+$:

H6 $$\begin{aligned} \left| 4\right\rangle \left\langle 4\right| =\frac{1}{2}\left( \hat{X}_\alpha ^1 \hat{X}_\beta ^2 - \hat{X}_{+}^1 \hat{X}_{-}^2 - \hat{X}_{-}^1 \hat{X}_{+}^2 + \hat{X}_\beta ^1 \hat{X}_\alpha ^2 \right) \end{aligned}$$

These basis operators can then be combined with further spins via outer products as required and used as basis matrices $\rho _s$ and $\rho _t$ in relaxation rate computations. These can either be expressed as matrices, or for spin halves, symbolically. Taking the outer product of $\hat{A}_+$ with $\left| 4\right\rangle \left\langle 4\right|$ for example, would be stored for use with the index label ‘p4-4’.

## Appendix I: Code to analyse CH$_3$

A complete set of examples are provided as well as a manual with the software. To illustrate an implementation of RelCalc, the following code calculates all the single quantum carbon auto-relaxation rates for a $^{13}CH_{3}$ system, including external protons and deuterons. We input the parameters, create a basis, image the spin system then generate plots of rates versus $\tau _C$ and different magnetic field strengths, compute a Redfield Kite and assemble tables summarising the main relaxation rates. Specifying the angle as a text string ‘beta’ results in relaxation rates being written in terms of associated Legendre polynomials. First we initialise RelCalc as a class in python, and set some internal variables.

Desired interactions can then be added to the relaxation Hamiltonian. Here we have 3 AX dipolar, 3 XX dipolar and 4 axially symmetric CSAs, one on each atom. Then we have an external proton and an external deuteron, each making dipolar interaction to the 4 central atoms.

Now that the desired interactions have been added to the relaxation Hamiltonian, the Zeeman basis can be built to determine the single quantum carbon relaxation rates, in which all auto and cross-relaxation rates can be evaluated using EvalRate which will subsequently be written to the output LaTex document. We first set the number of degenerate spins to 3, then construct the $A^+X_n$ single quantum Zeeman basis, and evalulate the relataion rates for all combinations of these operators.

We can also compute the irreducible wavefunctions in the point group C3v, compute a set of Liouvillian basis operators from these (IRRBASIS) then compute a complete set of basis operators for 4 spins taking operators with all permutations from +, −, $\alpha$ and $\beta$. We then compute relaxation rates of the irreducible basis operators.

Once these rates have been evaluated symbolically, values can be calculated. We first need supply relevant parameters. In this case, motional parameters are

then values to compute the internal dipolar and CSA tensors

Then inforation to compute the external dipolar interactions

Then set the magnetic field of the spectrometer (here 600 MHz) and compute the parameters from the constants supplied

3D images of the interactions in the molecular frame will be added to visualise all the interactions

Finally, a plot of the auto-relaxation rates for the single quantum carbon rates against rotational correlation time can be generated (as shown in many places in the paper). This is computed at first 600 MHz, then at 1.2 GHz.

Then plot a Redfield Kite at $\tau _c$ of 100 ns

Finally, assemble tables of macromolecular tables for both hopping and diffusive local motion, then conclude the PDF.
